# Changes in Gravitaxis and Gene-Expression in an *Euglena gracilis* Culture over Time

**DOI:** 10.3390/biom14030327

**Published:** 2024-03-09

**Authors:** Julia Krüger, Peter Richter, Julia Stoltze, Binod Prasad, Sebastian M. Strauch, Marcus Krüger, Adeel Nasir, Michael Lebert

**Affiliations:** 1 Gravitational Biology Group, Department of Biology, Friedrich-Alexander University, Staudtstraße 5, 91058 Erlangen, Germanybin.aviansh@gmail.com (B.P.); sebastian.strauch@univille.br (S.M.S.); michael.lebert@fau.de (M.L.); 2 Postgraduate Program in Health and Environment, University of Joinville Region, Rua Paulo Malschitzki, 10-Zona Industrial Norte, Joinville 89219-710, SC, Brazil; 3 Environmental Cell Biology Group, Department of Microgravity and Translational Regenerative Medicine, Otto von Guericke University, 39106 Magdeburg, Germany; marcus.krueger@med.ovgu.de; 4 Research Group “Magdeburger Arbeitsgemeinschaft für Forschung unter Raumfahrt- und Schwerelosigkeitsbedingungen” (MARS), Otto von Guericke University, 39106 Magdeburg, Germany; 5 Univ Brest, INSERM, EFS, UMR 1078, GGB-GTCA, 29200 Brest, France

**Keywords:** *Euglena gracilis*, microarray analysis, culture age, gravitaxis, phototaxis

## Abstract

Age-dependent changes in the transcription levels of 5-day-old *Euglena gracilis* cells, which showed positive gravitaxis, 6-day-old cells without gravitactic orientation, and older cells (9- and 11-day-old, which displayed a precise negative gravitaxis) were determined through microarray analysis. Hierarchical clustering of four independent cell cultures revealed pronounced similarities in transcription levels at the same culture age, which proves the reproducibility of the cultivation method. Employing the non-oriented cells from the 6-day-old culture as a reference, about 2779 transcripts were found to be differentially expressed. While positively gravitactic cells (5-day-old culture) showed only minor differences in gene expression compared to the 6-day reference, pronounced changes of mRNAs (mainly an increase) were found in older cells compared to the reference culture. Among others, genes coding for adenylyl cyclases, photosynthesis, and metabolic enzymes were identified to be differentially expressed. The investigated cells were grown in batch cultures, so variations in transcription levels most likely account for factors such as nutrient depletion in the medium and self-shading. Based on these findings, a particular transcript (e.g., transcript 19556) was downregulated using the RNA interference technique. Gravitaxis and phototaxis were impaired in the transformants, indicating the role of this transcript in signal transduction. Results of the experiment are discussed regarding the increasing importance of *E. gracilis* in biotechnology as a source of valuable products and the possible application of *E. gracilis* in life-support systems.

## 1. Introduction

*Euglena gracilis* is a motile freshwater flagellate, which orients itself in its environment using advanced sensory systems. The cells respond to light, gravity and other external stimuli, such as oxygen concentrations [1,2]. *E. gracilis* shows directed movement towards or away from the vector of light (positive or negative phototaxis, respectively) as well as to the gravity vector (gravitaxis). Phototaxis is triggered by adenylyl cyclases (notably PACα) located in the paraxonemal body (PAB), which is attached to the trailing flagellum of *E. gracilis*. Activation of PACα by blue light leads to an increase in cAMP in the flagellum or cell [3]. Physiological downstream events of cAMP modulation are yet unclear. Gravitaxis was found to be (at least partially) controlled by active physiological signal transduction mechanisms, in which, among others, mechanosensitive ion channels, calcium, cAMP, calmodulin, and protein kinases are involved [4].

In recent years, more methods have become available for researching the movement behavior of *E. gracilis*. In addition, the transcriptome and proteome data of *E. gracilis* are available due to sequencing projects of the recent years [5,6,7,8]. By means of mass spectrometry, Hammond et al. (2021) identified a vast number of proteins in isolated flagella of *E. gracilis* [9]. Among others, various signal-transduction proteins, proteasome complexes, as well as all proteins of a glycolytic pathway, were found. Although *Euglena gracilis* shows significant lifestyle-related adaptations in the flagella, data also show pronounced homology with flagella of other eukaryotes. The *E. gracilis* databases become more and more relevant for detailed investigation of certain pathways [10], genomic identifications [11], or effects of environmental stress on transcriptome [12]. Above all, paramylon, the carbon storage substance (β-1,3-glucan), was obviously found to have positive impacts on health [13]. In order to learn more about carbon-dioxide-related gene expression changes, transcription of genes at CO_2_-concentrations of optimal paramylon accumulation was investigated, and changes in major metabolic pathways were determined [14]. According to the transcriptome data from Ebenezer et al. [8], Krüger et al. [15] developed Agilent-microarrays to perform a large-scale microarray analysis during a parabolic flight to detect gravity-related changes in gene transcription in *E. gracilis*. Due to an elaborated inoculation protocol developed by Stoltze et al. [16], the results of the microarray analysis yielded consistent and reproducible results in independently cultivated samples. The rationale behind the experiments introduced in this manuscript is the simultaneous investigation of gravitaxis as well as changes in gene profile in a developing batch culture.

## 2. Materials and Methods

### 2.1. Cells and Growth Conditions

*Euglena gracilis* KLEBS strain Z (obtained from the SAG, Göttingen, Germany) cells were cultivated in 150 mL complex medium in 300 mL Erlenmeyer flasks [17]. The medium consisted of (per L) the following: 1 g sodium acetate × 3 H_2_O, 1 g pepton, 1 g trypton, and 2 g yeast extract. The inoculum was 3 × 10^6^ cells (determined with a Thoma chamber) per 150 mL of medium for each culture. Cell cultures were maintained at 20 °C under constant light of 25 W/m^2^ supplied with mixed warm-white and cold-white illumination (Solarox, Stendal, Germany).

### 2.2. Image Analysis

Image analysis was performed using an image analysis system, and real-time movement analysis was performed as described elsewhere [18]. In brief, the process was as follows: An aliquot of cells was filled into custom-made cuvettes, which were placed vertically into the optical path of a horizontally aligned microscope, equipped with a digital camera. Full frames of the microscopic pictures were transferred to an attached computer at a frame rate of 20 fps. For each frame, the software detected the position of each object and follows it for 5 subsequent frames. Cells can be selected by their size, excluding detritus or air bubbles. The locomotion of each object was translated into a movement vector. Pooling a defined number of vectors allows for the determination of various movement-related parameters, which in turn indicate the mean movement parameters of an entire cell population [18]. Each measurement took three minutes, and at least three independent measurements were performed with each culture. Parameters displayed in this manuscript are (1) circular histograms, which indicate the movement directions of all recorded vectors. The number of cells swimming in a given direction is indicated by the length of a corresponding section on the histogram. (2) The velocity indicates the average speed of all recorded motile objects during a measurement. (3) Motility gives the number of motile cells in a culture. (4) Alignment indicates whether cells swim preferably in a horizontal direction (alignment values < 0), a vertical direction (alignment values > 0), or without orientation (values close to 0). (5) The direction is the fraction of cells swimming upward minus the fraction of cells swimming downward. Negative values indicate downward swimming (positive gravitaxis), and positive ones are upward swimming (negative gravitaxis). (6) 120° upwards shows the fraction of cells swimming upward in a cone of 120°. Higher values indicate negative gravitaxis. In addition, the software also analyzes the shape and size of a cell. (7) The form factor is the ratio of the squared circumference of an object divided by its area and normalized to a circle value and indicates whether cells are more rounded (form factor close to 1) or more elongated (higher values). (8) The *r*-value indicates the precision of orientation of cell culture and ranges between 0 (no orientation, cells do not show significant common movement direction) and 1 (high orientation, all cells of the culture swim in the same direction) [19].

### 2.3. RNA Isolation

Cell samples in their liquid media were directly fixed using TRIzol^®^ (ratio 1:6 of mediaTRIzol^®^, Life Technologies, Carlsbad, CA, USA) to prevent any possible changes in transcription due to centrifugation or filtration, respectively. Subsequently, chloroform was added to the samples (0.2 mL for each mL of TRIzol^®^). After 15 min incubation at room temperature, samples were centrifuged for 20 min at 4 °C and 12,000× *g*. The upper aqueous phase was transferred into a fresh reaction tube, and RNA was precipitated by the addition of an equal volume of isopropanol. After incubation at room temperature, RNA was pelleted (15 min at 4 °C and 12,000× *g*) and washed twice with 75% ethanol (5 min at 4 °C and 75,000× g). After drying at 50 °C, RNA was resuspended in RNAse-free water. RNA quality was checked photometrically using a NanoDrop™ lite spectrophotometer (Thermo Fischer Scientific, Waltham, MA, USA) and the Agilent 2100 Bioanalyzer (Agilent Technologies, Santa Clara, CA, USA) according to the Agilent Technologies RNA 6000 Nano Assay protocol.

### 2.4. Synthesis of cDNA and Fluorescent Complementary RNA (cRNA)

Synthesis of fluorescent cRNA needed for microarray hybridization was realized by the Low Input Quick Amp Labeling Kit (Agilent Technologies, Santa Clara, CA, USA) according to the manufacturer’s instruction. Before cDNA synthesis, spike-in RNA (One-color RNA Spike-In Kit, Agilent Technologies, Santa Clara, CA, USA) was added to the isolated RNA (2 µL per 100 ng RNA). After the addition of a T7 primer mix, samples were denaturized (10 min at 65 °C). After the addition of 5× first-strand buffer, 0.1 M DTT, 10 mM dNTP mix, and Affinity Script RNase Block Mix, subsequent reverse transcription was performed at 40 °C for 2 h followed by heat inactivation of the enzyme (15 min at 70 °C). The cDNA served as a template for in vitro transcription of cRNA with Cy3-labelled CTP (2 h at 40 °C). After in vitro transcription, cRNA was column purified (RNeasy Mini Kit, Qiagen, Hilden, Germany), and the concentration and labeling efficacy of each sample were checked with a UV-VIS spectrophotometer (NanoDrop Technologies, Inc., Wilmington, DE, USA).

### 2.5. Microarray Hybridization

Microarray development and validation of the method was described earlier [15]. In brief: For determination of the transcription levels, custom-made Agilent-microarrays were employed, which were designed with the online design tool eArray for SurePrint G3 Custom GE 8 × 60 K microarrays (Design ID 084219, G4102A, Agilent, Santa Clara, CA, USA) using a transcriptome database for *E. gracilis* [8]. Slides with microarrays were produced by Agilent Technologies. One printed slide contained eight arrays with 20,396 different transcripts each (specific 60-mer oligonucleotides) in triplicate and various Euglena-specific replicates and technical controls [15]. Hybridization was performed as described earlier [10] using the Gene Expression Hybridization Kit (Agilent Technologies, Santa Clara, CA, USA). After the fragmentation of cRNA (about 600 ng of cRNA per sample), 40 µL of samples were loaded onto the arrays and hybridized in a hybridization oven (G2545A, Agilent Technologies, Santa Clara, CA, USA) for at least 17 h at 65 °C, at a rotational speed of 10 rpm. Subsequently, slides were washed with wash buffers and dried in an air stream for scanning with an Agilent C microarray scanner (G2565CA, Agilent Technologies, Santa Clara, CA, USA). An SHG-YAG laser (emission: 532 nm) served for excitation and was combined with an emission filter (550–610 nm). The individual fluorescence signals during a scan were transformed into a 16-bit TIFF file, showing the spatial distribution of the signal intensities. Scanner settings were as follows: Channel Green, resolution 3 µm, scan region Agilent HD 61 × 21.6 mm, TIFF 16-bit, dynamic range 100–10%. Microarray scans were analyzed using the Agilent Feature Extraction software (version 11.5.1.1, Agilent Technologies, Santa Clara, CA, USA) with the following settings of the extraction protocol: Extraction protocol: GE1_1105_0ct12, Grid 084219_D_F_20160803, Background method: No Background, Background detrend: On, Multiplicative detrend: True.

### 2.6. Agilent Microarray Data Analysis and Transcript Annotation

Microarray data were processed with GeneSpring 14.8 GX software (Agilent Technologies, Santa Clara, CA, USA) to normalize (quantile normalization) the data and perform the quality assessment and fold change analysis. For hierarchical clustering, ANOVA (one-way) statistical analysis was performed and displayed using the Pearson uncentered (absolute) correlation and Wards linkage rule. Fold changes were calculated using a moderated *t*-test. The *p*-value computation was performed asymptotically as data were assumed to show normal distribution. Cut-off values for the fold change and *p*-value were set to 1.5-fold and 0.05, respectively. Each condition was compared to the day 6 reference sample which did not show gravitactic behavior. Four independent samples were evaluated. Blast2GO pro software (version 4.1.9, BioBam Bioinformatics, Valencia, Spain) was employed for transcript annotation. This software autonomously performed Blast and InterProScan searches and extracted Gene Ontologies (GO) and corresponding enzyme codes (EC). Search in Blast was performed with translated protein sequences of the corresponding transcripts. Results of Blast and InterProScan were linked to GO terms from the non-redundant reference protein database (PIR). Enzyme codes were assigned based on GO annotation and visualized via KEGG pathway analysis. Gene set enrichment analysis was performed to find regulated functional groups. The number of DEG of a same GO term was correlated with the total number of genes classified to the same GO term. The published databases provided in Ebenezer et al. 2018 (Supplementary S2 “proteome”) [8] and Cordoba et al. 2021 (Supplementary S2) [7] served as references.

## 3. Results

### 3.1. Movement Behavior and Gravitaxis of Euglena gracilis Cells Concerning Culture Ages

**Figure 1:**
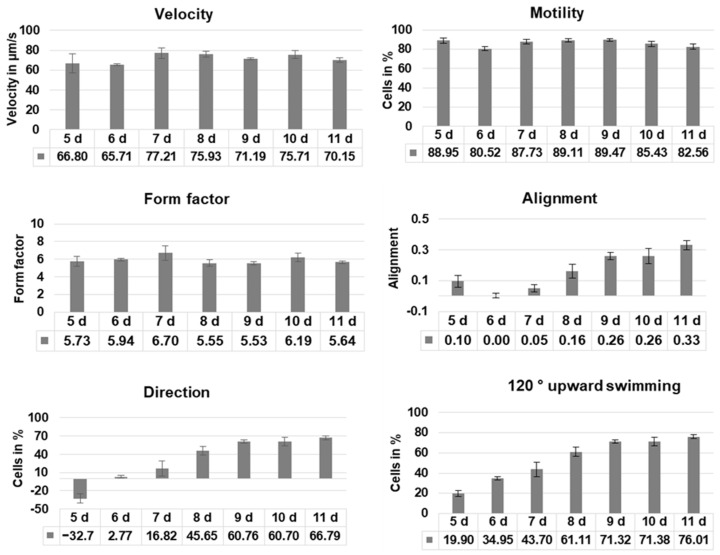
Swimming behavior of *Euglena gracilis* cells depending on culture age. Cells of four independent samples were analyzed using computer-based image analysis, and the results were pooled. Before measurements, cells were incubated in the dark for 60 min. Velocity: average speed of the cells, motility: average percentage of moving cells, form factor: indicates if a cell is circular (1) or elongated (>1), alignment: indicates whether cells swim horizontally (negative values), show no preferred direction (values close to 0), or vertically (positive values), respectively to the gravity vector, direction: fraction of cells swimming upward—fraction of cells swimming downward, 120° upward swimming: cells swimming upward in a cone of 120° around the gravity vector.

**Figure 2:**
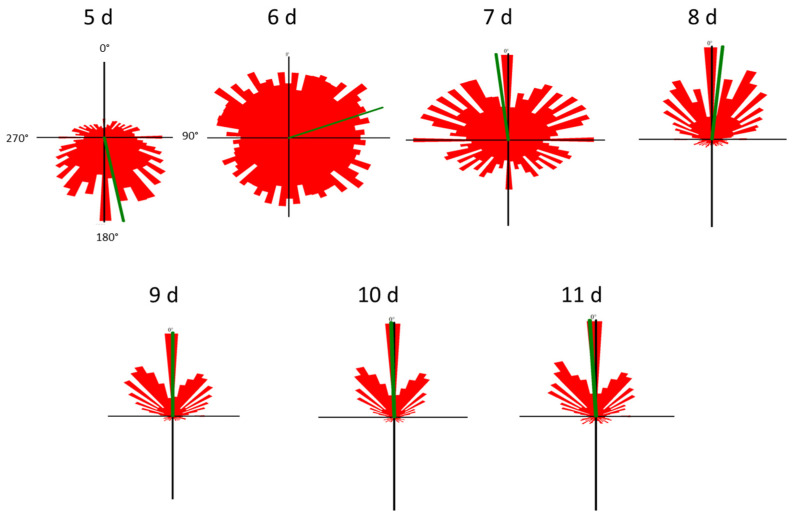
Movement histograms of *Euglena gracilis* cells of different cell culture ages. The length of each sector represents a fraction of cells swimming in this direction. The mean swimming direction of a culture is represented by the green line. The angles are depicted in the first histogram (5 d). Cell movement was recorded using a horizontally oriented microscope, which means that 0° is upward, 180° is downward and 90° and 270°, respectively, are sideward.

Cell motility, cell form, and velocity did not show significant changes in the course of cultivation. During the initial culture period (until day 5), cells in all four samples showed positive gravitaxis. At day 6, the average orientation of the cell cultures became random (no preferred movement direction of the cells). After seven days of cultivation, all samples developed a more and more pronounced negative gravitaxis. After 9 days, the precision of negative gravitaxis (i.e., values alignment and 120° upward swimming) reached a plateau and did not further change significantly (Figure 1 and Figure 2).

### 3.2. Culture-Age-Related Gene Expression Changes

**Figure 3:**
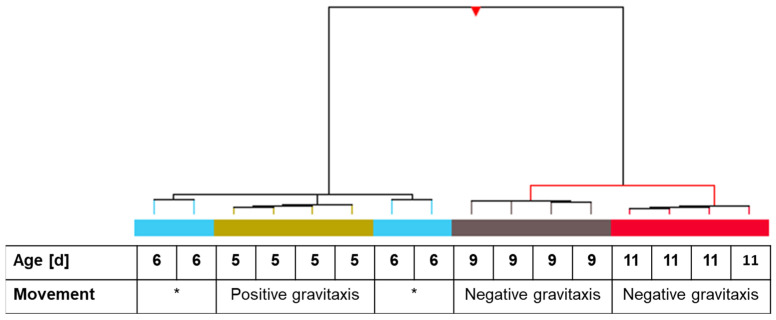
Hierarchical clustering of individual samples of *Euglena gracilis* cells at different cultivation times in correlation with gravitactic orientation. One-way ANOVA analysis of the samples was applied before clustering. Hierarchical clustering was applied using Pearson uncentered for similarity measure and Wards linkage rule as distance metrics. Related independent samples are indicated with colored bars. The table under the dendrogram shows the age and gravitactic behavior of the particular samples. Asterisk indicates random movement of the culture.

Microarray hybridization was performed with RNA extracted from 5-, 6-, 9-, and 11-day-old cells. One-way ANOVA variance analysis and visualization employing hierarchical clustering revealed that all four independent samples of days 5, 9, and 11 clustered together. The 6-day culture, which showed no particular gravitactic behavior, was divided into two clades (Figure 3).

**Figure 4:**
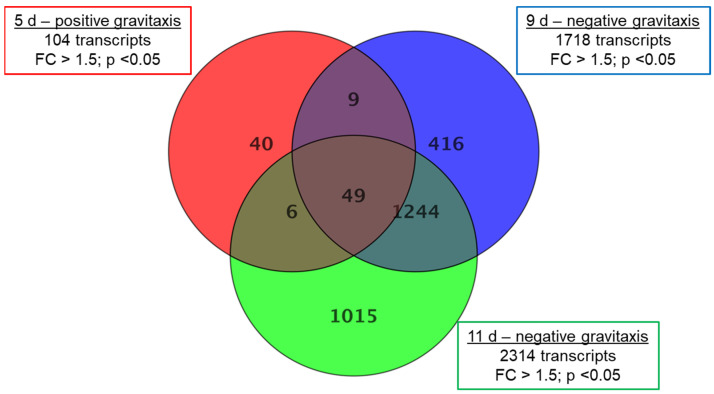
Gene expression changes in *Euglena* cell cultures at different days of growth. Samples after 5 (positive gravitaxis), 9 (negative gravitaxis), and 11 days (negative gravitaxis) of cultivation, were compared with samples of six days old cells (random movement). Significantly differentially expressed genes are presented in the corresponding circles. The numbers in the intersections of the circles stand for changes, which were also observed in other sample data. Fold changes were calculated by moderated *t*-test and regarded as significant when equal or greater than 1.5-fold, the *p*-value cut-off was set to 0.05, and for false discovery rate (FDR) was accounted by Benjamini-Hochberg correction.

For further comparison, samples of day 5 (positive gravitaxis), 9, and 11 (negative gravitaxis) were normalized against the randomly oriented sample 6 d. The analysis revealed 2779 differentially regulated transcripts in total, where only 104 (40 specific) transcripts were different on day 5, while 1718 (416 specific) and 2314 (1015 specific) transcripts were found to be differentially regulated on days 9 and 11, respectively (Figure 4).

In 5-day-old cells, a relatively low number (104, from which 40 were specific for this culture age) of differentially expressed transcripts (compared to day 6) were found. In addition, the fold changes were considerably lower. Most of the changes were found to be in the range of 1.5 to 2, and only in 11 transcripts, the fold-change was greater than 2. Older samples (9 days and 11 days of culture, respectively) showed more (1718 (416 specific) and 2314 (1015 specific), respectively) differentially expressed genes as well as a larger range of genes with more than threefold expression changes (123 and 275, respectively). Highest changes were observed in samples after 11 days of culture, where fold-changes of up to 15-fold were measured (Supplementary S1). From the total of 2779 different transcripts that were differentially expressed in at least one sample, about 20–40% were specific at the corresponding culture age. Almost half (40 of 104) of the differentially expressed transcripts found on day 5 were also found to be changed on days 9 and 11, respectively. However, the gravitactically negative samples of day 9 and day 11 share a considerably higher number (1244) of differentially expressed genes (Table 1). Although the number of up- and downregulated transcripts is almost the same. However, almost all pronounced differentially expressed genes (FC ≥ 5) are downregulated.

### 3.3. Gene Set Enrichment Analysis (GSEA)

The 2779 differentially expressed transcripts were annotated with Blast2GO and evaluated using gene set enrichment analysis (GSEA). Gene set enrichment analysis indicates differences in biological functions or pathways between two biological samples (complete lists in Supplementary S2 and S3). The gene set enrichment analysis of differentially expressed transcripts from samples of day 5 and day 6 only revealed changes in DNA-binding processes (Table 2). The gravitactically negative samples of day 9 and day 11 were found to differ in a high number of GO terms compared to day 6, with the highest number of significantly different GO terms on day 11. Among others, DNA binding, changes in mitochondrial genes, genes of the plasma membrane, signal transduction, ATPase activity, and cyclic nucleotide were found to be altered (Table 2). Intracellular signal transduction processes were impacted and above all, nucleotide biosynthetic processes. Interestingly, fold changes linked to the corresponding adenylyl cyclases showed that they were mainly upregulated. Changes in RNA metabolic processes probably indicate changes in DNA replication, as well as changes in DNA repair and metabolic processes. On day 11, changes in photosynthetic and chromosomal genes were observed additionally, to set the number of differentially expressed genes in perspective to the whole genome. GO terms were retrieved from Ebenezer et al. [8] and Cordoba et al. [7]. However, in the recent database of Cordoba for most GO terms far more transcripts were found, provided in Table 2 for comparison (Data of Ebenezer and Cordoba with respect to observed changes are provided in Supplementary S4). For some GO terms, a considerably fraction of transcripts were found to be changed. Day 5 and 6 differ in the expression of proteins involved in DNA-binding (GO:0003677) of about 1.41%. However, at day 9 and day 11, the differences changed up to 16.9% or 17.46%, respectively. About 35.48% or 43%, respectively, of transcripts involved in cyclic nucleotide biosynthetic process (GO:0009190) were differentially expressed at day 9 or day 11 compared to day 6 and 43% and about 36.5% or 45.16% transcripts coding for proteins involved in phosphorus–oxygen lyase activity (GO:0016849). Other gene ontologies which were considerably different from 6d-gene expression were mitochondrion (GO:0005739-day 9: 3.93%, day 11 3.35%), ATPase activity (GO:0016887-day 9: 8.1%, day 11 9.72%), and intracellular signal transduction (GO:0035556-day 9: 8.22%, day 11 10.27%).The GO term photosynthesis (GO:0015979) differed only in the 11 d samples (19.64%).

### 3.4. Changes of Functionally Annotated Transcripts in Euglena gracilis

**Figure 5:**
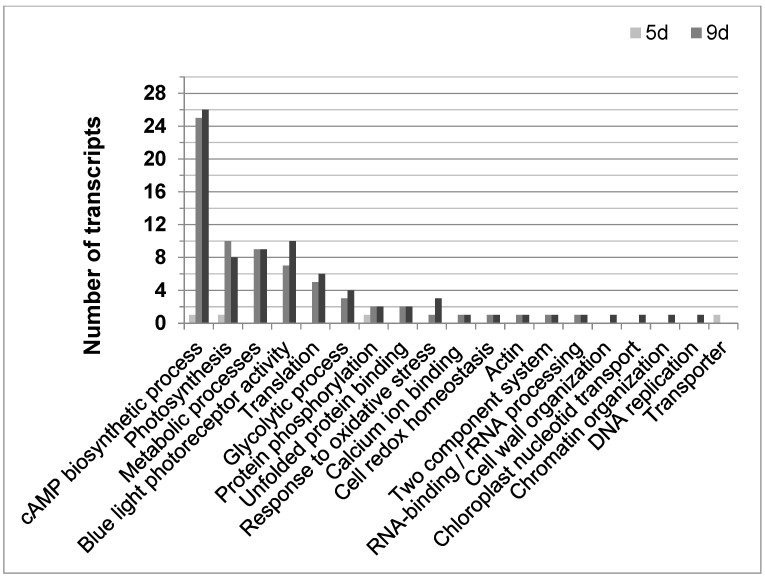
The distribution of functional annotated transcripts in Euglena gracilis was significantly regulated during different cell culture ages with opposing gravitactic orientations. Graph shows the number of functional annotated transcripts for their corresponding GO term for 5 days cultures (5 d, light grey bar), showing positive gravitaxis, 9 days cultures (9 d, medium grey bar), and 11 days cultures (11 d, dark grey bar), both showing negative gravitaxis). Transcripts showed fold changes ≥1.5-fold and *p*-values ≤ 0.05 as calculated by moderated *t*-test and asymptotic *p*-value computation.

Until now, 108 of the 2773 differentially expressed transcripts could be annotated according to their function in *E. gracilis*. Most changes compared to not gravitactic cultures (day 6) were found in older cultures (9 or 11 days old, respectively) showing negative gravitaxis (Figure 5). Above all, genes involved in the cAMP biosynthetic process (GO:0006171), determined by sequence similarities to putative membrane-bound adenylyl cyclases (BAD20740.1 and BAD20741.1), were found to be altered. Interestingly, 25 and 26 of these genes were upregulated in cell cultures at day 9 and day 11, respectively. However, only 10 of the corresponding sequences were identical. In most cases, the adenylyl cyclases were found to be upregulated compared to transcription levels in not oriented cells (6 days). Only four transcripts were found to be downregulated. In young cells (5 days), which displayed a gravitactically positive behavior, only one gene involved in the cAMP biosynthetic process was differentially expressed (downregulated) compared to the 6-day-old cultures. Moreover, various transcripts meeting the criteria of the GO term photosynthesis (GO:0015979) were found to be differentially expressed, in particular, in the older cultures exhibiting negative gravitaxis (10 transcripts in 9-day-old cultures and 8 in 11-day-old cultures). Transcripts coding for light-harvesting complex II were mainly downregulated, except for four transcripts, which were upregulated. Young positively oriented cells (5 days old) did only differ in one transcript related to photosynthesis compared to the 6-day reference. In addition, the number of differentially expressed transcripts involved in metabolic processes (GO:0008152) was higher in old cultures, while no differences were found between 5-day-old cultures and the 6-day reference. Among other processes, cellular amino acid metabolic process, glycerolipid biosynthesis, carbohydrate metabolic processes, porphyrin-containing compound biosynthetic process, and pyruvate metabolic process were found to be altered in older cells. Only older cells (9 and 11 days, respectively) showed a differential expression in genes involved in glycolytic processes (GO:0006096, mainly upregulated) as well as in genes involved in blue light photoreceptor activity (GO:0009882, mainly upregulated). Among the altered blue light photoreceptor transcripts isoforms of the *E. gracilis* photoreceptors, PACα and PACβ were identified.

Several transcripts accounting for different GO terms were also found to be changed but only to a smaller extent. A complete list with all sequences, fold changes, and annotations can be found in the Supplementary Data (S1 and S2). Protein sequences are provided in Supplementary S5.

### 3.5. Single Transcript Analysis of Sequences Differentially Regulated in Cell Culture, Showing Negative or Positive Gravitaxis, Respectively, Compared to Not-Oriented Cells

Single transcript analysis was performed for the 49 genes, which were found to be differentially expressed at all days (Figure 4). Based on the GO terms, a total of 16 gene functions could be assigned while the rest resulted in ambiguous findings (Table 3). In terms of signal transduction mechanisms, two adenylate cyclases (EG_transcript_1009 and EG_transcript_11359, Supplementary S4) and a cyclic nucleotide-binding protein were detected. Compared to day 6, all of these proteins were found to be downregulated at day 5 and upregulated at day 9 and day 11. Transcript levels increased from day 5 to day 9 and subsequently remained stable on day 11. A similar pattern was found in the transcription of a potential flagellar calcium-binding protein (EG_transcript_26861), which was found to be downregulated at day 5 (1.51-fold) and upregulated at days 9 (2.16-fold) and 11 (2.27-fold), respectively. Transcription levels of phosphatidylinositol 4 kinases (EG_transcript_10359) as well as of potential major facilitator transporters, which enable chemiosmotic transport of small solutes (EG_transcript_9532, EG_transcript_8497) were upregulated at day 5 and downregulated at day 9 and 11, respectively. Similarly, a transcript with homology to a cyclopropyl isomerase in *Guillardia theta* (EG_transcript_19556) was also found to be upregulated at day 5 and downregulated at day 9 and 11, respectively. This transcript was found to be possibly involved in gravitropism in *Arabidopsis thaliana*.

## 4. Discussion

Transcription levels of four independent *Euglena gracilis* cultures were determined. Hierarchical clustering revealed that expression levels were very similar at the same cell age, proving the reproducibility of the culture conditions. An exception was cells at the transition state between positive and negative gravitaxis, which were clustered in two groups. Shortly after inoculation, *E. gracilis* cells are located at the ground of the flask and do not show free movement (unpublished observation). Under laboratory conditions with a moderate ambient light environment, cultures show negative gravitactic behavior (swim upward in the water column) about one week after inoculation. However, cells of freshly inoculated cultures initially show positive gravitaxis (swim towards the bottom of the flask); after some days, no preferred swimming direction of the cells of a culture can be observed (random distribution) before most cells of a culture switch to negative gravitaxis [20]. Six-day-old cells showing random orientation were employed as a reference for cell ages with clear positive or negative gravitaxis because they undergo a transition of the gravitactic behavior (gravitactic sign-change).

In total, more than 2700 differentially expressed transcripts were detected at different culture ages compared to the 6-day-old culture, making interpretation of the data very complex. The gravitactically positive cells at day 5 showed more similarities to day 6 compared to older culture age cells (9 and 11 days, respectively). This is in good coincidence with earlier studies, where metabolic changes, changes in the activity of hydrolytic enzymes, and changes in chromatin structure as well as chlorophyll a/b ratios with increasing cell age were reported [21,22,23,24]. The cultures were batch cultures with no fresh medium supply in the course of cell growth. That means that with proceeding culture age, nutrients necessary for heterotrophic growth decrease while cell number and self-shading effects increase. These factors significantly change growth conditions, and the observed alterations in transcription pattern might account for adaptation to the changing environment. Another factor affecting cells and cell movement is the density of the medium. Gravitaxis was found to be impaired with increasing density of the growth medium [25]. However, no culture-age-related changes in specific densities of growth media have been observed until now. Under the given inoculum (20,000 cells/mL), light, nutrients, and temperature conditions, it was found that after 5 days, cells are still in the exponential growth phase, while after 6 days, cell number becomes maximal and subsequently almost stays unchanged (about 10^6^ cells/mL), indicating transition into the static phase (Stoltze, unpublished data). *E. gracilis* was described to grow heterotrophically, photoheterotrophically, or photoautotrophically [26]. The cells are not able to utilize nitrate as a nitrogen source and therefore metabolize various carbon sources such as amino acids [27,28]. It was found that accessible nitrogen in the medium drastically decreases with increasing number of cells [28]. This changes nitrogen availability for the cells with increasing culture age making expression of new genes necessary. The observation that 5-day- and 6-day-old cultures differ less in gene expression may account for a transition from preferred heterotrophic to phototrophic growth (changes in photosynthesis-related genes as well as signal-transduction). In addition, higher cell numbers result in increased self-shading of cells and decreased light availability. Changes in the GO term glycosyl transferase activity in combination with changes in GO term “plasma membrane” is a hint for modification of the cell membrane. This is in accordance with the finding that *E. gracilis* possesses a vast majority of surface glycans [29]. In contrast, actin mRNA was reduced in older cell cultures which is most likely a result of the reduction in cell size found in older cell cultures (unpublished observation). Decreased mRNA content for fructose-1,6-bisphosphate aldolase and enolase indicate a lower metabolic activity in older cells probably due to stationary growth phase and reduced cytokinesis. Another factor which might contribute to that is depletion of nutrients in the medium. The generation of reactive oxygen species (ROS) is probably different for the various compartments in older cell cultures as different ascorbate peroxidases showed up- or downregulations.

EG_transcript_19556 shows similarity to a cyclopropyl isomerase in *Guillardia theta* and was found to be slightly upregulated in cells showing positive gravitaxis and downregulated in cells oriented gravitactically negative. Changes in the transcription level of EG_transcript_19556 were also found in other experiments where cells were subjected to altered acceleration conditions, e.g., in the course of a parabolic flight experiment [19]. Detailed sequence analysis of this protein revealed homology to CPI1 in *Arabidopsis thaliana* (AT5G50375), a protein involved in sterol biosynthesis. In higher plants, certain sterols in the membrane were found to be involved in the polar distribution of PIN2 in [30]. Mutations of CPI1 led to changes in the sterol composition that interfered with PIN2 endocytosis and harmed root gravitropism [31]. Downregulation of EG_transcript_19556 resulted in impaired gravitaxis as well as phototaxis. Also, knock-down with CRISPR Cas9 indicates impaired orientation. However, these data still need to be confirmed. The possible role of EG_transcript_19556 in *E. gracilis* is still unclear. Other observations like an altered cell shape and abnormalities in perception of light and motility might indicate an important or rather general role for *E. gracilis*.

The data show that microarray analysis is a promising tool for the determination of culture-condition effects on cells. This has become important because in recent years, *E. gracilis* was found to exert very interesting health effects. Among others, *E. gracilis* produces the storage carbohydrate paramylon [32,33], vitamin E as well as polyunsaturated fatty acids (PUFAs) [34]. During a study with an arthritis mouse strain (DBA/1J), oral uptake of *E. gracilis* or the storage polysaccharide paramylon resulted in a strong reduction in clinical arthritis symptoms [35]. In addition, a decreased secretion of inflammatory cytokines IL-7, IL-6, and IFN-γ was observed. The authors suggested that paramylon regulates T helper 17 cells. Recently, Piovan et al. (2021) reported about promising effects of *E. gracilis* extracts against LPS-induced neuroinflammation by modulation of NF-kB and Nrf2 pathways [36]. In lung carcinoma cells, *E. gracilis* extracts show possible antitumor effect by attenuation of suppressor cells of the immune system (granulocytic myeloid-derived suppressor cells, gMDSC) [37]. As *E. gracilis* was determined to be the fastest-growing *Euglena* species [38], it is believed to serve as a very promising organism in realizing the “5 Fs“ (food, fiber, feed, fertilizer, fuel) [38,39,40]. Cultural conditions have a strong impact on the performance of *E. gracilis*. He et al. investigated metabolites under stress conditions (cadmium, the antibiotic paromomycin, as well as nitrogen starvation) [41]. Significant changes in the metabolite profile were found even if no physiological effects of the cells were visible. Schwarzhans et al. showed that defined culture conditions (nitrogen content, presence of peptone) were crucial to achieving optimal n3- and n6-polyunsaturated fatty acid (PUFA)-ratios [21]. Under anaerobic conditions, *E. gracilis* cells accumulate wax-esters [42]. Modification of the growth medium by replacing yeast extract, tryptone, and peptone with a single amino acid (glutamic acid, glutamate, or asparagine) resulted in the production of euglenatides, a new class of cyclic peptides with a strong inhibitory effect on pathogen fungi as well as a breast cancer cell line [43]. Transcript analysis of cells during improved culture conditions, which result in the accumulation of a desired product, will help to understand the underlying mechanism enabling further modification of the culturing process. Using *Agrobacterium tumefaciens*-mediated transformation [44,45] or the application of CRISPR Cas9 [46] or CRISPR Cas12a [47], promising synthesis pathways can now be genetically modified.

*E. gracilis* is a promising component for life support systems in space research and has already been employed in space experiments [4]. With *E. gracilis* as an intrinsic part of a long-term closed biological life support system, it was possible to grow tomatoes from seed to seed [48]. With a view to *E. gracilis* in biological life support systems in manned space flight, determination of growth and aging of *E. gracilis* cultures is important.

## 5. Conclusions

The gene expression and phenotype of the *Euglena gracilis* cells are changing according to culture age. In terms of the significance of this organism for biotechnology, pharmacology, and space applications (e.g., biological life-support systems), determination of such shifts is very important.

## Figures and Tables

**Table 1:** Changes in gene expression with respect to culture age. Samples after five (positive gravitaxis), nine (negative gravitaxis), and eleven (negative gravitaxis) days, respectively, were compared with gene expression of samples of day six. The table shows several differentially expressed genes classified according to their fold-change (FC). Fold changes were calculated by moderated *t*-test and regarded as significant when equal or greater than 1.5-fold, the *p*-value cut-off was set to 0.05, and FDR was accounted for by Benjamini-Hochberg correction. Up/down: ratio up- and downregulated genes.

Fold Change	5 d	9 d	11 d
FC ≥ 1.5	104	1718	2314
FC ≥ 2.0	11	526	758
FC ≥ 3.0	0	123	275
FC ≥ 5.0	0	9 (all downregulated)	98 (mainly downregulated)
Up/down	44/60	805/913	1115/1199

**Table 2:** Analysis of differentially expressed genes according to GSEA (gene set enrichment analysis). Samples after five (positive gravitaxis), nine (negative gravitaxis), and eleven (negative gravitaxis) days, respectively, were compared with gene expression of samples of day six. The threshold for gene set size was set to 5, nominal *p*-value cut off ≤ 0.05, and false discovery rate (FDR) ≤ 0.25. All annotations and calculations were performed with Blast2GO. The right column shows all identified genes of a corresponding GO term identified by Cordoba et al. 2021 [7].

Cell Culture Age	GO ID	GO Name	Number of Transcripts	Total Number of Corresponding GOs [7]
5 d vs. 6 d	GO:0003677	DNA binding	5	355
9 d vs. 6 d	GO:0003677	DNA binding	60	355
	GO:0005739	Mitochondrion	41	1044
	GO:0005886	Plasma membrane	11	1395
	GO:0009190	Cyclic nucleotide biosynthetic process	33	93
	GO:0016070	RNA metabolic process	25	720
	GO:0016779	Nucleotidyltransferase activity	10	84
	GO:0016791	Phosphatase activity	9	159
	GO:0016849	Phosphorus-oxygen lyase activity	34	93
	GO:0016874	Ligase activity	14	255
	GO:0016887	ATPase activity	35	432
	GO:0035556	Intracellular signal transduction	36	438
	GO:0065008	Regulation of biological quality	11	723
11 d vs. 6 d	GO:0002376	Immune system process	6	401
	GO:0003677	DNA binding	62	355
	GO:0005694	Chromosome	8	294
	GO:0005739	Mitochondrion	35	1044
	GO:0005886	Plasma membrane	16	1395
	GO:0006281	DNA repair	6	268
	GO:0009190	Cyclic nucleotide biosynthetic process	40	93
	GO:0015979	Photosynthesis	11	56
	GO:0016070	RNA metabolic process	26	720
	GO:0016757	Transferase activity, transferring glycosyl groups	43	121
	GO:0016779	Nucleotidyltransferase activity	14	84
	GO:0016849	Phosphorus-oxygen lyase activity	42	93
	GO:0016887	ATPase activity	42	432
	GO:0035556	Intracellular signal transduction	45	438
	GO:0042578	Phosphoric ester hydrolase activity	11	190
	GO:0042592	Homeostatic process	15	313

**Table 3:** Determination of age-dependent expression changes in *Euglena gracilis* cultures. The table shows transcripts, which were differentially expressed in young positive gravitactic cells (5-day-old cultures) as well as in older negative gravitactic cells (9 or 11-day-old cultures, respectively), compared to 6-day-old cultures, which show no gravitaxis. Allocated transcript functions and domains as well as several corresponding transcripts are shown. Annotations were determined by BLAST2GO or manual Blast search. NA: no similarities to known proteins, (GO) transcripts without conserved domains, or unspecific similarities. The threshold for similarity measure (E-value) was set to 1E-03 for all annotations.

Function	Number of Transcripts
Cell cycle	1
Dephosphorylation	1
Flagellar protein	1
Hydrolase activity	1
Meiotic cell cycle	1
Metabolism	1
Nucleotide metabolism	1
Positive gravitropism	1
Retrotransposon nucleocapsid	1
RNA-binding	1
Signal transduction	4
Transport	2
No GO	9
NA	24
Total	49

## Data Availability

The large-scale data of microarray analysis have been deposited into ArrayEx-press. All raw data and detailed test protocols will be provided upon request.

## Supplementary Materials

The following supporting information can be downloaded at: https://www.mdpi.com/article/10.3390/biom14030327/s1, Supplement S1: This supplement presents the influence of *Euglena gracilis* culture age on gene expression, elucidated through gene array analysis. It encompasses the total count of differentially expressed genes, categorized based on fold-change ranges, in *Euglena gracilis* cells at distinct culture ages (5 days, 9 days, and 11 days) when compared to the baseline of six days. Additionally, Supplement S1 provides comprehensive raw data for all identified differentially expressed genes (DEGs) and a ranking of genes based on their respective fold changes (FCs). Supplement S2: This section compiles all transcripts identified as differentially expressed in *Euglena gracilis* across the three investigated culture ages (5 days, 9 days, and 11 days) in comparison to the 6-day samples. Supplement S3: Possible functions of the proteins encoded by the DEGs (differentially expressed genes), which were differentially expressed for the corresponding days. Supplement S4: Correlation of GO-terms of DEGs related to culture age in *Euglena gracilis* with total numbers of identified genes with same GO-term which were published in [7,8]. Supplement S5: This section provides the amino acid sequences of proteins that exhibit differential expression in relation to the culture age of *Euglena gracilis*.
