# Supplementary material for: Changes in Gravitaxis and Gene-Expression in an Euglena gracilis Culture over Time
Source: Biomolecules. 2024 Mar 9;14(3):327. doi: 10.3390/biom14030327 (PMC10967724; doi:10.3390/biom14030327)
Supplement: Supplementary file 1 [file biomolecules-14-00327-s001.zip › Supplement S5.pdf]

Supplement S5: Proteome sequence of corresponding transcript number

TK-Nr.	Proteom-Sequenzen
9	MVQSFGSESRFTAEQQDLRPFAIRRGNAEVLLFIPTDVWQTAAHLRQRFLEDNKADSEKTSAELALEFLQFLVNTDSREEEAVRFVRFLSLVVQYCYEEVFKNNNVHCADLSRF<br>TSVTAPTLIQNWYKATHRLQQSKVAVPKPPSALFEAAGRGTVTLGALFGGQGPDWLRPLRELHATYQPVVEPFLRAMARKLNAQAASVEARASCLLLYGLDFMGWLEGGAA<br>LPPDQYLTSAPVNLPLIGVVQLANYWVMWKSLDRPLQEVQSAFKGAAGHSLGIISAVVLASSADEAEFLQNAATGITLLLWIGLRASVAYPAVALDPDVLEDSLTANEGKPTPM<br>LSIAKLPISAVQQHVEEINRLVPADRQVELALVNGPRNVVVSGPEDSLCGLNRMLRKLKVQASKDQSRVPHSKRVAPLATTFIQSSAPFHSSYLTAAVEMVAADIDRLGLRFPLK<br>AAHPVLSTADGTPLSFSPDLPLTLARLTLVSKVDWPKSSALPVSHLIDFGPSGMGLLTHRNKEGTGVQVILAGELTAPTPAVLPKAALFDALPDHLRWAPNWEEEYRPRLVRTA<br>HDGRLHIDTPFSRLVGKPPLMVAGMTPTTGSTAFVAATVSAGYHVELAGGAYFSEPGIRGAVQNILTRTPAGESITMNLLFLNPFLWNLEFPLALKMRKEGIPLNGLTVAAGVP<br>SLDVADTILDAVHEAGMRHISFKPGAVEAIFTVCSIAERHPDVSVILQWTGGRAGGHHSFEDVDQPILTSYATIRKHRNIILVAGSGFGDAAGSVPYLTGEWSTKYDYPAMPFD<br>GILMGSRCMVAKEAQGSDGCKELIAKAEGVLDPKNWEKSYDGPVGGVVTVTSELGEPIHKIATRGTLFWKELDTKVFSLPKEKRLAKLLEMKPYIIQRLNADYPKPWFPKKLNG<br>EQPAEGYCELWDMTYYEIALRLVEVLYHPHIERWLDVTMRNLTADWLDRIEERFVGGDQRPRVLQRRAQLDAPQTFLEQFFAQYPAAMTQRVQTADWQHFVHLSRRKTQ<br>KPVPFILMFDDEFLLWFKKDTLWQSENLAMVPGQDADRVCILQGPVAAAHSRVVNQPIKELLDDIYEGQVRLLLDHYYGGDEGQVPTVEMLGGPPPVASVPSLPGVIVAERT<br>DDVQVFQLASAGPLPGSAEWRRFVAPREANWLQALLTQPHIQRGTRLAPNPLGRVLAPRHGQRLKVCYTGQMPTGWALYDDDYMKDVVVDGEISPVVKCQYDGRDLITLR<br>MLEPLGASAAECVFQFRYVRTCGPNPIHEVMEDRNARIKQFYSALWFGPGDTGAVDAQADGAPWEIKDLLTCTHKVTRAEITDFCRAVGNVSEHHVDRGQAVLQAPMDFA<br>MVVGWRAVIRALFPQCVDGDLFQLVHLSNGFHLYDDCRPLAEGDEVATRGAITGLTFKDGNMTVEVEATLAREGVDFMTITSAFLYRNAQGSDAHAFQYTRDTPVQVQVR<br>DARDVAVLLNKPWIHFTDSDMVAPGAVLTFRTSTVRRFGRGLLHVTGSVSCETTAKVTVVIGTVEFEAEEVVGNPVADFLARKGTPLVDTRALPNGGYSLTPSKAMLSSTSAAP<br>KSNHTYARISGDLNPIHVSPYFAALAELPDTIVHGMWTSAAVRTFVVQYAAGNQPGRVRRYQVSFTGMVLPGDKLDTTLQHVAMRNGRAVIRVTTSNGSGAVVLEGTAEVE<br>QNPTAYMFTGQGSQMPRMGMDLYETSAAARAVWDQADAHFQSTYGIRILDIVRDNPPSRTVHFGGQAGNRVRANYMRLMYKEVKPDGTSTLKRLFPAITEDTQFFTFHH<br>EEGLLFATQFTQPALTLMELAAYRQLLEKGLVREGSPFAGHSLGEYAALASVGDVLTIESLVDVTFYRGMTMQNAVPRDALGRSDYGMCAVNPQRVGPGFNQQALQYAVDT<br>IASQGGGLLEIVNYNVWQWQYVVTGELLNLDTLSLVLDTIHVKKLDLGEILRQQSLQSVIAQLERIVAECRAQAVARRAANGGRVAVQRGVATIPLPGIDVPFHSSLLLPGVAPF<br>RECLMEHLSPTRINVHRLIRRYVPNLTAIPFSLDRGYFEATLARTNSPKIKAVLKAWNPDPSPAEQQQLGFTLLVELLAYQFASPVLWIDTQDVLFREFNATRLVEIGPGPTLVGM<br>AQRTLALKYQKADQARNIKRDVLHIVKDRSVVAYELEDKVAASEPAAGPSAPTPAPTPAPAPVVVAAPAPAVSAPAASVPDAPMTPEEVVHVMVATKLKRKLGEVPLSSTIKD<br>LAAGKSTLQNEILGDLGKEFPAYTPGEKLEESDLRAVAGAVRPAFQNKPGPYTTGLLSKLVSAKMPAGFGLSRMKDYLSASYGLGPDRTSAVLLHALPLEPAARLGSEAEAQGW<br>LDGVVAAYAGYAGVALAKGGASPAPTAAFAAAPAATAAAPAAAVPDRPPTAADFVRVLVALKTKQSPKAVAPTRSIKDFAAGKSTLQNEILGDLQSEVGAVSVGEPAEVSLAE<br>LGQGIPGYKGSGAFSRSQIARMVSSKMPAGFGLSAVRDHLASQGFQEGSIAAILLHALALEPPARLGTVAEAHGWLRSVVQDYANLHGVSLSAAPAGAVVAVGPAVDHSGLT<br>KRLDGLFGEQLELYARYLQRDLREGDKRAETQAQQAAALQAQVDLWVREHGSVYAEGISPAFHAKKVRHYDSAWNWAKQDLIELVLRISADPAAGMDPTIVSTSALILNRSR<br>EAILHYITYETQRTMAKGGREFEVLKQFGYTLSATLPQFAPMWGLDWQTALATLSAPPRFVHRQPLTAPSTTVTPEGRIVYQEVARPGVATFEDYVQEVSRGFDPQNKSADQ<br>VVAECLRQLRRRPSAKQRRALLQQLASLNVKVNSKGPRGVPVGGRRGPHKESPPLVHVQTRSKQNPNAWAYDKALTTAYFQELTAAATTGVCFQLKTVLITGCGRNSIALEVL<br>QAVLAGGGRAIVTTSSYSKAQCDFYQAVYQQYGARGSALTVAPFNGGSVQDVEALVAYIYDQPGWDVDYVVPFAAIPEQGIEMPDVGSRSELAHRLMLTNVLRLLGAIRAQK<br>HRRSITTRPAQVLLPLSPNHGLFGGDGLYAESKIGLEPLFHKWHSEGWATYLSLVGAVIGWTRGTGLMAGNNLVAEGMEALGCRTFSTVEMGFLLVGLMTQRLATLSQTQPL<br>WADLTGGMDRIADLKGHTTRLRAEIQEQAATRSAVLQEMAREDEVVHGKPPAATPTIQRRANLQLTFPTLKSFEELQPLRYMQGMVDLDQVVVISGFAEVGPWGNTRTRW<br>EMEREGRFSLEGCVEMAWLMGLIVHFNGSLPSGEAYSGWVDAATKAPIADHEVKARYESRILEHSGIRLIEPELFNGYDPNRKLFLQQVSIDQDLKPLELSQEEALAFQRQHGD<br>ACEVWAKGDGQWLARLRKGAMIYVPKALQFDRLVAGQMPTGWDAKRYGLPPSIVDQVDPVTLFTLVSTAEALISSGITDPYEFYEYVHVTEVGNTSGSGFGGMVASQGMY<br>TNRLLEKPIQKDILQEHFINTMPAWVNMLLLSSSGPIKTVVGACATAAESVAVGVETIQTGKAKIVVVGGVDDFQEEGSQEFANMGATSNSVDELAHGRTPAEMSRPATTTR<br>AGFMEAQGAGMQILTTAALAFRMGLPIYGIVAFTNTATDREGRSIPAPGQGILTSAREKQLVPGTSPAPELAVAFRRKQLDRVRRHIKRWADEEAEGLQQEVAEAKAADPAL<br>DEAQYVTERLATLQRGLQRQEAAALATWGNHFYVGNPAIAPLRGALAVWGLTADDVGVASFHGTSTQANDLNESEVVNVQMRHLGRTKGNLLPVVMQKYLTGHSKGAA<br>AAWMLNGVLQYMLDGAVPGNRNLDNVDSRLEAFEYLVFPNRTLKGLQIKCGLLKSFGFGQVGGEVLVVHPDYVLATLSLPEYERYATLRARREAAYCKATHDSLTGTQPLLRV<br>KTEAPYTPKQMQAVYLDPTARAKYDPEKGSWSFARYGEAKAAEHPQEVQATQEALRQVVAPALAEAKGVGCDVQLTADLNLANDTFVQRNFTDAEIAYCRAQPDPQASFA<br>GRWSAKEAVVKAVSNFRPDAPAVWQGAGAPLRAIEVLPSPSHSPRVQLTGEALVAAEQLGVREVRVSISHSGAYAIAVAAAL
78	MPMMGQPFLYSPAPQQMGAEEAMEDRAKKWRLMNSKRYAKKRKFGYVDAQKEDMPPEHVRKIVRDHGDMTNKKFRHDKRVYLGALKYVPHAVLKLLENMPMPWEQ<br>VRNCRALYHITGAITFVNEIARVVEPIYIAQWATMWIMMRREKRDRRHFRRMRFPPFDDEEPPLDYGDNLLDIEPLEPIQMELNDEEDDAVFNWFYDHRPTVEGQKLDGRK<br>WRLSLSIMANLYRLAGVLLSDLMDKNYFYLFDLKSFFTAKALNMAIPGGPKYEPLYRDMDAGDEDWNEFNDINKIIIRHQIRTEYQIAFPYLYNSRPRSVHLQYYHYPALCYIKTD<br>DPDLPAFYFDPIVNPTPAYRDQPANAFPDYTPPEALLEEEDDFELPEDFQPFCSEFDLYDDNTAQGIALYWAPRPFNLRSGRTRRAIDIPLVKTWFQEHCPRSYPVKVRVSYQKL<br>LKCWVLNELHRRPPKALTKKYLFPTLRQTKFFQTTTLDWVEAGLQICRQGHNMLNLLIHRKNLNYLHLDYNFNLKPVKTLTTKERKKSRFGNAFHLCREILRMTKLVVDSHVQY<br>RLGNVDAFQLADGLQYIFNHIGQLTGMYRYKYRLMRQIRMCKDLKHLIYYRFNTGPVGKGPGCGFWAPSWRVWLFFLRGIVPLLERWLGNLLARQFEGRKSKGVAKTVTKQ<br>RMESHYDLELRAAVMQDILDMMHEGVKANKSRTILNHLAEAWRCWKANIPWKVPGMPVPVENMIIRYVKHKADWWTNVAHYNRERIRRGATVDKVVCKKNLGRLTRLY<br>LKAEHERQHNYLKDGPYITAEEAVAIYTTTVHWLESRKFSPIPFPPLSYKHDTKLLILALERLKEAYSVKSRLNQSQREELGLIEQAYDNPHEALSRIKRHLLTQRTFKEIKIEFMDLFS<br>YLIPVYQIEPLEKITDAYLDQYLWYEADKRHLFPNWVKPADSEPPPLLVYKWCQGINNLTDIWNTSNGECVVMVETKLDKVYEKLDFALLTRLLRLIVDHNIADYMTSKNNVVL<br>NYKDMNHTNSHGLIRGLQFASFIHQYYGLVLDLLILGLTRASEIAGPPQAPNDFLCFRDVNVEIKHPIRLYHRYIDRIHMLLKFTHEESKDLIHRFLTEHPDPNNENVVGYNNKKC<br>WPRDQRMRLMKHDVNLGRGVFWDIKNRLPRSVTTVQWEDSFVSVYSKDNPNLLFAMCGFEVRILPKIRSFNEEFVAKDGVWNLQNETSKERTAQAFLRVDDDSLKKFENR<br>VRQVLMSSGATTFTKIVNKWNTVLIGLMTYYREATVHTQELLDLLVKCENKIQTRIKIGLNSKMPSRFPPVVFYTPKELGGLGMLSMGHVLIPQSDLKYSKQTEMGVEFFRAG<br>MSHDEDQLIPNLYRYIQPWEAEFIDSQRVWAEYALKRQEANQQNRRLTLEDLEDSWDRGIPRINTLFQKDRHTLAYDKGWRARTDFKQYQMTRPNPFWWTNQRHDGKL<br>WNLSNYRTDMIQALGGVEGILEHTLFKGTYFPTWEGLFWEKASGFEESMKYKKLTNAQRSGLNQIPNRRFTLWWSPTINRANVYVGFQVQLDLTGIFMHGKLPTLKISLIQIFR<br>AHLWQKVHESIVMDLCQVFDQELDSLEIETVQKETIHPRKSYKMNSSCADILLFASYKWQVSTPSLLADTKDNYEGTSSTKYWVDVQLRWGDYDSHDIERYTRAKFLDYTTDNI<br>SIYPSPTGLMLGVDLAYNLHSAYGHWFPGMKPLVGQAMAKIMKANPALYVLRERIRKGLQLYSSEPTEPYLSSQNYGELFSNQIIWFVDDTNVYRVTIHKTFEGNLTTKPINGA<br>LFVFNPRTGQLFLKVIHTSVWAGQKRLSQLAKWKTAEEVAALIRSLPVEEQPKQIIVMRKGLLDPLEVHLLDFPNIVIKGSELQLPFQACLKIEKLGDLILKATEPQMVLFNLYDD<br>WLKSISSYTAFSRLILILRSLHVNFERTKQILKPDKNTVTLPHHIWPTLTDDEWIKVEVALKDLIMADYGKKNNVNVASLTQSEIRDIILGMEIQAPSAQRQMIADIEKQAKDQSQL<br>TAVTAKTVNKHGEEMLITTTSPYEQNPYSSKTDWRVRAISATNLYLRTNHIYVGSDDVKDSGYTYVLPKNVLKKFITIADLRTQIAGYMYGITPADSPMVKEVRCIVMPPQWGS<br>HQAVQLPHHIPDHDFLKDMEPLGWIHTQPNELPQLSPMDVITHTQLLENKLWSLDACIIITCSFTPGSCSLTAYKLSETGLEWGNQHKDNPTNPQGYMPSHYEKVQMLLSDR<br>FMGFFMVPDENSWNYNFQAGSHSQNMKYELTLANPKPFYHELHRPSHFLNFSTVEENAPVEVDREDFFH
97	MAAAPFTTLSMQRALGLQRAPLGAQRASSTSIDEMRFTSVEEFVKAMGGSKVIKKVLLANNGMAATKGIRSIQQWAYETFKDSRAIKFVVMATPEDVTANVEYIKVADECVE<br>VPGGPNRNNYANVDLIIDIAQRTKCDAVWVGWGHASENPLIPERLAKTPIVFLGPPASAMRALGDKISSTVVAQSAQVPCIKWSGSHCVLPPGTVNVDEQLYSQCCVKDLEH<br>CRQVAEKVGFPLMIKASEGGGGKGIRMVKSMAGVEDAYLQCCAEIPGSPIFLMQLATGCRHLEVQLLGDRLGNVTSLKTRDCSVQRRHQKIIEEGPVLGTKDGVLRSMEQAA<br>VRLAKLVGYEGVGTVEYLYEKVSGNFYFLELNPRLQVEHPVTEMITNINIPASMLMVGMGIPLQRMPDVRRLYGLDPTGDDLFDLDQEQMPAVCHTIAVRVTAENPDEGFRP<br>TSGKIYSIDFKPSRTTWGYFSIAAPGGVHEFADSQFGHIFSSGESREIARKGMVVALSQLRIEGEIRTTFEYVMNLLERPEFTNCDVSTSWLDGLLASKEPISQPIPHVTVVCGAIHK<br>MNARQEKNHMEYLAFLQSGHAPSNELLSQAFTEVLILNNMKYTVKGFKLSPTTWELVLNEGHLRVQTRILTDRGVLVYLGRESYLTTAENDPNGLRVVVNGKTAQFTNDEDP<br>CSLRTTVPGKLVRYLVSDGDFIESGMAYAEIEVMKMILQLKTNISGTVTLKAVAGTAVNAGKILAQIEPLDGTQIIRPDEYTQAWPADALQSRYVLPDALSDMREAVTGLHNILA<br>GYKYPPKVLEAEVRQHVEKLCRGCDAPLQGLGLAFLNVTPEGDADHPSRLVFLIRFFLEMFWSVEAPFDLQHEDVAVLALCKRHAGDLPRVAEVMKAHCSPDKGVVALALAA<br>LAEAKGLVPPIKEVLEKIATLSNVSNNRVVLQAKHLLRRMHLPSFNEMVADLHSMFEKGVDIQEMLYQSRFTSRMIISLMRDASQAVAKQALDLLVRRNYSAFELQDMEITGS<br>DDTMRARWSHALGQSVAAAAVAQNLAAFSDMGIGDEGAAAFMAKREWGLMFVFRTVDMLLERFERDVMLYMMEDEELRTHPSVVEILVGGHVGNALDERLEKLMADK<br>ANAFTLNTGCQRITFRLGIHSFFTFRRVSQFKEDIMFRDVAPTLAVFLELSRLSNYVIETYPLRGRGLRMYFAKEKMGMRMLPLPQREQDRRFFLRMLAIPDREEGHQFSATTLE<br>YIISEAIQILEVAMSDSKYSSVSNHLFVNFIEQHVNVDELDALLGCLYRYLKQLHKLYLTDIELKITTSDSQRIRIFVHNPSGYVPHTEVYYERMKNWKLYLEPAESLGGLSSSPSKTLR<br>PLRHCPSDPHPLLTAVDRKRLAAQKIGTTYVYDWAETFSVCVKEMWAKAVQARPGLAGFKEAMPQAPVVATEFVLEGGVLVPKPVSDCNNNCGMVCWEFDFFLPTRYRPE<br>TQTVPPKKVMVVANDISYQSGSFAIEEDDVFNAAGQRAKEKGLPLVYLASNSGARIGMAQEVKDVYRVMFADPEHPEKGFEYLYVEEADYQRLSTMKSINATPAVCPHSGRTI<br>YRLKDVIGVKDGLGVENLSGSGLIAGQMSSCYATVPTLSLASGRSVGIGAYLNRLGRRVVQVVGSPMILTGAAALNKLLGKEVYKSNNQLGGCPVMSPNGVTHWEVADDYA<br>GCDSIVQWLDFMPEQFPGAAAPDVPVPGPHFQLVPDPWDRDVLYSPTLGEVADPRFLITGLRAADGRLVPGLFDADSWQETLSKWATTVVAGRAKLGGLPVGIIAVETRTQ<br>EKILPADPADVTSVATVRSQAGNVWFPDSARKTADCIQDFQKEGLPCFILANWRGFSGGQRDMFDEVLKFGASIVDNLREYDQPVFVYLPPHGDLRGGAWVVIDYHINPDCI

	EMYADETSHAGVLEPSGLIEVKYRDSEIVQSMHRLDPVCKQLLEQQRASKQSMSADIQQRLRAREELLAPIYKSIATELAALHDTPGRMLAKGAIQGILDWRNSRRFFYQRLRR<br>RQWEFSVLRRLRRVAPGVTLKAALQRLQEWCGGVQDDAALAQWCEEHAAVIEERVQTVWKEMKAGQLMAECRANPLGQRALLLQLLQQYRESGADVAELRAVLAAP
**145**	MDPFSVPPPFLPPHMHPHAHLFAAYPPPFPPHFQPPHPPWAAPYPPYPTGDYDAEADTALDHPEEEGPAPPISPEAEAGPLANGPTPATDRVDGLPAAPPLSAVHPPLPIALP<br>AAPAAEAAEPNSDPAPGDRPPYGEEGEALHSPTGAASPYGPPPAPFFPPPPAPGHPSGLLPVIHHHEATIFSLRSELGVVAQQLGAAQAHIQLLQQREMAMAFQQPAHRTSH<br>TALTPVLLPPLLPTPAVRSPAPSHPTLDRFTEYISRSAMEAGIQSGSLLVGKLSVAKYSDQLAHVANPAFMGEVQVVGLYHRNRALPGDLVVVRLLPQDQWAPLPGAAPARRR<br>HTPRRTPVGAPLDAAATEAAKEAFGPDVDPLALYTPPDFDAAMVACGTVVAVLQARDKQELYCRLHPAKAALLDVTEDTDGEGVLPGPWKESPAATVEQLGAVVVSGRQRL<br>VRLTPVDPEFPCVEVPAEALPNPVPGDALFLVQLCPEAWPAAAAFPQGTVLEARPGALAAEYAALIHEAGLQATAGDAAGQVEGPAPGTADWAKRADLRSTDLTIAISDVPA<br>GVEGIDGCVSCRVITVGGQQAYRLGVHIPDVAHALARGTAADEEAAQRAVALQLPDCAVPLLPPAVLQACAVGSSGDTLAVSAVWTISAEGAVLNEWLGPSVVRPWGTLPA<br>PAVSKLLKGTAGPEDVVLRVEEAAREQVFASVTESVRQMYTVASALRRHRLRLGQLTVRGRNVGFKFGGSADAKVPVKVVPDTEHDVEGLFLVEELALFTQRRVALKLQQHLP<br>RHAVGAQRPAPADWKTAALCAAAKAKGVDVDGARPGALGWLVEAVEGSKSPTAVGIMAVVNHLLGPVQYSIAGGSHDAPDGPPATPCTCPTRRYLDIVVQRLLLHAINLEN<br>HRSEVPAAPATMLLAEDGEQRAAGYAPADLAQLCRQGTAAAAKAQRVQQAAEETCLALHLRALTKRDPWLTAVGYVVDVAPGALIVTVPAYGVTAEVAYTDRHQRWQEV<br>RYVKETHSAVVTWEGGATLELALLTPLEVRLWPARSGRSLRPAVHLLVWDHLDETQKAKRRANPETARDFVLVGELQERQQDFEAALASYTLALEREKPPVEAFLKRGDLLAF<br>HLRDSDAAMQDYDAAVAVDNKDPAPFLHRAKLLCNLRRYADAIADCMRAIWLAPTDVEGYLQRAAAFTSLGDADRATADYNTAIQLDPRAVPAFLQRGCLHAAKAGDYTA<br>AVTAVADFTAVIELQPLHALAHRKRGELLYERFHDVPRAVRDLVQVAALEPKQLHPALYQSYQHMGFATEAQYHDQHLHADQLEERNALRRRVGEALAGVQGCQLFVAGPT<br>LLGLGSAADPLALCCVPGYEAYADWWKSQGLGHTPHGYQAQYVARIAQALTERLGLAVEATVVGRCPTAVVKATEEAPIGLTVCLEYSTVRLSLLLERYLRPTWAKVVAFYVLQ<br>WARRQGDDAELFGTPATVLQLLAYFLLRKELVPFADPGHFPATQPVPADALFEACLHDELEHGQLMPTGEEGFRIGKVISDFFHFYTFEFDYASHVVALGTPALRCKKEKGWA<br>DPLCLEDPFAPDRNLTKGVSRQALTALRGCFAAAFDYVVEIYASTCGFDFYQAPAEDQAWPEATEDSPVDANEVQTAPKPMANGDASTEAEAEGHPPGHAPTANGFVNSSH<br>AASLPVSPPLAPAPPPPLKKKAEPTAEEFEALQALVKAVPARCRVTLKALETTHWKAEQRRKFGPLAAFVARFPAALTTEGALVFRTPPPSS
**158**	MTITPPEQQVKRVRVAFVSNPVETSFEKWSRPGHFSRLLSKGPNTTTWIWNLHADAHDFDNHTTDLEDISRKVFSAHFGQLAIIEIWLSGMFFHGARFSNYEAWLLDPIHVKP<br>SAQIVWPIVGQEILNGDVGGNFQGIQITSGLFQLWRSCGITSEFQLYITALTGLIFSAVLFFAGWFHYHKAAPKLEWFQNVESMLNHHLSGLLGLGCLSWAGHQIHVSLPINKLL<br>DSGVNPAELPLPHDFILDKSLISQLYPSFSKGLAPFFTFHWSEYSDFLTFRGGLNNVTGGLWLTDVAHHHLALAVLFILAGHMYKTNWKIGHDIKGLLESHTGPFTGQGHKGLYE<br>IFTNSWHAQLSLNLAMMGSLSIIVAQHMYSMPPYPYIAIDYGTELSLFTHHYWIGGFCIVGAAAHAAIFMVRDYDPALNFNNLLDRVLLHRDAIISHLNWVCIFLGLHSFGLYIH<br>NDTLSALGRPQDMFSDSAIQLQPVFAQWIQNTHYLAPTLTAFNLVSPTTPVWGGDVVSISGKVAMMPIKLGTADFLVHHIHAFTIHVTVLILLKGVLFSRSSRLIPDKASLGFRF<br>PCDGPGRGGTCQVSAWDHVFLGLFWMYNSISVAIFHFSWKMQSDVWGTVLANKVSHITGGNFSQGSLTINGWLRDFLWAQSSQVIQSYGSPLSAYGLMFLGAHFVWAFS<br>LMFLFSGRGYWQELIESIVWAHNKLKVAPNIQPRALSITQGRAVGVAHYLLGGIATTWSFFLARIISVG*
**181**	MASPIVTSQVYNLTTVDGGINPASINFKSVTMESDKFICVRDQTGDQNSIVIVDLAQKSNARHKIAAESAIMNPISKVLALKAGSTLQIFNLDLKAKMKSYAMPEPVIYWRWISA<br>QTIALVTATAVYHWSVDGQSDPKKMFDRKEELNETQILSYKADEHESWLLLIGVKKQADGTLQGLMQLYSVDKNASRVIEGHAACFINFAPPGQPQCCLMAVASNSAAGGK<br>MFVMEVPTASKAATTFERKVASVGFSSPSDFPVSLTASKKYGLLYMVTRSGYLYLFDLPTVTLLHSEAVTQDPIFATADEERTSGLLGVNTRGLVLSIAVNPQTVVKYVQQTCGN<br>YDVALRIAASGNLGGADDLFMAKFNNYLNEMNIQEAVKLVAEAPHGLLRTRETIQRFQAIPQLPGQQPALSVYFKHLLDHGKLNTFEAVELARIVLQKAGGAQYIQKLIGEEKLE<br>ESEELGDVLQPHDADLALKIYFKGGAHLKVIHTLVQRGDFAKIMAYCKKVNYTPDWPHLLSGVLNLNPDGAVQLAVSLNELDPPPVLPNQVVEMFVSRNMIKQVTAYLLDVLK<br>NDKPEEANLQTRLLEINLMYSPPQVAEGIFQNVSSGNLHLSHYDAMKIAQLCEKAQLYQRALENYIKVAQTTDANAINHVKRVIINTHAINPEWLIEFFGELSKEDSLDCLRELLT<br>HDVRRNFKVCVQVAQKYSDELGPTHLIEIFLSFKSYEALYYYLGSIVNFSKEPEVHYRYIEAAARVGQWSEVERVTRESDVYEPERTKDFLKEAKLSDLWPLINVCDKHDYIEELVK<br>YLHDNRQTKYIDMYIQKRNPMKTPAVIGALLDCDTPEDYCQNLIMSVGPMCPVEALVTNVEKRNRLKMLQPWLEARSAEGSKDQVLYNALGKIYIDSNQNAELFLKTNELYD<br>HLTLGRYCENRDPVLAVVAYERGQCDDDLVRVTSKNGMYKQQARYLVRRQDAELWAKVLAEDSDHRRQLVDQVVQTALPETTVPEEVSTTVKAFMTADMPEELTELLEKIV<br>LHGNNEFKNNRYLQNLLILTAIKADKTRVMEYINRLDNYDAQDIAKIAEGAELYEEAHAIYKKFGDNVQAVRVLLDDINSIERAYEFSERVAEPAVWSVLAAAQLKAEMIPEAVD<br>SYLKAKDATNFQQVIAAAEEGGFYQDLVRYLVMARQQSKVKEGSIDTELVYSYAKTNRLAELEEFISTPNVAQIQYIADRCFNEEMYEAAKILFTSISNYARLACTLVRLQSFYAAV<br>DAAQKANSIKTWKEVNMACIDAGEFRLAQMCALHIIVHADELEELVRFYEQRGLFDELITLMKAGLGHERAHMGMFTELGILYAKYKPDKLMEHIKLFHKRINAHKLIRTCEQF<br>HHWAEARFLYRHNDEFDNAIKVMMRHTAEAWEHEIFKDILTKVKGTELCYQAIHFYIQISPSQLADLLATMSSRIDNERVVQELRKAKQLPLGKTYLETVQDANLKQVNEALN<br>ELYIEEEDFESLRLSIDNFDNFDQLALAEKLRKHDLLEFRRIAAALYKKNKKWKQSVELSKKDKMYKDAMETVAESEDKDLTEELLRFFVDNGLSECFAACLYTCYDLVRADVALEL<br>AWRHKIFDFVMPYLIQTFKENLEIVQDLQQKMEKREEAHQPGGHGPEVMGLGSALVPVGMGMPGMPGMPGMPGMPGMMPGPMGGMGMGAAPMGMGSPMGYG<br>QQW
**224**	MLRRCEQCVQIKNRELEFTRNPWTCDACLGRGFGAQYPPAPAFGTWPPMAPQTLGTTLPINSFQNGVPAAVSPIVQLPTSTQHSLNGFAAAVPPQVPTPPQPVPQLNGFVP<br>TPVIAQTPAPAAAQPAEAVKTQPVAEVKATSPPKSSFSMGPKAKLVLEDGTELEGFSFGAEHGTAGEVVFNTGMVGYPESLTDPSYRGQILVITYPLVGNYGVPNGDLDEYGLP<br>RRFESDQIHVAGLIIATYSQEHSHWDASRSLGEWLKEHNVPALSGLDTRMLTKKIREKGALLGKIQFPGENIPFVDPNETNLVAEVSCPRVRTFAPLDASKAATAPRILAIDCGM<br>KYNIMRLLVSHDVHLKVVPFDYDIFSEEWDGLFISNGPGDPSMCSVTVANIRKAINLPQPKPIFGICLGNQLLALAAGAKTYKLKYGNRGMNQPCIDLRNGKCSITPQNHGFAV<br>DSTTLPPDWKELFVNANDYTNEGIIHNHKPFFSAQFHPEATAGPNDTEYLLKSFIDTVNGLPTKVHTMRIPPPMTGIRKVLILGSGGLSIGQAGEFDYSGSQAIKAMKEEQIETILI<br>NPNIATVQTSKGMADKVYFVPVQVPFVEEVIKRERPEGILLSFGGQTGLNCGLALEREGILSKYNVSVLGTPTRAIEITEDRDLFAQELLSIGEKVAPSEAATNLKDALAAASRIGYP<br>VIVRAAFALGGLGSGFAHDDESMTELCTRSFAICEQVLVEKSLKGWKEIEYEVVRDWKDNCITVCNMENFDPLGIHTGDSIVIAPSQTLTNSEYFLLRRAAIKIIRHLGVVGECNI<br>QYALNPESEEYYVIEVNARLSRSSALASKATGYPLAYVAAKLALLKDLTSLRNSVTKVTTACFEPSLDYCVVKIPCWDLKKFTKVNPEIGSAMKSVGEVMAIGRRFEAALQKAVR<br>MTHPSLQGLMDGKKMFIDLEAEGTEEEKYELFKSAVMKELVKPSDKRVFAVWEAFEMGMTVSDVHNITNIDKWFLQRIHMMHACSVEMRTIKLEDFNLDFLREAKALGFSD<br>TAIARYVGSTEPVVREHRKKMGVLPVVKQIDTLAAEFPAMTNYLYLSYGGTENDVTFDQPGYIVLGCGAYRIGSSVEFDWCAVSCVETLRSEGQKAIVVNYNPETVSTDFDKSD<br>RLYFEELSLERVLDIYEYENPKGIFVSVGGQIPNNLAMPLYARGVRIMGTSPLDIDRAEDRFKFSQLLDVLKVDQPAWKELSSLEEAESFCRKVGYPCLVRPSYVLSGAAMNVVTA<br>EADLKVFLGEAAMVSQEHPVVISKFILGAKEIEFDAVARNGEVINYAISEHVENAGVHSGDATLVLPAQKLYVETLKRVKKAARLIARALNICGPFNIQFMAKGNDILVIECNLRA<br>SRTFPFISKTLNIDFITIATKAMIGMTIKPVVMNPYDLEYVAIKAPIFSFTRLMGADPVLGVEMASTGEVACFGEDMYEAYLKAMISTGFKIPKKTIYFSVGPLASKLEIMEAVEIFA<br>RLGFTLFGSEGTCEFVRERGIAIQPEPKIVQVLKEKKMDLVINIPSPGDATTEDTQGYLVRRAAVDFGCSLVTNVKCANLLAQSLERVTKEKLLESRPWSEYLQMTHFFDRS
**256**	LSTAKVKVKVPFLMNPAYTLREYQLIGLDWLNTMYHKGLNGILADEMGLGKTVMTIAMLANLACTKECWGPHLIVVPTSVLLNWEIEFKRFCPSFKVLTYHGSRQYRNSLRCG<br>WSAENRFHVCITSYNLILSDAPMFRRKKWVYMILDEAQYIKNWKSKKWETLLNFNTERRLLLTGTPLQNNLMEMWSLLHFLMPDAVAFESNLEFRAWFSNPMQEMVTGQS<br>TLNEHLIHRLHSLLRPFILRRLKKDVEKQLPKKYHHVLSCKLSKRQRVLYDDFMSLATTKDAISEGSYTGLIAILMSLRKVCNHPDLFEERPITSPFDQLEPIDLRVPRLACKPMEHM<br>DDRLRMLRWLHTELLDTWSVSAIAERCERPTVISEAMRLPPEEDVAQLHPTHLFGPYSTALCARRAKLQEERATLIAHMNAVRCTIHNLGITGNLVRGCQRLADLRSSPRLALL<br>HASDCFEPCGSTLMRTLVTTSEERMGNHLELLKKFTCFIAAARARPLTISAYLPIHYRPQKFRALRMEDVPHFDLRTLAPYRPTFVRLQMSFPDKYLLQYDCGKLQVLSRLLAKLK<br>AEGSRVLIFTQMSKMLDVLETFLCMGGHTYVRLDGSTKLEDRQFLMERFNHDDRIFVFILSTRSGGLGINLTGANCVVFYDSDWNPAMDLQAQDRCHRIGQTRDVHIYRLISE<br>HTIEENIYKKVQEKKMMVNLTLKGGQFGADFVANKVNVRDFFHSFDEDMDQDEIRKLQLLAGENVEEVDAPVPKIVEFEGAAEVLTEELPPLPSAMSLEVDKDVEVAMIKAE<br>DEDDRIAFKKAKEESSKELKEFDENFQFKDLDPVDAEVLEEEFMAKLRPLVRRSIEAYVDLYPDKAAEIAQEYRVEEYEALVADGNPGPLTTTSPTPTPASNPHPAAAGQPGEAP<br>ADAGSLPCGDGPGLSQPSALPRVNGSCPPEAAGGPATGKAGRPKRANDHPDPPAKRSRVEHPNELGEEPEGGGSSPLDDLPPPRFARTVVRSYPSGVHVLVKLPVPPPKVRT<br>GGTPCGLGRRPPVAEEAEEDDDEDDNEVALFYETEHADDVLAQYRGIQFDLDDADLEHFYLHCLQKDQDRLERERQLREYLRADASESDAASEASLATKEDPGAGLAESVPRL<br>ADAEEAVSSDPRPSRRPVRSTATSFTGFGPDTEDGLDQESSLDAIPRRRPQRRASRIDEPIIPVAPLRRGNQEECLQLLQEDNSELPWRDWEEAALLKAVETFAPLPAGQVLPTG<br>ASAVLPPPPRAASPAASSHGWSWDLLCDVVNAIGFPSATPRVAAQCKNRHAQIMTGHAPLPAGPPRQRPRLVDQPLGAIQKELKRRQLHHMAAVLAQPPGRAPKVLQKNR<br>RKGPEGYPFSDTEADGGTARWSPVPGPGPPGPLPTGCPELRGHTPKDLLRVIAEKQRQPPEVAVVPQPSYLSRQPKGSPIPGSSAFPPQAAPYGFTAAPPGPRVVPPLHAVGG<br>ALSVMSQPPTSPLPNGDPLPLDGGPKGSAVPAAWELPPLGAMQVGPFPVQAAGSDPSTMLAALPSGHHLPPLSFPGFPPPTMSVSPSATNTGPTGSTVHQPPHTTALLQPP<br>HHHMPTLFLTNDHTARLSSNPSHLSAHAPLGPMALGSGPLAVHNGFAALQGSMGGLAPPPFPQFQQQLSSTPHPMMALATMHPRHLHLQQQFQQQRLTMATQQLQPP

**595**	MEMDFGPPPRLPPFRRGTRIQLNHSDMDDGWGFLGPSLLRARRNQSPRPWIGIRANAGASRGRVAFSFVNETGGNIRLGWSTVDATLELGMDWRSFGFGGTGTKSHGGQ<br>YIKWGQTFGKGDVVTSLLDLDKHSILYMVNDHLLPEDAFSIPKGLWGHAFFPHVLVKEATFEVYFGPGQGHPKTYIRPPQLPGGFSWLCDGADVLSPIVPQGPPPFMDETLAEI<br>EAAKALEKMMQCGAANQACHREQFADWKVKVNTYVRHFQAPLSFEYTAEKEQVMNRIAKQTKQQLMRDGYGCYQMTAEFLDEGRKKGADVLILVLRPTRGVVPRLAEIS<br>MGRVVLISAEDGEVDIERAEFTISGEVGEIKREGIIVSTEYSKLPYSPNRLYRVDLGPNVATFARIDLMLDTLQREEPAKPNTFGRMGIINPGTALANCLFADIDETATAAQIWDPS<br>ARPVPNPDADISIAARMRHDPPSRPWYRKGTVPEPRPLNSTQRRAMEMVLQEERKLVFIQGPPGTGKTTTAIEIICGWIGLGKGPILATAFSNKGVDNLAEGLWQRGVKVLRV<br>GHCDASAPYALDGMDRSQQQKSLKEADVIAATCIGVGMRLLRDIEFPFVVVDEAAQIIEPAVLIPISRGSRQVVMVGDQCQLPATVISAEAQKRGLDVSLFERMLTLGMEVH<br>MLGIQYRMHPLIAHFPSWRFYRAELKTGVLPEEREHNFGVLRRPLCFTNVDGRESSQGSSKLNLAEAQCVAAFVKALPIARSNIGIITPYAAQVSAIKHALSRVIGDEALETTVAS<br>VDSFQGSENEVIIVSMVRSNFSGSVGFVADWRRLNVALTRAKRLCVVVGSLPTLCQNALWRDWVGFHASLPQQTVDWCNWDAENGTLGPLEQNKEALAIAKDAQRQFAV<br>TKEAELKKALPLRGEYPGLAIEERQVRKQAAKVMVEVPKAEERVNLAAVEDQEGKVYHTADDILRKWLTERLNAMAKEISDAKGKGNWIEMMKVMAATGLSLKNSSAQVVE<br>RRVDPADGNAYTQEEFVECYGGTAEWDAATPVSAAPPAPVAVSAAAHPPQPVQPPPAAQRLAPPVATPTAPPPLAPTYPGASGAYAQPQVGYGAASHDPYAAAQQYGAE<br>VPQAMLPMGQPLPLQQDLQAQYLSPYDAQAQQMMQALQGGMGQLQFQQQSPAPYPDPYYSPPQPQAQYPPQPQYPVPQGAAQYQQPQYPPQPPAQQYAAVPGGV<br>PPQFAPQAMPPYQQPLPAYTHQQQQPLTQPYAPPRPQPASQSTALPGTPAAAPRQAVSLAVAAPIQPPPGAFPPGPPQWEPLATSHDAKPQRERKRRHG
**680**	MEPARASAPAPAAPAVVQSCPEFRRPASVRPKKCKSLRKATVRRELLDNPNEKVIVGVCAMAKKVNAKPMRAILGRLESAGDIRVERFGDKVILEDPIETWPRVDALIAFCSDG<br>FPYEKALKYVELRKPFLINDLQKQYLLFDRRLVYQTLVKNGVQVPKHIIVNRDTLMQGQDPEGFIERENYVELNGVRIEKPFVEKPCDADDHNINIYYPSSMGGGVKRLFRKVGD<br>KSAEYDPYHPGTVRRDGSYIYEQFVATGGTDIKVYTVGPRYAHAEARKSPVVDGKVIRTGDGKEMRFPVLLSPAEKEIARMVSLIFRQKVCGFDLLRCRSKSYVCDVNGWSFVK<br>NSGKYYADTAIILRNIVFSALSINKIFRQLNVSEAMRREAEEFKYEFGTGETNLDEPIEHSDRELRCVLAIVRHGDRTPKQKMKMIVTQPPLIALMEKHLDVKGKQAKLKAPSQLQ<br>ELLDITRRMLEETRRDMQAAETKEEQDALLETSEKLRYMRAVLEQGTFSGVNRKAQLKPIQADKKQKEKEREAARAEDAGSPLPPAPDGNPVPREGSELPGAAGLAAAADPP<br>AKPTEEGAPDAKPEKPEKPEKTDKADKAEKGEKKKREVLLVLKHGGVLTHSGRQQAEQLGKEFRTVLYPSNLRGGLLRLHSTYRHDLKIYSSDEGRVQSSAAAFTQGLLDLEGAS<br>LTPILVSLIKKDASMLETFGKGANADIQAAKALLYQQLTYDPAADQTFSIPVPLPERTEAFEFTDSEAVRKRSRCLANDDEDSVSETGKHTDANPSSEEFNKQMGSLLSVEDPTYG<br>MDPKPFKGGEARFHRMSDQTLAHCQEMVGLLKNLVQEIQARLRLEREVEKGTDKGTEKEAPVDGDVKKAEDQAYSALVNHPSEWDVKALNEFTPCMSEKLILIYDRWRKLL<br>KGFYNEKKSLFDCSKIPDVYDSVKYDMIHNQHLGFPLERLYAVSRKLANCVIPNEYGTDVQSKRRIGARICSRLLGKLLNDLDFMVDESVAASAGLDMTLSDDVDDTEPSHQNR<br>AHTLERQALEVAYSFEDEELDSGRSAGGDGEEEDEENDSDDEVTRLSPEYASGIEHPMRHVRTRLYFTSESHIHALVNVFRYAHVDRPELEEGPTLVGPEGLELLSQTPELDYLSN<br>IVLRLWEMKTCALDDPERFQVEAMFSPGALYDGKAENHCLPPVQHQPLHPTYFVNLKRMHQLLEPFSTPCKRPAAYWKVNAKPSGSFIHVSPHNRGPAPMMSPTS
**712**	ENFKSYRGTVTIGPFADFTAVVGPNGSGKSNVMDAISFVLGVRAAHLRGASLKDLIYSVEGEKTDRRASVTLLYRARDGREMQFKRLVRADGSGEYRIDDVGKTWEEYDEVLR<br>SIHILTKARNFLVFQGDVENVAQKSPKDLTALLEMVSGSDEFKEEYEAKRRRKEETTARMLDASSKRRGVGAEKHQYRLQKKEAERFRELRAEQTNAKRDFALFQLFYIETELTA<br>MRRELQRARRELQELQAGNQEGGREYEQCKKDFADLHKKSLALIKTKQDRSARQSQLQRQADKLVLQVRFTEDKLAEAQSQVKKVREAGKQHTKDVQALQRQLDDVKAEQ<br>AAWEKKRDAEVKALEGQALTESQYQEWRTLKQTATTKTITVAQEIGVLNRQKTAEALQLKSLTAQAEQLEGRRRQLAEAVAQIQERRDQLRAGLGKGDQEGAALRDELAELK<br>AKLGEREAKRAELDQALAAVAAELSELRFEKHESGRDKKFADTLEQMKGLFAGVKGKLHELCRIPDKRYQTAVTVAMGKTMDAVVVDNAACAMECIQFLKEQRLPPITFIPLE<br>TIKATRVDERLRRLGGTCRPVVDCLQFEDVVGPAIRYAVGDTLVCDKMVEAKRVAFGEVDGERHKVVTVDGSCILKNGCIQGGQATMERKARKWDEHKYEAQKRKRDELVR<br>ELDRLGDVTALRNRELDLTSRQAALQASRDSVQADIAAADRKLETSNKELDAISKQVKELQPRVKEAEKKVVAVANRMQELESKKNEMERQVFEDFGKRVKIGDVQAYEERRD<br>RLQAEAAAKDAELKELRARLENQLDFAERRAGTKTVKGAEKAVADLTAKLAAEKSEQASTAKELEKVTKEAEAVRAEAEDCKKAMAKTDGRLKELKRALGDKLDALGTAKKRV<br>TQRQGACQKLRSVRAATIQKCGVEEIALPMAPLPPKRKAAWEARRKRRKVDGGAAEGEEAEEEEEPMPEGGRLDYSEPFNLAEDVGSQRAAGGAAGDVVTIDFSDFDPKLK<br>AMASNAKEYQKQSALFDDRLQRLELDLEKLAPNLKAMQRLADVEDKFDGTTRQFEECREEHKKAQAEFDAVAEKRYSKFMATFDVISDEIDKVYKILTRDDEFPAGGTAYISLD<br>NPLEPYNGGTKYNAMPLTKRFREMEQLSGGEKTVAALALLFTVHRVRPSPFFILDEIDAALDHGNVVKVCNYVLSHCHTCQFLVISLKPEFFENASTLVGVYRDQKLKASRVLSW<br>DLTQYDRAGEAEGEEG
**791**	MASKQRSVSPSTAESNIAWQLCDKAFRGDIAGLKQFLDAGQSLDVQDYDGRTPLHLAASTGQFATVKFLVEEAKCTLQRDRFGGLPIHDAERSGHEELKEYLQNLDFDAKLVC<br>WHGNVDAAKQALMDKVLSLVLKQGVWVFGLAFSEMEYYFHDLGLDDWYFKHYTPAQIAKHIHCYIASKKVGQTTAEYTIQFHMEAPSTAFFLTSIGENKKPLQQTEELVTKW<br>MNAVPEDQAYSMTYVESRGAAYRGRKDRLAIFVVESNPFQSHEVDEEEDSLELLATTNFLKEKQPKHQVSYQRLIQQVMRSSTDSWAGVVPTTSLGEQATRWGQWLVQFGI<br>KQRLPVFLQQFQLALDRLGVQSRKKHIETFQNGVTCYHFYCDTLPGQQAEDCLRAFQLVPHMKQSPLTHLFLDNKICSDSLIYISCAVKFAFHFVKKDTREYLILANALQHDPAN<br>KEKLDQLYLQAIMDLLTEDRVYDVVLRYPDFCRQLYSDFRQIALGERERFLNDTLAKQLAMNRDETDVKVLSAMLTFNQHLTGTNFFRCGGTPSSLAFRLDPSFLKLVSQAIFPEI<br>PHTVYLVVGRSFYGFHIRFRDVARGGIRVIKSRDESMYRRNAGTLFEECYNLAFTQQRKNKDIPEGGAKGTILLNPDAQAKTKEAFTHYVDALLDCMLPDRAGIHCPHPDILFFG<br>PDENTADLMEVGASHAHSRGYPLWKSLTTGKPPALGGIPHDVYGMTTLGVHTFKLGLLQALGKREEEMTKVQTGGPDGDLGSNEIKISKDRTIAVVDGSGVAYDPAGLDRTE<br>LLSLAEHRLPISNFDPAKLKSGGFLVRVGDSNVTLPDGTLVQRGDLFRDSFHLSAYATADLFVPCGGRPKAVTGDNVQQMLVNGKPKFKCVVEGANLFFTDSARKALEDAGCH<br>VLKDASTNKGGVTSSSLEVLAALVMPDEDHQSLMCVPQGGRVPDFYLQYVQHIQQIIAEKARMEFSCLWAEKQRDPKTYYCATTERLSTKINGLSDMIGAEVVDDADVDLIRT<br>VLKRCIPALLIDRYGVDFIMKKLPPNYMHATVAAWLAATYVYECGFGATEYAFHKFLGKLQGDGMAHGVSSVTLDFASQ
**854**	AHQMECSSSGIPNATILTIWGRPDRPSTRNGSLVCRAVAEEARIDMDELEDDVLHDEEGILENSLPVEDEVSAPEKKPAGQQFKKQPSGKEKAKPAKSYTTPAVVRAREQFVRS<br>VLSRGVTYDDNLPICQAREEIADAIRSHQVVVICGETGSGKTTQLPKICLELGRGQAGLIGHTQPRRLAARAVASRIAEELRTKVGEGIGFQVRFTDESSDSTCCKLMTDGILLAEI<br>PNDRLLTNYDTIIVDEAHERSLNIDFLLGYLKEILPRRPDLKVIIMSATIDSERFAEHFNNAPVLTVEGRTYPVEVIYQPGKRESEDPISIRMAVETLIRREKETGAEPGDVLVFLPGER<br>EIQEATVYLQRFFRGFEILPLFARLSRSDQDRIFSPTGEQRRIVLATNVAETSLTVPGISYVVDTGSARISRYSLRSRVQGLRVEAISQASANQRKGRCGRLRPGICVRTYSEEDFLER<br>LAFTDPEILRTNLASVILQMLWMGLGGVAEFPFIDMPKRRAINDGMELLKQLGALDGSKQLTPLGVQLARLPLDPQLGAMLVAASKEGSLREMLVVVSAIAAQDPRNRPADK<br>REAAEAALAQFNHPKSDFMTYLNIWQAYQEEKSAGNSLWKWCDACFLSFTRMKEWQDLHGQLLDACQRLHLRLNSTPATYSSLHRALLVGLPDMVGVKDERNEQYAGCN<br>GKKFRIHPSSPMQKKGFKWVLAGELIETTQVYASNVAKLEPQWVESILPHLVRLSYAKPYWDSEANRVSVYKEVTLLGLEVMKKKISHHTVNRAEAREIFLRDALATDEYDSEAA<br>FLAHNRAVLAEVQAYETKLRRRSSVNASMLFDFYNGVVPQTVATPYEFEKWYADEAPSESILFLPDSILSDLRSKAPPDLYPDLWTLDGHRLPLTYRYLPGSNTDGVTLTVPVLAL<br>GSLKAEALEWLVPGLLPAKLEVMIRGLPNLLRGSWPPDTNQRVMKAVTGGEFGRGCLRTALTRAIEQAVGRAFLQTPQDLWQAVKVPPHHMMRIEAVDVNQRVVAEGRD<br>LGVLQRQCANAAYEELAPPFVRKGLTTWSFGTWPKPELCDVAGISVLAHPALVDCGTYVNLTAYADSTYAEQVTRMGLVRLALLSRPRVNAEVERQLKANAGTGLAALKTAG<br>APTSFSSSSASSAKKASAEAPRPAGQIVASVSSTADKDKARTGSPFQALQALRAKLVQQTYIPASVPKPQPTPRLQPSPLAPVSPRPTPAAVKPPKAAAKPTAPPAPLKINAAELA<br>ARMMAPKDPTYVPLP
**899**	MGLPWYRVHTVVLNDPGRLISVHLMHTALVSGWAGSMALYELAIFDPSDVALNPMWRQGMFVLPFMTRLGVTKSWGAWSVTGESFSDPGIWSYEGVAVAHIILSGLLFLA<br>AIWHWVYWDLDLFRDPASGELKLDLPRVFGVHLFLSGALCLAFGVFHVTGVFGPGIWVSDPYGLSGKIEPVIPSWGAEGFDPYNVGGIASHHIAAGLLGLIAGGFHVLVRPSQ<br>RLFVLLRMGNIETVLSSSIAAVFWSAFVVSGTMWYGSASTPIELFGPTRYQWDKGYFQEEIERRVQASLSDGCSLSEAWGAISPKLAFYDYIGNNPAKGGLFRSGPMNNGDGI<br>ATAWLGHAVFIDKEGNSLFVRRMPTFFETFPVILLDQNGVVRADIPFRRAESKYSIEQVGVTVRFFGGSFDTLSFNDPATVKRYARRAQLGEIFDFNRSILQSDGVFRSSPRGWF<br>TFGHLSFALIFFFGHIWHGARTLFKYLLAGIDPDLEEEIEFGTFEKLGDDTTKKELV*
**1288**	MGSPSCLQGKTFVITGQLESLDRQQAEDFIMRHGGTVRTTVSSKTNYLVVGEEAGSSKLERADKFHTSRLTEDGLIALVVEQSQRLGADPWAWKGGSAETGSDADADGEPM<br>AVDAAPGAPVPPPSVSSMPAPGAPSMASLLAPRPTAAAFYGADPSASSSSSAGVNRTQPASAYPTPSVAWEDQLWADKYRPLSLRSGMVAPPQAAELKRWLEAWPENSAK<br>RPPPPNFKKAALLSGSPGIGKTTTAHLVGRERGYEVVEWNASDKRSKRDIDETILDMVNNTSVKALFQVARSEGQKQKRCCIVMDEVDGCDRGGVGEVIQMIHRTKVPIICIC<br>NDRFHPKVKSLANHCLDLKFNKPNRTQVAAHLQRVLHKEGHDMPLPTLETMVTAYSNDIRSILNALQMWFRTRRNIGHDEARGLQASSNKDHEVGMFEAPELFLTPHTPKP<br>TIAQLREVYFTVDFVGLYIQENYVNMRPDQCRSNDERLALLARASDSISLGDISETLVRRDQHWGLSSVGVFQSAIYPSALVRGKFESLKPPSGWMDNRLRFPGWLGQNSSRK<br>KNDRMITCIAKAARNPLTGVTGSSAEVRMYYFDLFTRNITTPLATQGKDGIDAVTAFMDTYHLGRDDWEFLQELHQWKRLPVRQAPLPPIDSTVRAAFTRSFNKTHLMSAVK<br>AVAPTTVREENDDGLLEVEEEDAGSEEEDVAKDAMIVAKKKGKAAAATSAPPAAAGKAAKKAPRAQKRK
**1309**	MSFTKQYLSMRNSSGQSRADGLNRGLSYTFSRERTDGDGEMLDSIYDDDFLPEDAPSQRKGQWRTLHAVFLLTYAGRKMRPSGEFAPLAGHSANFRTEDGYVLKLLTQNEA<br>AVLESLQRERSLRGFAPQFMGCVERDGRTYIKMQDVLLSMAEPNFMDIKMGLRTFAECEVESDKRRMDLLKKALVIQQQNPAVYISDDEKLNGISKYRFMSLRDNASSSATLG<br>FRVQGMMKDGKGFTSKDFSSHRSATMCRALFRSFFERASQEQRQGLLKRLQDLHEVASMSDWFANHEVIGSSLLIGYDTASPSKYGLWMIDFARTEPSPRRLTHTRPWTLGS<br>GEDGYLLGVQALAKLWYDEFRGDIPLNAVDWHYRAEGHDCIVIGYHGLEPALQGKVLRLAKEPAIRRQRDTFTGHPATAYNDFVATMLTDYIGPAADPGQVVYVSPQFLQAI<br>AQQVEGIRPQFRALQAGLDLKSEYQLLLQDSCHIPGVGNAKTVAVELKLKMGYLPAPVDSIHWIKALASRFAMQQFLKLHQGSIEKISNYCPADLFSGDFDRIQNVLYRLLDTP<br>QNNLRVFYEDALLVPQQEGKASSVQSGKGVLDTRLEHLLGASGLPPSLYLCELLAKALIQEGPQGTPPLLSKILNLQRLGLDIEVLWPLVKDGVHELQQIYHVDPLDVNAVAQAL<br>SPPHPIHCQSVQQRERRGEYGCELANLQPLYFAARAGRRLEMEAVEERVRNFDAVTETRKFLLSQSLKDASLMVALAEVHEPGTLPLPNIVRGSDGRYWKFEMKLMDLDAKN<br>MQHLEKWYELDRTIVQTYMLHALGAGSIPEEDTPRGARPTATPPRRTSGVSPARSVSPAVRPPAAASP

**1453**	MAEAPVPVDPKLLEELCASIAVAANVVRTLKESEAPEDQLEPAINALMTLKVKLTDLVGVGGHEQALNKSAYKKEKKRLESVTEKPKVKQEFKQGEGRNSKLAKKMLEKRQTDLQNKTPHGQKKILDEEMAKEYDPEAVEMAWYAWWEESGYFKPRDADTTSEKFVMVIPPPNVTGSLHIGHALTCSIEDLVTRWHRMKGHNTLWVPGTDHAGIATQVVVEKQLYKDTKQTRQDLGRDDFLAKVWEWKTAKGDHIYNQLRLTGASVDWSREAFTMSDKLSRAVLEAFCRLHERGKIYRDTKLTDWCCTLQSVISKIEVDNVELEGETKLSVPGYDKKVDFGFIYSFAYPVLDSEEVIVVATTRPETMLGDTAVAVHPDDPRHKHLHGKFVKHPFVDRKIPIICDPILVDMNFGTGAVKITPAHDPNDFACGKRHGLEFIDIMTDTGEMAAGCGEFSGMKRFDCRVAVMKRLDEMGLGRGKAPNPMTIPLCSRTGDVLEPRIKPQWYCDCTEMAARAVAAVDAGELRIVPASHEAVWRHWLTNIQDWCISRQLWWGHRIPAYYIDFRGEPAGAKSDVTRWVVGRTLDEVKQRAAEKFGRPVEDLILSQDEDVLDTWFSSGLFPFSTMGWPDDTVDLQQYFPGSLLETGYDILFFWVARMVMLSLELTDRLPFRDVFLHNIVRDKYGQKMSKSKGNVIDPLWVVYGITLEELHANIASGNLPEKEVQSAQKAQREQFPEGIAPCGADGLRYALLLNCSLSSDINLDINGVVSCRNFCNKIWQATRLSTSNLVGFTPAAQPMEGCGERALVLRWLLSRLNHMLTRLDVAYTVYDFHEVAEAGRAFFWEEFCDVFLEYTKRIFQNPDHPQRAAACETLYTVLETTFRALHPTLPFLTEELWQRLPRRAGVTTPSIMLAEYPAPVAEWTDAQAEVQMELVLAIAKAMRATKVNYKMAPKATPRVTVRVRQAELAGLVRDHVEDFQKLSGVGEVEVAVGDSAPAGYVVTTVSEQVDVLMELAGNVDVAAEVAKLEKEKLKLEQTRDKLRAEQED
**1597**	MDPHASLLLSLTLDKDTSFVDPFRADLKLLQALLLSCLVEKAGIPPKVMDFQRLTKAFAKTKSPEDFQALVQHAESLTNEEALVTARLFLELIHLSNLAEKHHRLRRWRAYRRGEGAIVLHHTLEDCIKSLTDQHGIAPEAVRKQLSAQRIELVLTAHPTQAMRRSLLVKFAKILQLLEQKDSHFLTPSEELIVDEALRREVFGAWRTNTLRVLKPRPEDEARSGLSYIEDSLWNAVPKVHKAIDIALHQLDLPPLDEDTRLITFASWMGGDRDGNPFVTWQVTETICNLCRYRGADKFYDELEKLLFDLSMHRTCSAEMQEFIAHTEKQHAGDSQWDIWQNHQIAHDEPYRVVLQHLHVKLEATRNRFKALYEGGAVSPHILERPYFTTTKELYDPLKMLYRSLHEVGDGIVAEGLLKDTLVRLKTFGLELVKLDVRQESTRHAEAVDAITKYLDLGSFLEWTEEQRCDFLTRELTNKRPLIGADFEASADVVEVINTMKMIARVGPDSFGAYIISMARTASDVLCVSLLQKVGGIKHPLRVVPLFETKEDLISAPETVEALYRNHYYRSHIDDHQEVMLGYSDSAKDAGRITSVWELYKAQERLVKLSADYGVQLTLFHGRGGSVGRGGGPNYLAIQSQPAGTLDGGLRVTIQGEAIEQFFGISTIAEQSLERYCTAVLNTSLAMPPPPPVEFREVMERLSQTSCNTYRALIYENPLFVRYFHDITPASVLSGMNLGSRPAKRKAGGVEQLRAIPWVFAWTQNRVHLPVWLGVGTALRAEMEAGNKELLQRMYREWGFFQSTIDLISMVLAKADPQISRLYEEMLVEPGLWEFGQHIRDLLAFTTAAVMEVTQETFILMNQPVLKRAIDNRMPSLDPLNVLQAVLMKRDRAPDAEADTVLQDALKVTVQGIAAGMQFTG
**1731**	PAAPTPTAPSPFPTLDLTAAAAPPPPAPAPVDEAVRGEPAPPLPPAPVEPPVTTVVDVPAAASSSGEEGAASPSPWYDSEGNLSPLSLSATAVVLLLSAAAISFLQAEKPPGTAELETLVEVMQPLRANTRKLGTDAAIQKLLDTAEKATNSVVDAETFEKAIGPVVDAVEATLEAIEVQVSAVDEATVQAAKVAQAAADVVEKNAELVVAAAQSTDRAGAVAGRMADVTAAVELATQAVGDEVEIVETATQEVTKVARGAVKAIEKTARLAQDAARDAEVDPQAMKELVASVTSVVVDAADALRAQMEVVDLAAQEAARVAEDAAKTVAENAAAVTEASKASQREERAMVAATSAVSKAADAIGVQAQVAGTAAEETSKLAAAVATTIKQNVDLAATDPAALQEAVESVSGTVADAVEEIESKVQAVEAAAQQANKATEKAANAVQRNAERVAVASKALGGGPGVSQSMQSVSDAVNDAAQAVGDEVEAVGAAAQDAINAARAAADSITKKMELAAEATRDGSVGALQEAMRSVSDAVTEAVEAVESQVRMVDLAAQKASQVAQTAADSLEESSARVSATSKQVLNIGEVDEAMTVVRSSVSEAADTIKSQVQVVNTALQQLGKVAWATGWVLLSVAQLLQTAVTNLDPEVFKTVLQVLGYVMGIIIEALKVLEKVVVAEMPRVIAATNTVLAFYREQVRNVYAALPPDVQQQVISADVLAASPLLAYVGTNAVRAGWKRLLYGEGEWDVRPDQLRAMLLESDTLLVDIRIPKEALEGVPDLRRGARGKALLLSPLPLPPLVRAKASDPDKLMYELHAQTVSSMWSVRFRRAVVVMDRDGTKGQSFARLLRRKGIGQAYYLRGGFNRWKAAGLLVRPTYDVGFTDYVQQEAEALVLDALGLLGFSRPPRPAPEATAAKGSSLRIDPPPRPPTQGSRGPPKEGTLGRRTSPPPRSPTLGRKAELASPRGSSRVTSRR
**1761**	MPPPPKEPRSSPEQTSPHRLAHYNHVVRLPTQSARYTLANTRVEVERRAPCNPAIASEVRKDATGSRVGIALGSSSPSAAPVVFCISGPRVLLWRISGDGPLQQQVNVSAFILNDPSALEAAVVGQGRSLLLVHRLTCRAFFFRDVFRQPQAMSKLALATSANDEAVARFSQQGDHVVIVTTQQNVFVIGPKDFESTQPLTPFICRTGRSFWGLGALFSKPEDPIIHAQWSAEDECLVVVKGNVVEKWRAGAKVSSFALEGDLEFRALQVQSADVIHVLYSGCPKPTATADDAMAEDTDGLKYCVAHYKAGTLEGSTPLKFSYPQASKCYVACSARLLPGGSTPQCIVVGLDNLLVTSRDGNEDVLYDDFFMFGYVASSSALFLLRTSGLWGLKFPAPEVEEFAQPEEVPALPVGVTAANAVAHSKRIVDGSIANPVGWAAAAPVVDDASSGLLLAKAIARKRAAHRSFMAALCSTKIWDAMSQGDRRRVHEHGEKLAMCYALRKFQNDQHVAKGKPGHARQAHAFLAATLEEVVAAADRDLPSDTKMSVQERFFSHASQVQRLLELLSSLRDNPVKWSDTLQKFGLTPQTMVTFTNTVFLELFRSILAHRDVYGRSQALQDFGRMEWTRLDPFLQLISRQLALTHDVIADLAGANVKTSDGLLGATEAIQRLTTSSDAVLLDPAGELLKQQADLVDYVLEFTADDGRASPTAIALVQKYLMEFAVGPRKVADGRRRASPLAHWVREPVRALAEKWRLFDMLIELSRTAPEAPPDPQLMQQYLQRFEGEGFHTALFQHHLQHKDWRSLLAVEGVDQPLAEFLDRHNVAMAAVHRLRFARGLEPPAAAAELQTSAALFRDAAQRQLNLRERKASLSMAKLVLHLHSTTSPADTNSRLVMHDLNKELKVIKLQEVFLQEPEYLAPEPAIEKLSAAALTTAHRQQRQDKLQAALLLNQQRLDCDEAERTRCEKEIWAYIWQAEETDWRQLAQLCRDGISDPDMRQHLEGSLFCTLRYTPEGETLTRDMFHDLRKTGRL
**1863**	MAEPVLVSEEVPLTSTQQSSAAFKRFGDEYVRVGGSLAKGGTMTVEESRTFSQTPITTYQSGARAVSNTRILSTGATTLSSATSIVDGSRRYARSSAAVCCQPFDPCNSVVTRQLQTRYLDPVETTHVEKVPMEIEVDVEGYREVIEVPVERIVEVPVERIRTVPQMQVVEVPVNRHVERLVVVPELKERQVFIPKTIERVVKVPEVYEYEVPVQRTVEKVVVVPREVLVEVPVDKVTTVDRVVEKHVPVEHIVERLVEQERVVETQVKVQQIVEVPREVIKNYEVERIVERVVEVPQLKEVQVPVERVYERIVPVPKRDTVQIPVERIHERIVPVPQTNIVEVPVDKLIERVCTVPKQCTVEVQVEKVVDRIVEVPRTITQEVLIDMVVEKVVTVPSEKIIEVPVEKVVESIVQIPKVVTHDVPVEKVIERVVTVPHEQRIEIPIQRVVERVVQVPVDRVVERVVEVPHVVTREVPVESVVERVVTVPVNRVVEVPVDRYVDRVVQVPVEKVVNRVVEIPKVITREVELQQIVEKIVEIPTTKVVEVPVIRYIEKIVEVPVTREVEVPVQKYVEKIVEIPYERVVEVQVEVPVERIVEKVVEVPFTVDRVVEISIEKVIEKIIEVERRVEVPVEVIVEKVVEKVVEIEVPVIIEKVVEIEKIVEVERRVEVPFDTIVEVERVVEVEKIVEIPIEVVKNVERVVEKTIETSYDVTPVVEQKVSSFSASGGGSYSTSASYSTGGGYASGGGGYVSGGGGGFTSGGGGYVSGGGYSSGGGTGGSAFIGGGGSGGSAFISGGGSGGSAFISGGGSGGSAFISGGGGSGSGSRVTGGFSSSGALPGAAGVTGTTQLYSSQTSSLSGSTGAWGLPKQY
**1888**	MDGLLKACFVGGNVNEAATSFAEGVDHEPCLTALEPFLSEIKTAISNKKDNNQKIGACTVIGALTKVVGSAVEPYTIPFLETMLDLCADKQLPVQKAAKANCMVVVEELCRASTKQVVPIMVNGLGRSKGWQTKVAALEMLSALAARAPKDLSAVLAIIVPAVSECMWDTKKEVATVAKDALTKVCAAIGNSDIEPFIPAVVSAIANPTEVPDCVYKLAGTTFVQVVEAPALSIMVPLLIRGFREKSIPIKRLCSVITDNMVKLVDDPFSTEPFLAQLAPENEKVMEVSDPECRQVASKTNDTLKRIRDPSKAKASSAADFDATLKALQDLITEIFPTLKVTPFIETSLRWIATSACGLINNKQFYHESWTAAVLPNLGAFIDHHCPDSAQKINEAFLEKCVKELAPKEEADLDDEEGEDLCDCEFSLAYGNKILLNQTKLRLKRGQRYGLCGPNDCGKSTLMRSIANGQVEGFPPQDVLTSVYLESDIPAELADYNVIEFVRLDPHLKDKDMDKEIIPMLKSVDFTDQMLNGPVYQLSGGWRMKLALSRAILRGADILLLDEPTNHLDVRNVAWVENYLINLKDVTVVAVSHSAGFLDRVCTNIIHFNGLKLKKYRGNLSAFIKIHPEAKAYYELKAVGSLKFRFPDPGYLEGVKSKNKALLKMRNVGFTYPGTTRKILTGVSIFVSLVSRVAVVGPNGAGKSTMIKLLCGELVPQEGTVEKHPNMRLAYVAQHAFHHIENHLEKTPNQYVQWRFAGNEDREAIVKATLELTKEEEEKCKKPILWDGKKRVAERLVSRKQVKKSYEYEVKWEGSPYTENSFLSKDMLMQLGFDKLMQRVDEREAQSQGAFNRPLTQVNIEKHYTDVGLEVEYSVHSQIRALSGGQKVKVVLGAAVWMNPHIIILDEPTNYLDRESLGALADAIRDFGGGILLVSHHLDFTTALCSEKWVVDNGQLQAIGSPDEYFKGEAYDVQQQDEVTDGAGNVVKVDTKKKTNLTRKEKMARKKAKDAA
**2012**	MAAGTGAQQPCSSVGPRRPSTALHLHAGLDTGLPVVEALAQVQRTIGGAAVPPLAQEGFSIPTFTGEDLVLPTVASLAYLYARPGVITGAFDTVVAAPMYRMQMKKLDPKKIEIGRKLGSGGFGEVYLASVKDEDSGREVTIVLKRVKEFGEAETWMNERINRALPGVCARMVDSFEIKPTGDKKPSSGVFGLSGGKELWLAFDYEGEKNLAEYMQEPNFPFNLEQLLFGRRLNGPKSPERKIQTIREIMRQLVFLVSSLHNIGIVHRDVKPQNCIVTFDGRLKLIDLGGAADLRTGINYAPKQYIIDPRFAPPEMTVMQQSTPAPPPIAVATFLAPALWILNTPDKFDVYSLGVTFLQLCFAGMRSNDDILAFNDTLKDSFKYDLQAWRESEAKRNRQDYLDGFALLDLDNGAGWDLLKRMMQFNPRDRPSTTDILRSAFMKGTTLTAPMKSRGVVDRALEVVNQLVVQLYSRYTRTPTDQGIQEGTVLNVEDGQAVPESPDDVSRTQTWWEGRKRAVTIATRPPTLMERLSDQMKPRSK
**2212**	LDARSEEHFLQSSAETANLEALLRVRQKDGDLRREESRQQWLAEINIEPDEMYTGPRISDPVTEEELHALYNHIKTTPDDCVHPFYMLRLLSAAKKQFSGLPRVTEVVHTDGPHVTFVVVGDLHGQLQDLLYIIDRHGLPGPGRCMYLFNGDFVDRGPYSVEITILLLAFKLVYPGSVHLNRGNHEDMLINQRYGFCDEVLDKFGSLHMFNEFQDLWNLLPIAATVDGLALVVHGGLFRLEGVTLKHLKAIPRGPCNLQPRNFQETIMVDLLWSDPCEEPGHQMGLRGGNTIRFGADVTHRFLAQNKLKMLIRSHEVPHTNKGFDVTHNGRLVTVFSASNYCGHRGNSGAVMVFQRDLKYTFHEHWAPSLESFRVVRTTSPKRPNSEIQSAEVKLDVLRQVKKLVVENKTDLQWFWNREDTTGSGSVPIDTWRHGMNVVLNISLPWEKYEDFLIKKCMSTPDTVHYGRFLQQFQVRVKPEFAKWKEYVVKQVHDALVQADLNLADMVEVFDQNNDGYVDVDELSRVFRRLEIDVTKDQIEELLADIVICGQVDVIALIESMQIKRYANLDPSVQKIIDYIGDLLIGQEASETVRLFENIDENQDGRLQHHEFINVVQRLLVEDKAKGKPNCPEVSEEMLVQVTQAIDANNSGSIDFMEFVDVFHRQRSSMDRLLEQICRVFYKYQDSLFRAFQYMDVNGDGVLTPEEFADGLMTFNALVSEPLSASTMDAVVKYIDRNSDGFISYQEFCSAFYFVDSSTDVTEPMKQGHRRRFSKPADTLPKSVLERRASKGNLPHQFPDQFKATSSQPPAPQ
**2357**	MSPSSLHITTKPHSISVSSLKPASGGEFQEDSGRFANLNLQPMPQQAKAHLQVQHSAEERSLASSWTPVVSLLTALGGAAFAMAHLRHRADSQIAIAPFFGIVQPTNQTVADVRSSSSSTKTDKGADSSEDSMDRETERHRIDQRVVFNAERWRKHRGIDRYGRAVGTTLQSYVVRHLVWPVFGISLFAAGVCAAQLAYGVDQFSLPTSLFSLSAPTLGLLLVFRTNASYERWDGARKMIGLVKNRSEDLTRQVCLRFPADRTDLKEQMMRYIHAFFFALKVHLRSGPGGFEDDSNLIEDLTPVLKSKELELLLKATNRPAHVMHCMTEIVKAAKLEGAALYCLDQNITQLADVLGGCERILRTPIPLSYTRMLTRFLLVFLLLLPLALSVEVLKLDHSIWLTIPAVSFISFALLGIEEVAVQIEEPFSILPMEAYAEGSRRNTTTIFDQMAAVASMNIVEKAAPPSLPSSPVPSQAEPAKPVLAVAAAATAVPPAAPSTVAVVTAAPVAVTTAAAATTVVAAAPPSPAPAKAEPSTAKGRTAVAVAVQEVTQVEAVVEKHAVVSEHAASGQHEAARKSVVHVAAAPAETPLPVPVSQPQAQKAESSNIARYEPSPKEVVLELQRGNGRFWMGVSERNEMNAMERRSLIVAQYPKVCILSCSDSRVPVEIIFDQGLGDVFVVRMAGNVLNASAAGSIEYAVKFLGVKVLLVIGHEGCGAVKGAITMSPTDIAKEPSDLQEVLRSIRADLDVKALDDISDVRAREREAIVNNTMNQVQRLQSNPLIAPQVEKGQLLVVGAFYEISSGIVDFFSLDKNGFLVDV
**2385**	PARNQIAKNPTNTVYDAKRLIGRKFKDKEVQNDLKTWPFQVVAGDNGDPCFQVQHCGQTQQMRPEQISGLILAKLKETAEAHLGKQVRKAVVTVPAYFNDAQRTSTKVAGELAGLEVLRIINEPTAAALAYGLDRKTDKSQLVLVFDLGGGTFDASLIAMDSGVFEVKATSGNTHLGGQDFDDNLVKYCIQEFKKATHLDMSENAKAVRKLRTAVEKAKRALSSSITTEIEVDAIHEGEDLLVKVSRAKFEQLNLKLFEQCIKEIDKVLTDASVKPADVDEVVMVGGSSRIPKVQEMVAEYFGGKRLNFSINPDEAIAYGAAIQANVIAGSAEAKAHTGDVLLLDVAPLSIGLAADGGKFKVLIPRNATIPRKVSEEFTTVDDDQTCVEVEVYEGERPRASANHLLGTFELDGIQKAPAGEASIMVTFNVDANGVLNVTAEDSSSPGGKKAEITITNTNKLSSDQINAMVEEAKKFARDDQAFVKLATARAKYEEYLYDLQKLAEEREVKESLARALEAQAEWQEQLLEASYEDIQDKMDQLVEIHQRMLETKEERAKRKRAEQDGELE

	DDAFSQPMLGAADAPGDDFAPEDRPDEAEEEEEHSQPKKRHRKTVKTAVKTVAADTPGDGDRIKVEKKEKKERKALKRKGEGSAGEPGPADATADSAPAQPAEAKPEVDAT<br>DEQPSAHPNPDAKPTTGAAGSPKKPGRPSKPRASKPGKGKDKAADGAAAQAEGGSPTADGEATDAPQDEQEKGVATAPEDPVAKKPKVAKGRKASKEIVVADDES
**2459**	IFFRQSIINLTAEFFRNNKLVRKNDIAELKGTRLAIDGHFWIRNLQFNEPYQMAMGGVPLGLQTGIDAAIEKLTKYDIKPLFIFNGIKLPPAPPMNSDGKSINSGVQGFGLDQYRR<br>REEAWRHYQKNDYEGAKKEFREAGYGAASGDVLDAVLRYFQQKNIEFLRAPYLAWAQCAWFCGQKDRVASAVWGSEEILLFIGMVQFDKLILQLDFNSGIYEWVSLTEICDA<br>LGQGTSAGKITVSQFIDCCILVGFRGGWIQGTQYADDDNGFASTMEMDSIRDGIGSSLQGLPQNPNAIHFAQSFQDIEQVARDLTSFRSGPSLLEYKVRTFHLDTMYLDQVLE<br>QFLRIKNLILHHIILNLECVAIPISMHNNTHPEKDRNYYSNSMMSMPQRNVINNIVPNNLHTIIGPRLPNLVYFFLSIGLVSPQVIYNVVHNTMMDYLPTIDSYEYRDLLQKIVPLR<br>TQIAYLLINCMMRKDEVYWKVVPHGGRRPVMSYIQWHTEKTIALHTPPELLLDDWDVTWTPAITEQMPKDDRGGLVMNFCTVLPFAKCALKACVSANVYGPPRTYNQFEE<br>VVCVILLKSLDLLGYFTHPPQQKPLGGDESGTSLFAEALSDVHYRYAEQGVLFIELIRTGALNCQPFKPPYSHPTDLVTITLMTDPNVLLISRVLSIMPMNLKSQEWGSNIVVRDLC<br>AFHVLFKSLHKTLRNLCEIIMMVMFMDNRVKPNNMQMDSVTLFTELGRLLPFNDSVNTAMGVVVYYLLLRQHNAQGNTPQERWQTLQMEKTFPCCKDIRGDLSRAYGFW<br>HEAIRVVAKLEKGSAISAPLYQQFVDADRLLCNTMAAFSIPPHPR
**2508**	MTAAPAMMIQRPSSAVAATNVQAQNVQGSGYHQVGATFAPIVLGVGAITAFVAALLHRGQKPVAMAATTGERLLWIPNATPPAHLTGEFPGDRGFDPLGLAKDPKVYQR<br>MRISEVFHGRLAMLGIVGCVAPELFFSKGAWFDYSDYDLNRLGLIALQVIAPLEYWRGNGGFSWDGNDGPDRSYPGFDPLGLTNEETKLQEIKNGRLAMTAMLGLEVQSHIT<br>GKSPLTNLSEHLSHPLSANLLTGGGSLAMFSTAGQKTDRRPLWFPGGYAPSWLTGQHFGDRGFDPAGLAADPKVFERMRVSEVYHGRLAMLAIVGAVVPDIQGKGAWYEA<br>AQSAGIGVNEVAVFTAAYGIAEVARGLKANSDPTSRYPGFDPLNLTTDYTKEAEIKNGRLALTAMLGLEVQRHVTGVSPLVNLVEHVKHPLGHNIAESVMHQWPVAMFAAT<br>GHKDGVWFPGATPPAHLTGEYPADRGFDPLSLAADPTVYARMRVSEVFHARLCMLAIVGAIVPEVLGKGAWFEAGNSVDGIKLGFIAMAIAAPTEYWRGNGGFNWDKGA<br>ADRSYPGFDPLKLTTDYTKAAEIKNGRLAMAGLLGLTFQYLATGESPLANLAAHLANPVGTNITTTLAMYSTSGEKYRGLWFPNITPPAYLTGEFPADRGFDPAGLAADPKVYE<br>RMRIAEVFNGRLAMLAIVGCVYPELLGNGVWFEVWNKVDFYRFALISLQVVAPLEYWRGNGGFGWDGEEKYDRSYPGFDPCNLTTEYTKAAEIKNGRLAMIGMFGLEVQSH<br>VTAQGPVANLIEHLRPCSWSVRLESYTPVCWHAPGFFQNFQRMGQRAPFQWGVTEPPSFIHTSATVNEGWWGSES
**2633**	LISVDRTQTTERSRLTDDHKDPFVTLLKCYKAIREGANAFLLAEYTLCSIFIVIFSGLVLVLTAKTGAKFEWDVGALTATSFVVGGFTSILSGWVGMQVATYSNARTTISATQNGPK<br>GWTDSFNTAFRAGGVMGFALCALAILILYGLLNLFEKHFGSASTGEAKLLFECIAGYGLGGSSMALFGRVGGGIYTKAADVGADLAGKVVSGIPEDDPRNPATIADNVGDNVG<br>DVAGMGADLFGSLAESSCAAMVISSQSKHLVDNGWACLMVPLAISAIGIVVCAIASFLATDIMPVRKEEDVETVLKVQLFSTALLMSGAILPMMKYWLPETFSFTTYLDSNGVP<br>IPCHWWGAYACVLAGLWSGCLIGFITEYYTSHSYTPVREVAQSCETGAATNIIYGLALGMKSAILPIFLIAGTVFISFELVGMYGVALAALGMLGTLATCLSIDVYGPVCDNAGGI<br>AEMSEFEEGVRAKTDALDAAGNTTAAIGKGFAIGSAALVSLALFGGFIARVSGEHREQVTINVLRPTTFAFLFIGAMLPYWFTALCMKSVGTAAMSMVKEVERQFREIPGLLDG<br>TPGHGEPDHSRCIKISTDASLREMVLPGLLVIFAPIITGSFFGIESVCGLLVGSLTSGVQLAISQANTGGAWDNAKKYVEKGCVRVGGQVQGKGTDLHKAAVVGDTVGDPLKDT<br>SGPALNILMKLMAIISLVFADFFVSVNHGRGLFNLNSALNL
**2977**	MEFFRTNNLVRRNDIAELKGTRLAVDGHFWIRNLPFTEPYQMALGGVPLGLHAGIDAAVERLTKYDIKPLFVFNGVKLPPAPPMNSDGKSINSGVQGYGVKEYHRREEAWRK<br>YQKNDYDGAKREFKEAGDGVATDDVLDAVLKYFQQKNIEFFRAPYLAWAQCAWFCGQKDRVASAVWGSEEILLFIGMVQFDKLILQLDFNTGVYEWVSLNEICTFLGQETN<br>DGRITVSQFIDCCILVGFRGGWVQRNPDEDSGVVASTDMDGIQDGIAGSLRGLPQNAGAVRFALSFQDIKDVAFSLPSFRSGPSLLEYQVRTFQLDTMYLDQVLEYFLRIKNLLL<br>HHIILNLDGVAIPISMHNNTHPEKDRNYYSNSMMSMPHRNVINNIVPNNLHTIIGPRLPNLVYFFISIGLVSPQVIYNVVHNTMMDYLPTIDSYEYRDLLQKIVPLRTQVAYLLIN<br>CLMRRDEVYWKVVPHGGRRPVMSYIQWHTEKTIALHTPPELMLDDWDVTWSPALTEQTSKDDRGGLVLNFCTVLRFAKCALKALVSANVYGPPRTYNQFEEVVSVILLKSLD<br>LLGYFTHPPQQKPLGGDESGPSLFAEALADVHFRYAEQAVLFIELIRTGALNCQPFKPPYSHPTDQVSITLMTDPNVLLISRVLCIMPMTLKAQEWGSNVVVRDLCAFHVLFKSL<br>YKTLRNLCEIVTLVLFMDNRVRLSNVRMDSMFFFTELGRHLPFNDSINPAMGVVVYYLLLRQHNAQGTTPQERWTTLQMEKTFPCCKDIRGDLSRAYGFWQEAIGIVRRLEK<br>GSAIAAPLAQQFGEADRLLESAMAAFSIPPHPR
**3011**	LLLALVAAGLGLFPPASAGAWQAWQYLRPQSTPSAVARQKQWAASLQPGSLVRHAFPPGSDITASEWGGEGAWPTSGSNPRGSQPGNAPANPWRPAGQSQPVAINTGL<br>PAEGWGAPAEGAGAGHSSQSAWDAGWAGITAAAVGTALVAGAAVGERRDASFGPASAPDRTLYLAQCPHNVTEDVVRNHFRELGPEAIVQVTWNMRGGIFDGSGYVEF<br>RTPEKALEASMYAPIPVGPVSCLHRLLTKLHTDVGDADMAIRDARVVFHGGCPDSITDDHLYRHYSVLGEDAISSVRWSLKDGRFRGTGFVEFKTIDLARRALELPPLIVDGKAV<br>TRERNGAGGKVGLPLRKARERASQLLTNIHQAPRFSFSQSQPAWWSAEWLAQNPFPRDFSPTLQYFERDPQAYQTFFREFNLSVEPATIWPVLDLHEVDFGGFINAFLMESFS<br>KPTPLQSVSWPAMLSGRDLIAVAPAGSGKTLAYGLPCLTHIMAQRRSEEREGPIAVILAPSREHVLQIHRDLERFQTVLGVQIGAAFGGDDPAASRFMQATKLVSGCEVLVATP<br>GRLLDFVESGVTPLARTTLLVVDEAPQLLARTLQPQVDAVLKQLRPDRQVAFFGASLSLPEQQFVESVAPDPVKVVLGKATYPPVNAGVTQRILFPEGDRGDREDMLVDVMK<br>EVIDFNPDDSRMLIYVLRKDYLRGIVRGLRYIFSPRVLSLAEGATQLDRVTALASLNTNPVGMLVATDVVSRHWEIEELAYVINFEMPPDIATYIDRINHLGVDGKPGMAISFFTA<br>KDEPLAGDLVRVLQQSGQPVPPRLLDLAARAADPIGGLGTAAGWGPAKAPLPPLTEREARLKEENKTQLIEMRRREAVRAMLWP
**3087**	MPKSGGIQFDLPPRYRLVKLLGSGGFGHVCLAYDTRTVLEHNYVGDGRRKAFKVTFKHITIDDGDTISAMINGERAPFRLLTPPGEFWPFYVEFEHPPPRGAAIKLRTLERVAIKR<br>IIDVFSDMQHCKYTLREIKLLRFFDHPNIIKLRDIFLPFKTHQLLPGDKWEELFFVNELMDNNLRVIVKDKEQIIREEHSSYFMYQIFCGVKAMHDVGIIHRDLKPHNILVNTDCELK<br>ICDFGMARKEEVSIDMSDEVQTIWYRAPELLVDMRSYSKPIDMWSCGTILGELLVRRALYMGRHALHMVQLIIQMLGSPAEEDIFFPENENRVRAFIKKLNVPPIDWKKWAP<br>HASEAALDLISKLLVFNPAKRITIEEALEHPFISLYRDPGEEDDMESDVLSPSESMGSFERNTSIDVQYAQTLPAENRAEFRLLMSNIIDACMVDRPDAEKVAILNDIVARVIDLCPT<br>TADFFAQLGWRGDRFFGPVNFQLLRQYIQKWERIESKLDSQTHDQRKQHKAKEGKAKISQFDDTFEEMFKDLVTPSQEQKVMDQVRKLLEQEIEDFSREHPEMATMPPPAG<br>VRPPVPFRFGTGGGDPTETPAHIIEMADTCYTPVTPCQGQDWGHA
**3482**	MSSAEELTLVSNGVQADVANGVLPNSLVPHIVYSNDDLPSVGKLFIGQLPDLFAEQDIRQIFENFGRLEDINVLRDKTDRSSKGCAFMRFSDPAVTLAVICLLHGKFRLQGNLRAI<br>EIRFAQTPAAKAAQPFGMPGVDGSNPYGPGYDVQAAWAAYYQYYMAAYPAYYASLYGAAATGAAAAPMADGQQGSPAPGEYPAGSVAPGAPAADGSQNGASSTVPG<br>WPTGYSYSGQPSTAVVSYDPTAAAAAAAAAAAAAGGTPPPAAPYPGYSAAPMAGYPGYAGYPPPTTTATVPPGYPDPYNSTQAPGYPPSGYGAPGQAPYPSGATPGYPAGY<br>PAPGQPPLPTDPPPQQGGALGYDPSAYGAPPAGYPGAPPAGYPGAPPAGYPATPPPGGDYAAPDWAAGGFGGKGGDFGGKGGKGMEFGMGPDFGKGKGFGKGDFGK<br>GGKGGEFFGGRGPPMMEGWGDSGFDEYGGWGKGGKGKGMDGGMSMGGEWTGISPEGAGVVAFIPWQWDDHEVRSRFAHFGNIVRVKLPKPGTAFVNYDNVPSAD<br>RAIAEMEGVKDGTWTLRVRKLVARAKPY
**3489**	SFPALPTAGLKALITPGRDLASGEFQCNNALQLPKLLKAANVAGAPSSPHDVGKCLVEHLPANDVIEGTTVAPQGFINIKIRKSFVADRLYDILKNGLKPPPQPRQRILVDFSSPNI<br>AKEMHVGHLRSTIIGECIARLFEFCGHDVLRVNHVGDWGTQFGMLIAHLKDTFPDFRTAPPPISDLVAFYKQSKQRFDSDPEFKDRAYQEVVALQGGNEDNLQAWRSLCAVS<br>RVEFDKIYQRLEVRLEEKGESFYNPFMPSMVQGLLDRGLAKVNDGATVIVSADQKKLDEVTPADVERILQYYYLNAPLSEQEGLLALLRPGGTVKANAEGVECLNIGTAKKEQLI<br>PTAEVSQVALDRQLLGKVTKALAEVLAVTTKTKAGTATKLQPEFLALLRTVPELVSAAADGEVLAVPKFSHPFMVRKRDGGFTYDTTDLAALRYRLQEEKAERIVYVTDIGQEGH<br>FNMLFAAAQDAGFMDPAVHRCDHVGFGLVVGPDGKKLKTRAGETIRLVDLLDEAPRRSYEESVKREEKKRQEAAEKGEPYEGLTEEELRANSVVIGYGAVKYFDLRQNRVSDY<br>RFSYDDMLNLEGNTAVYLIYAYVRIRSMARRAGIAHSDDVLAELLQAHPEGLVVSLGEDSEWVLGLHLMRFSSTVERMAEELYPNLMTDFLYQLVDKFNGFYNNCRVLTAEDR<br>DGKLLLCEATAKILKEGLRLLGLGVVEKI
**3743**	MNFNFLSPEYTQPTYAPAAAQAQRAAGAAPPSPHPYRQQQDDYRGQRQAEELAYRQPAAAPRSSTPYANGAAQHYPSQQYHQQPDHTVRSYREESVVRPGPMVMRTSV<br>HNVAVTEAQLESLRQGASLSRLGTVASAPNTPSRAPATHVSAASVSTPVHYQGPVSVTASASPPITQLATAPFGTTTTTFSLPGTTTSSFLYSAQPLAAPTYTFQAPTTTSVYAAP<br>TYLSSSQTVLGGQSFISGATYLNPTTAALASPSIYSLDPVTSASVAAASLSRGVLAPGTPVAFPPAASQVLVLGSDYAQATHFDSPATARAALTTDPRFHTPVRRLSNSFGDYQAA<br>QYTQPTSYGQQQAGSYGAQSPYAQPTTPSAQYSGLANGYGYAPQQPPQPPQPFAGGAPGGPAPYPNQYGGPAPPPSGHPYSAAPPQAGQYAGGPPTAYANPYGGPVAA<br>PPPSASLGQPYPSTAPPPSGPPAGGFGYGGPAGPSVEPSWGQPPAQPQPQAPPPAAGSQRRSSLLSKLFHWMDDEAPGPTAAPIPRENYTTLPAEQRIYLTSKTDPRDASFV<br>EPVPLLVPADNAKQRIKHIVGSMPQSRIFVDNGPYLRFELVSPAYGAVDDVELQFDAQHRVIHTLLTQSSGPPDPQAARARLQYLFQCLQDDIRQYPHAMAELNRPPLPKYGA<br>PSAAPYGGPPQVSSARPPAPAESYRPSGPPVPYSYR
**3760**	MTLRPWVFLLLLLLLGGVSAADDEPVIGIDLGTTYSAVGTWHLGGVEIIPNEMGNRITPSVVGFTEEDRLIGDGARTQQPTNPLNTVFSVKRLIGKKFDDLEVQRDLQLLPYKVV<br>EDKESGACKVQVTYKGDIKQYSPEEISAMILSKMKAVAEAYLGREVKKAVITCPAYFNDGQRMATKDAGTIAGLEVLRIINEPTAAAIAYGLNKKGERHILVFDLGGGTFDVSLLS<br>IDSGFFEVVATSGDTHLGGEDFDIRLVKYFLSQLKKKHGIDIRDDPKATARLKRACEDAKRMLSNQPEARVEVDNIAEGFDLSEKITRAKFEELNMDLFKKTLDPVKDVLRQGKLE<br>PKDIHDVVLVGGSTRIPKVQQLLKEFFKGKEPNRGINPDEAVAYGATVQAAVIAGKDEIQDKVLLVDVCPLSLGIETVGGIMTKLIERNTPIPAKKGKIFSTNSDNQATLLIQVFEG<br>ERAKTKDNRLLGKFQLNGIPPAPRGKPKIEVTFEIDENGILQVGAKDKEGGVSEQITINKDKGSLSPEEIERMVKEAENFAQEDKKLRERTEEKVALETFAYNLRLQITDEDRLATK<br>LDAESIKKIDAAVEEVITWLDKNKDPEADESKEKYKFLESVAKPIIEKAYLAMKDEREGKAEKKGKAAQEEEDDGEL
**3780**	MMNPFLQSGNAVTRRLGSLNEFLLGNPKRSSPVAFKRPLYDGFGIEGNDALDDLDNTINYELDQFRSGKVWLYGVVDTQTKVLRRGLVIEDHLLSCAHPAPSPAFTHTSPRKT<br>HRLQALCTALDTDALPRKLFINNVWAESKTGLKYATINPATEEVICEVSEAGEADVAAAVAAAKAALEGSWRAAGPYARSKLLWKLADLIERDVELLAALESLDNGKPFLESSTV<br>DLPLVIQCFRYYAGAADKLHGNTVTLGGPLVDETFTAYTLHEPVGVVGQIIPWNFPLLMAAWKLGPALAVGCTVVLKLAEQTPLTGLYLGKLIKEAGFPPGVVNILSGFGDKAT<br>DGSIGAGLALLRHPDVNKVAFTGSSSVGRIVMRECAKDFKRVTLELGGKSPNIIFPDADLDGAVAGALISQFFNQGQVCCAGSRTFVHEDIYEEFIDRLQKATKARQKLGDPLHP

	ETTQGPQVSLEQFNQVMNYIEVGKKEGAKCLFGGERQQGSKGYFIQPTAFVDVQDGMRIWTEEIFGPVMAIAKFRTMDEAISRANYTVYGLAAAVWTKDLGTAQYMQRRL<br>RAGTVWINCYNVFDANMAFGGYKSSGIGRELAQYALENYTEVKQVCVKSAPRF
**123**	MAAEPTDSALKRKKKKAKPIIPSLEGAEAADPESAPPLAGRARPRKRAQQPDAPAGARPAKKRRRPPPPEESGSDSDDADILAEAEAELRAEGVQIHGDQQRCPRWAKQDIAK<br>AMDTIKRRSEDDTTKVFYARCPLNITEHHLYDHYKNLGAEAIKDIKWGQGRADLGSQFKGWAILTFKSTSLAMQAAAMKPPQVNGQELVVRWMGYQPLDAKRPSQLLLEM<br>RRKREAIDEELGVKKGTGAWKKDWLQGRTLPPVEWTEGKLADKAFERDFYSTCVKAVNRPAEDVQKWIQSNAISVPADSPPPIMEFSEVDFGEEVNKVLRKKYRSPFPIQSIS<br>WPIILKGRDCIGLAQTGSGKTLAYALPAIVHLNAQPRPTAAEGPVVLVLAPTRELIIQICDELGKYQMAGGLRIGLAYGGSDGAADRTTQANVLMRGVDIVGGTPGRLCDFVEA<br>KILPLGRVSFLVIDEADALLEMGFIDQTSALVMQTRPDRQTVMFSATWVRDVHEMAETVLTDPIKLVVGSNKEKAVEANRDVEQRVVIVDCMKDKLELLTTTVQQLKQKSPA<br>SHRTLVFVGQKKFVAPVSKHLLPRFGKNVYTITGDLHQTVRENIIRRFRDDPRGILVATDLVARGLDIKDLRCVVNFQLPRDIDQYVHRIGRTGRAGEKGMAVSFFCRFSPMDC<br>RLAQDIIDCIRRAGQQPPNELLQYADGVTADGQKGRRKKRRVEGGKGEKGDPPEEDDAEGDRWGPDNPTERLLPSVPHTTATPAAAWTPAAATP
**4401**	MSAMLGLEVQSHITGKSPLTNLGDHLSSPFTANILTGGGSVAMFSTSGKVDRPLWFPGGYAPSYLTGQYFGDRGFDPAGLAADPKVFERMRVSEVYHGRLAMLAIVGAVVP<br>DILGKGAWYEAAQNAGIGVNEVAVFTAAYGVVEVARGLKANSDPTKNYPGFDPLKLTTDYTKEAEIKNGRLALTGMLGLEVQRHVTGVSPLVNLVEHVKHPLNHNIAESVMH<br>QWPVAMFAATGHKDGVWFPGAQPPAHLTGEYPADRGFDPLSLAADPTVYARMRVSEVFHARLSMLAIVGSIVPELLGKGAWFEVGNSVDGIKLGFILMAIAAPTEYWRGN<br>GGFNWDKGTADRSYPGFDPLKLTTDYTKAAEIKNGRLAMTGLLGLTFQYLATGESPLANLAAHLANPVGANITTTLAMYSTSGEKYRGLWFPNITPPAYLTGEFPADRGFDPA<br>GLAADPKVYERMRVAEVFNGRLAMLAIVGCVYPELLGNGVWFEVWNKVDFYRFALISLQVVAPLEYWRGNGGFGWDGEEKYDRSYPGFDPCNLTTEYTKAAEIKNGRLAMI<br>GMFGLEVQNHVTAQGPVANLIEHLRHPLAANIGANLAHPWPPVAMFATTGHKDGVWFPGAQPPAHLTGEYPADRGFDPLSLAADPT
**4567**	MAGIGGLQGSRISLISKSEIRYEGFLYSINPDENTVALRNVRMFGTEGRKKEGPQIPPADQLYEFIIFRGSDIKDLTVFDAVQKQTAQPPDPAILNAWQAPSAPSAPSSGWQGPP<br>ITFASRGPPRDEYLGRPAPRGPPRDRYGYDQDRYGYDRRDRPDNRWDDRREDRDRGVRYGGRDDRDQRYGGGRDYQDRGWQPRGGYNDRGSYGGARYNDRGSYGGPD<br>RRDRDWEPPRGGKAGRDDRRDDRRGSGKDDRRGGGKDDRRTGKEDRQPAKGDRQRGKDDRRPAKKDQKEGRPERRPEGKKPRSESVRSNADQHTGRNFKVDDSGAAK<br>KQYAEEFNFEENLKKLDLTKVAQEFQEKASESETAEDKKVKLQFTAAKGVYNKEESFFDQISCEALERKITGPEPKKVDKELREQQKQLDRETFGEAGAGFGYGYGPRAGFRGRK<br>QRSNFKTGGGGAGGYSNY
**4707**	MLRLSQACLLLGLVWGGNGALQPTVEVEVTAGRGGPIVLHGVPPPAQDSALLADSLAAASPLRLVEARPANACTALALSPPPRGPFAVVVQRGLCEFGLKILNAERAGASAVLI<br>TESLTTLYNFSSGEPRLQASEGGPCLLRCDLGEGEVPLSTPKADILKGLAGSTCAQSERCPSHMCGVSGEQVGDSYRACCLPDRYVMMDLGNRTARRPAIPAVFLGASAATDLE<br>RALRGGGPVVIRVADAGAGLGWLVGAFLIWVLGCSVEALSTWLSAQEEREWVATGGAGAAGEKRQHGPEEPVVMTVETAMGYLLVATVGLVGLFLLVQQSARVVVFVMI<br>GVFSIATLNVVTSLGTYPVVHRLAPQLDRQMVTVPLLGQSTVAWLVGFAITLAAVVYWIVTRHGPYAWIFQDTFAVFVCCQFVQALRMNSIKVSYALLVSFMLYDIFMVFLSP<br>LIFKSSVMVTVAQAGSGSISISKTNPFHCERIPDERMPMLFMLPRFGSEGEYAMLGLGDVLLPGLLLSFMLRYDTLARAKDATGGCCGLCYLCRRYPYWTVGVVGYVVGLLLAF<br>LANTLGFTIFDVKGQPALLYLVPCTLIPLTLLALVRGEFMVMWEGPPELRQAEADAAAATADHTPEARGLLKQEPI
**4779**	MAYDRERDSERPRDRDKERPRRDNRDVTPILRVAGSSEAKNMATAIERRILDFRAVKLEAVGAASVNQAAKSIAIARSHLEAEGKSLAVHPEFIHIPSKEEGQGDRSAMQYFVF<br>AVPRSRTRRYDDDDDARILRVSQKSDAKTVATSIQTRMRLDGSVKVQAVGAGSVNQAVKAIAIARRGLDDTLRTDLACYPEFIHIPAPNPGDGERSALRWTLFELSQGRKDR<br>ERDRDYYERDYDRDDRGYGRDYDRDYYDDRDRDRDRPRERERERERSDRDRDRDRDRDKDRERDRDRDRDREKDRDRERDRDKDRRPAREDRRADRLDVPLREPPYRADA<br>EESKVQELKVAATSDAKAVAGAITLRLRNGDPVRITVMGPSSINQLVKALAVARTYLVEDHADMAVYAYFIHLRGSSDDEPERSALQITVYLTHEHKFDEEQTAEFKVAGATNP<br>KSTAGAIANGIRGSKPCKLEALGPKAVGQAIKAIAIGRANLEQDKTDIFFVPQFRTMMLSNGRRSALELLVLPVAPKS
**5030**	MRPAAPRESSPPLPVGWEKKWDEKHRRYYYINHIDKSTTWTAPPPPPVVAAPPPRLVPEYKPPPPAPAPSSGFTANVSAPAATYTPTPPRPAFAGRKRPRGPPREPPVPVREP<br>PVQEPAPGSIHSEVFSQRGPVTRNPALSLNEMKANIPENPKALLHPDVQGKLDYFLAHGPFGREDVDQRFIDILADVPPIRAINCLEYVLNSNDTNGVKNPSAYLVTMLKKREFP<br>PPLHVDLTPPLLVEPVQALVDNICIYEVLRREDFTQLLVEQLSLLGAYGAKALCDFLAADLDRVHRPALLLQAIVNVYCEEYLVPAKGAAKTDRRPPRPVESRPPRPIEPYSEREPR<br>QRTWAPHDGEREHQGPPAHTNAAAMPTEPPTYQTILHPGVQARLDTLFAQRLMLREEVDEWAIQAIASLPPMVGFNLLQFLRESDIQGRIRNKSAYIKARVRNIQERGPGKV<br>VEVDLDQVAAEEAALPYEVQVKLDECFEKEVLIRQDVPQEVIERLGRLPEATALDALGRLLAGEADEIPNRSTRLLILIDAVDPEGANPEGYSQVEQPPPSYTPTPTAATPAQGQ<br>GKPDKRRQLYVTKLPQPPNADFIRAVFNDFGTVVDVKVVGVGAFVRYETHEECQAAIRALHNITILEGATEPLQLLLT
**5198**	MEILKDFATTLRALRTTSPAFRETCSRLTVLAAELEAEQRENVVSLLISQVSTSKEEDVKLAAWRVLDQIVTVVGRGYLPGFGEHLPHLARYSLPPTPEYSSVVYGWELKRLFPRETI<br>ESIRKGLESGFGKKLEEFSKSEAPAAAGPRAPQPTEAPPAHGWKEHVSADGKKYYHNARTGESVWERPAEMDAVPQPSPAAPRPPVAPVAAAVGSAGAWQELLHSSGRRYY<br>FNAVTQERTWQRPPEMDGPAAGQPGPPATAAAAAAPVSPTPTTAAPTPAPQDVWVEGVSPQGVKYYFNKVTKVSTWDRPAELDRPPPPPKPEAPAAPSVWQECTAPSG<br>DKYWYNPVTKVSTWERPAELAAAAAPPAVPAAVAPAAPQTPAAMVAAAPTTAPAPSAAALAKASMWQEARSPDGIPYWYNRETKVSTWQEPPEVAAVRGTPATAWQE<br>GVSPQGIKYWYNRDTKVSTWERPPELGPEPVATPTPVSAAQPAPIAQPAVYAQQPASPIPTAQPDATPDPKRRRVDAPPAAVEPAAALAAMQPFAQPVGPVPLHAHIPPGV<br>PMQGPPMHAHLNPYAAHQHMAHLAANGYAAHAHYLWPMPPGAAMYPPPMPMGPPGPHPHHHHQQHFFRGPPGPPHHHHHHQHQHHHHQHQFPPHMAHQHH<br>HHHQHLPPGPVPATAPPNQTRPPFAGPGQPSPRGPPTPTPTPT
**5340**	MTLAGSLCGDARACSTLLELLDPAKRTTHASSSFSGADITIAFTSVPEPGPSCAVLALVCHHQSLSSLERLVNDTIPAHRELLDKAPFALVTVPKSSSPTPAGPDKATAFAELLQTVV<br>GALAATEGFEGHLYVHDGPTGQLLQQTLGAALASPRLLLWSRRQQALTPRGTAMLHRAFWLFDADRDGELNDVELRSLAAAFYGRADEDDVVALKSLVTTASHGLSRSAFLS<br>LCEALLAQDKVEEVWQMLHSTGLDSTGQPSGVKDLSWLHPKPDKHKVLYEPSPIAQRFLEELFLHGFPRKRPADPWPGWAVVPGGVPGPIADPWYPDRPAPVAPFRAGWD<br>DAQADTFLAHWSYRCLADPETLIRYCYYWGYTGQAQDLVVPHGRGGPKHAAAKSVIQVLVLGGSKAGKTSLIQHLAGKRLPDLQIYRPTRRPLACITGKSFSEGEEKSRATVVY<br>FELVDEDVNAFITDEKQMRTIDVVLMLYDGSDPYSFSYLQTKQRQMLACGTGRMPFIYVMTKCDLPRANQLGCRPQEFVRSLQLSWPPVFVSVAGDPRTTSGPLSNEIAMLA<br>EFIVETARHPECATAMLEEESGEWRRLLPLAAVVALGAASLALWLVRRRGALR
**5712**	MNLNLRDEEEEALPFEGIDKAGALHESKSFNQASLDPKKCKGTLIKILYLLSMGESLATTEATELFFNTTKLLQSQDATLRRLVYIMVKELSTCAEHVFVASNTLCKDMNSTNDMY<br>KANSIRALRKITDSSMLGPVERYLAQAVVDKSDIVSSAAIVTGIHLSMTHPELVKRWSGEVGEALKQRGVMAQYHALALLHKLRKNDKLSVSKFVQSVNTIPIRSPMALCLLIRM<br>CAEVLKEDFDGSPDLSQFVVNSLKHSSEMVVFEAAKCICSLKTASSKDLTPAILVLQLYLTSHKPVLRFAAVRLLNKVATTQPLAVTTCNIDMESLISDPNRNIATLAITTLLKTGSEF<br>SIDRLMKQINTFINDIADEFKIVVIDAMKLLCVKFPHKHNVLLTFLSDALRDEGGFEFKKAIVDAIIKIIDTIPQAKEEGLLHLCEFIEDCEFTMLSQRVLHLLGQLGPTTSNPHKYIRYI<br>YNRVILETPAVRAASVSALAKFAGLCPPLRQSIKVLLQRVMQDNDDEVRDRAVFYTSVLDGKEEDVQTFIMDVATNTESRRSKMLKEVDDEVPLPLAEDGAAKGEGATAAAAP<br>GAVEQQVRSHKQIEKAVKALLAHPKLKKLGRPHKTSEPEHITDTESEFVVTYLKHAYPGKVVFEFRVHNNMDDVLLENVT
**5897**	MPDDNGEPPPAINWSLLDDDQEWRRREGSKSAPPKLNVSAFPVPDNLFFDFGQKQGADGDEFIRNGPEKGTGNPPLNAAFHDFINSVTKLTDSPEATPSQSLQSSFSSVPNT<br>TNASFTSLLLKFSGSQQPSRLEKDRRGVPFALVPDSPAMQPADGGALPIRIPGFGTSPQSQSFLLGTPPASLASLGGEPYFVNNSPSVGGGLMSGTTLAAPQASWPRPAFAEG<br>QAGKPKKEAVMKPKNQQRLTFQDSKEQPIGQLPKGKATTEMAQGNSLRPQVPADATRQGLHHKDGRGGVKGAAAGTVERRLFPALEGEIDGHLSSLDEVIGNIYPTAKDQY<br>GCRFLQKMLEDGSPQHLQQILEEVYDHCIELMTDPFGNYLMQKLVEYCNDEQRTIIAQKVAPQLVNISLNMHGTRAVQTLIEHLSNPVQVQIIIGAFQASVVPLIKDLNGNHVI<br>QRCLQKFSALDKQFIYDAAIPRCMDVATHRHGCCVIQRCIDHASEDQRAYLTDEIARNAFSLVTDPFGNYVVQYVLDLNMDQMIRKICSSFIGSFAGLCMNKFSSNVMEKCLQ<br>LAPDDIQHEFIAELMDPSILPKLLQDQYANYVVQTALTVSKPHQFGQLQEAIRAHLHLVRNTPYGKKIENKLNRGAGRGGGGGGGGGSRGKNRAGG
**5947**	TTVLLFAVQAERAAAHARETSAALDSKNGIGKGGDSGHVQYGCEKLKTCKDNVLDKWMRSVKGTVQRPTLLGTEMTAKANYSDLFDALVATLESRADPPNDAELISFCARLM<br>PHTQVAHKQIQDTVQAMLLFEEAAGNVLKDCFNEPGKGDDDQVEMDIAIQRVLLRAEDCLKVAHSRWGREGTTGVSMMLAEARIAFGDSREILLEALDNVRSLIPKAVPFQ<br>GLEDITSIKKMTELAVEMEQVAERLRLVVSQVAKGTAIDKAAAENKRNDIRLALVSLLRQKHEDLSERWLKTIVQRVPGYKAAGDTNQGAEQVSRDINKRLIAAMIERLRPGGD<br>SASLVYSAQKLVEMRFTKMKIDAVQAATKLFEDVCMDMYHELAQQGPYSKDLLPLIYADDVDVRAMLTDVIFLANKLIISEYNIRAARDQDPRLPGRLLVRDGSTISQGEIRGRI<br>REILVGAEIAGQYRLDTYIMAGGFGHGWRGTDLQTGREVFVKTFKSEEEFGSGAETVVAKMVEELETAERVMKVQRLMKHPNVVSVLKVPRNATIIVPSTRQQGDCFHGIVT<br>EYCDGGELFNYIVLPKESGGLQGCAFNEKQARFLFKQIIDLLLVLHHPKEGEGEPYYHGDIKDQNFVVSGSTLKLIDYGTLSKVSDNVGPVKHMTRSHQQPFH
**6083**	MAVLWHWSATPVITRHDLQLQHPGVVAQARGLQQIGGRHPTRRPVVLAAAQRHITPSEDKGIAPLPPFPDRVQTTWRRLFGVAWAAVAAALLAGITVALNKRTLPRTYCC<br>MVAAAADVESPPSTTRSKKEKRRAEEGPDDPDSEGPAYQFPLPAPTYPNFGWQTEVGPAGGQARLGLLTTPHGAVETPAFIFCATKANIKGLPPDMVRDAGTQIILSNTYHL<br>MLQPGSPLVRKLGGLQTVTGWRGPMLTDSGGYQIFSMGYGSVSSEIKGQRGAAGFSTPTVQRITEEGARFRSYVDGSPQDLTPESSIVIQRELGADLILVLDECTPFHVDKAYT<br>ARAMRRSHRWAVRSLTEFAAHDDGTQALYGIIQGGVHEDLRAESVAFVNRQPFFGTAIGGSLGADRATMHAVVGYTAARCRPDRPIHLLGIGGIRDIFHGVRCGIDTFDCVH<br>PTRLGRHGGALVQARYWDEEQPESRVTREHIHLEKARFRDDIRPIDSTCGCYTCRNFSRGYLHHLLRAQETLGGTLASLHNVYFVNRMMAAIRVAIRNGTLAQEEAVWVHPR<br>LYDRNPQEQQEEQRQREERQRQRAAQKERRIAEQLARRSPAPAADTHACTDTDGAPGPNPCSPTDDGSEEQG
**6113**	MTKLAVAEAEVKRLSLENGSLKELLGQQTATKTFKQGYGLWKEVLDGFAAYEKMWKAWGGQPVPLDAFLMQKKTHAFIPVKLNPPPLCDRCSQRPGAYGCPLGLFILECAER<br>HFVCEQCYKAGYTVHQGIIGGKSREYVLAVLRAAFKQSDADFSGSIDFLEGIHFGYLVASRIPDIPEGRRAVYNVLQTLKVAFDAYAANDRVDLDATLRLFREIFHIDPPNLDAIFA<br>SHTNVKAASFAQFLWITYHVGHPDSKHTKHLLKAEKQLFKREPTVKEYQEADDSDVVKSVPTYDHAKSKILKMLGEGGLSMAWLIDYDGATIVAKVPKPSLGYSEKANMFSAA<br>RLQGTVSHRNVLRVLGVHDSSAWPCILLELAEGGDLAAWQGCEVDRRLQWKALHEVALGLNQLHTSNPPIIHRDLKAPNVFVNKKGTCKVADFDFAAKLEPPLYLTSGIMGT<br>PGFMAPEMLANQFYGVKADVYSFGSLAYEVTHGAVPFGEVLEQYPFMDMEGWFQYISTLTQSGKRPAVDNARVTPGMCRLIVSCWRANPDDRPSMAEVVKKLEEIRSEYS<br>L

**6194**	MGNPYGAVPPGGDFVQYTGPPDPASLRYQDPHAANTGPPLHGYDSVAPEGRTVYISNVPNGITEFQLQDFFSVVGTAVKVKLCGEDFQPTRFAFIEFASVQECHSAISLTGISLCGRPLKISQSKGAIQASTGGFGGSPLLPPTNRTPEEHERMTRTVYVSNLPTSIPEAQIVEFIGLSGRVINYKFCGEETQETRYAFFEFENLQGAAACKAITGTALGGYTIKCADSKGLIHTPPEQAVALAMQSAQAAPALGAQNPLAEVISRTIYIGNIDPVISEAEMRVWFEERCGPSMKTNMLEGTSGGYRYGFVEFVDRQSAVNALQYNGHQLGAGVIKVAPSKGAIQKAANANVTQPAVTVQNSASIVALNNQAYAMALQEENRKYQEYLAKKAAGEDTGRRRRRRSRSRSSSPKRKRSRSGSRGRGRRDRDRDRDRERDGSRGGPPRIRERDRGR
**6216**	LDRLGVVEAAFLRGHVGFADCAWDRIIPSLYIDGASVPTYLPKKTVVDGRQTKSGTRRSSLSLQSEVHVVTEEYFEWVVDDTQLKGDLQIAGMLRRSPLQPFISRAMFLLTAGHVTAAYFGHLKGYKYITESMADPEIEKQVREVMQEIAVIEEKRYGADPTETVTYIDQAVARFKNPHIHDAIARVARDPIRKLAKEERLIKPLLTAIKYGVPNTALCRSVAAALHYVSDSDRESVRLHHLVKQLGVEGALKAVSQVSKDSPTHAQLNEEGTIRRIVEAYGQLAAGEIISPVTPLDTPPLDKNALAGTPIVTSLSKVPKGAVHFGAGKIGRGFVGELLALAGYEVTFADVDKHMVGLLHKLKRYDVLVLGLTSNVVKVQHVDAILSMDDQLLEKLATCELVTTAVGVNILPKVAPTIARGLKLRRERGVEDPLNIVACENAVKATTALKAFVLEKLEPADLEYVSKYVGFADSAVDRIVPSMERVDTFVTDGEEQEVEVVVEEFFEWDVDQTQMKGEGYLDGFRDIDGITLTDNLQAYVERKLFTLNTGHAVTAYLGYLKGYKLIADSINDPEIERVVRGAMEESAGVLIRKYDFDPKKHAEYIERIICRFKNTYLRDDVTRVGRDPLRKLGPTDRLFKPFLGTVEYGLPND
**6309**	MADEEERKRKLVADAAREKQLREEIEAIERDTQEEERRKRLDRKRQMRKSKGQHEEERQEEQRLAAAIAAELKAKAAEIPAGQTEKFTFSNGDFYEGEWQDGEMHGSGKYYYANSGNSYSGRWQHNRKHGFGTFTYRVSGNTYKGEWVDGVKHGQGIFTEASGNQYDGEWKDGVMHGQGTYTYGNGDRYTGNFRDDEFNGFGVFVYSNGDKYEGEWLDGAFHGRGKLTYKESGHCYDGFWVNDQKEGKGKYTSKEFTYEGEWKDGCMSGYGVYRYENGDRYEGQWLNDREHGQGTYTFAEGNKYAGEWRDGVRNGHGVYTHPNGEKYEGDWLNNVKEGKGTYFYASGATYAGDWHEDVMHGRGVYTYSSGKKYDVTFEHGKCVEKVALGHGQPGQTVSPPASVSGTSVSGLAVSPKKNGDMSAWLNSLPIDDDMKDIFRKERIDEKAYMLLNSDDLKELGFPLGPRKLILNDIATKRGEASSRPGTAASRLGVSHNGGPELAAGGGD
**6605**	QIQKKSLGEFLGVQKGTSWASDDEEEFMNAPATTETGARDASDWRAGPSKGDGEHRPFASSGGGGGKGGGVLPDRPPYTLYVGNLAFGTTEEEIAQFFRTSESVRLVVDRATNASKGYGYVTFPSRDDLANALKQDGQQLGGRPVRLDVAADRGGKGGGKGGGGFTDGSGGFGGGGFSGGRDFMGTGQVERPERPEPGQGRVNKPMGGFGGGPSWDKGRDAMGSGVVEGRPAARDRPAQAFSRDTMGSEQGAAPTPFGTKPERRGFGGDPDGWSAGRDAMGTGVVEERKPPPPREDRPPRDAPAFSRDTMGASEGTKEGEPRPARAAPNRLMFGASDAAPSRDLFGAEEAPERPERVERPERPPRPEPWRPGGKGAAAPAADPSGGSNWRAGPSAPHASGAGAGQDSPAKQLSEPKPYQPKNKQTSQELEVKPKEKPAPKVPLTQEELFQQQVAKTQKKEQQAKAAKEKTSTQANFNPWEALGGKKKKKKGKKEEAEDADDEEEED
**6632**	YDILSLESARCVEDETRAPLKRCLNGGPDRPEKGSLMIWKDCGEFVRTGHVAVVVNVTDTFVDIVEQNVDDEVWPEGVNYSRRLPAVSDPATGSYTVSCTFPDSEVLGWVTLAEGPVHAGRPADVTRADLEMHVAHLDHPEDNTWLDPTDRKQAAFIEAYGDRLSSSTTAEYLTLTHKGLQGLQQATRELHTMFRFAMDHVIHSMQDTKLTSAFGIPPGLWHKIRRSWFRNNQDTVCGRFDFCLTDTGVKVYEYNADSASALFECGYVQDRWAQAAGLGEVGTGSGACVAEELEKTWRRLRVAGVLHLWHDADKEEEYHTLYMKSIAEAAGISCKIVSGSSVFTWSAAGDILDADGLVVRNVWKTWSWSTALAELEASPTAADDGWAMDFELKDIRDRPDVVARVGGKPARLFEVLLHPAVRVFEPLWTVVMSSKALLPVLWQLYPDHPYLLPSTFERTADMLEHGYVAKPVLGRCGQNVLLFDKGGQLLEEKDGRFGSDTFVYQGLCLLPRFGRRAVQINTFVTRGDYLGPVVRSEEDNIMQSDSAVLCLRLLP
**6676**	EYAKFLELHGPQLFGSGVPEGLWPSVHAKLASSILDAAEYVQYAEDDDGSLVTVAKQSMTAGSVVFLIDHAWTFKLDAAKPQLRSVAGLLERVEGLVQASPAPEAALEERVTAAWERLWRYVQTYRVATEEQMDPEAVWYLDDELGCRFDHDSDPNFQFAPLFYAAEQAAYTLAWPVKDVEEGELLSRDMLRGADAASGQRRVRELVWLDDEEALLECREAYRRMAAEEGPTFHVPATSGPQAGAQPLPDAERYWIYTDNPVLRRAALPPRFEFCDDLSLAHVVWCDYSRLDLGTLRSEQIVNQFPLDECLSAKEQLSYTVRAKWGRPPWLPVTYDATAELAPFVGDYVARREAEEDNAWIVKPHRMARSMDVYVTSSMDCLLRLSETGPRVICKYIHQPVTINRKKFSVRFTVIVKSFSPLEAYVYEKFTLSCSPNDFSLDHLDDYKTHTTVMLYRQEADRECETKLKCDDFVEAFTAEYGEGQWAAARSAAEDVLRQALEAGAHRGMHHPAGRALYGADVMWAQPGLQPRLLELTYAPDCHRIAERCPSFYADVLRTLLLGEPTNVRPLHGPPAAP
**6882**	MPPTPGPLHADGRLLSSVALVAGLCVVAVACAAFAAPTAQLLALPLHHSRPVVAAAPPLSLSAADRPTRQSVLLSSLRPLVPGTDFPAPDTATSQAPQEVVLSWLWPLAAPAALLAAALLGWAQRERRRLGAAVPLVQVALADNLQAQPWRMAAAAEKIRKPLTKDPYADDVRLAFMGWLEQQGLPEQPVSLKYVGKAGWGLVADKPLQRGDVAIRVPKSLLITKETATAGLPPPIAELIRAKNLPDFWVLAIFLVENYAATEQKEPCPYAPYILSLPRETGSVLEWGRSSITDLLKGSPVESTAYELNNDVNDAIAWLSREMPGISPRYGKFYTRENLRWAFAILFSRVVRLTAMGSTLALIPWADMLNHSPTVSTHIDWDEAEQAVTFRVDQRYELGEEVFTSYGPRTSGDLLLTYGFVPRNRRLQSGNEKVELKLQVDPSDPYAGPKTQMLVKYGLQTPATFPLQLNAYPKNIVPYATFLAFEPKSEKDVEEMAEKLLGPNPKRVPEEVEALGRKLVKQTCFDVLSRFKGTAEDDKKVLQPQLEKQAGIEDFMAQDSRAAAAAVVRLRERAILKKTAESIESPGDWLTDLMNTNPMDFFENVFKRR
**7369**	MFLSNRGTEQLTRKEIKQAEKSFFTQYNRSLYNRGPGVSPATPYGAAPYPSPYAAPYPAPYAAPYPPPYAAPYAAPAPYAAPYGAPAPYASPYAIQPRIPGPVPVLAPPPAPAPFAPLAYPATAPAYATTAPAAYAPYPVARPPVVAPVASVTRTYVHPPTTYAEPYAATTTYAPASVVRPAPVTRPAGAHFKSYEEAYDYAMGGRRPAGSAGPTIVRNYDHAPTYHAPTYRSSHDYPHSSSYHAPTYGYHNGDEVIDIQYNHLPSGSHPPPTSSYARSYRRGDEVVDVHYR
**7397**	MRAGPSAVAGAWLAVGCCLLLGGGGAAQGSLWTTSVARSPAVTVRPIGATDERLGRPLAHQRSGAAAVLPHRVGTSGGWSAQAAPTTALRTAVLLVAGLLSAVAIALLGRGGGRPLSDGQSFALMANTGGSKENPPRPTVVISRCSVYVTEGDLQRQFPGAVTVRMERLGGAQGARAVLRFRTAQEAQAVVKTPGLQVNGTPVVARIRAASAKKAKAEAATRTVILLGCPLAATKDDLRQLLPLAEDLRPVVVAGEFTGKCFAVLPTREEAEAVAKARGLRLRGQPVRAVMAAEGLIEGMEANRTVVLAGCPESTKATQLQDQFLGVETVRLLPRWPGGPFSGVAFLVFPTPDEAAAVARASALQVNGAPVKASLSGDKRWLQAQRPRQVFLSGCPLATTPAELAERFPTAEFIRPLPTGGPFSGRCFIIFPTAEEAQAVAGTAGLEVKGKPVKARMGTDGEAILQEEIARTVRLSDCPKSTTPAALQEQFPTAEGVFLSTLRGRASVRAFLVFSTAAEAAAVANTVGLQVGGQPVRASMTDPTSETKTRESPAGATP
**7645**	GSHQGYKGGGQNFAAMKEDDAEVQQLFVDKLQAGINFDKYDNIPVEVSPADPSTPPIAMFQHLQSLHASVMANIQRAGYQRPTPVQKYGLPVICAGKDLMACAQTGSGKTATFLIPCINRSLWEGVPQHNMHGPAHLRKAYPSSMILSPTRELATQIFDETRKFIFQTNLHAAVVYGGADYKSQFRQLEAGADVLVATPGRLMDMMERDKISLSNVRTAVLDEADRMLDMGFEKDIRQILTKTDIPRTGHRQTLMFSATFPKAIQQLAADFLSNYVFLTVGRVGSTTDMITQRLLWVEDSRKKSFLLGMMLHHDAGSTLVFVNHKQEAQSLGHFLKQHGVSANSIHGDKTQEEREQALWAFKSGQISVLVATDVAARGLDIPNVAHVIQYDIAGSIDDYIHRIGRTGRAGNTGNAISFVNDRNKGICPELVEVLREANQEIPTWLEGIVRHMGAFNVLNKKAGEGNFGGQDIRQDRSYNNYTKQNQAFKAFDEEAYRGFNFEAQPAAAPTPAAKGGPGRGKGLYGKGGAKGAGKGGYAGAQQQQYSLPPDPAVAYASQESYGAPNDKRRRY
**8156**	MPWLRAALVTRTKGWLPCCSPRLSAALFSTAAPRLQRRLPMLTVGSAQTTDGDVLVTAPFDRAPLALVPSADAAAVEQALETASALFRDRRRWLPVPERVRILRALARIMEGRAEDLARQAAQEGGKPLLDSRVEVQRAIDGIDVCIETLRTQAGVGIPMNLNAASADRMALTHHEPIGVVVAVSAFNHPLNLIVHQVIPAVAVGCPVIVKPAEDTPLSCWSLVEMLREAGLPQEYCQALVCRDVANAQRLVCDGRVAFFTFIGSANVGWMLRSRLAPGTRAALEHGGVAPVIVAADADLDSAIPSLAKGGFYHAGQVCVSVQRVFADRAIAEDVARRLAAAAGALRVGDPTAESTEVGPLIRPREVDRVEQWVQEAVAAGAKLLAGGKRISDSCYACTVLYDPPRDCKVSRLEVFGPVVCVYPYDTVDQALEWANAVPYQFQAAVYTRSLETALHCYGRINAQAVMVNDHTAFRVDWMPFGGGQLSGLSVGGLPHTMKDMQIEKLLVVKSPALAH
**8170**	LKEQELLERERQLKQLAEKAAPPEPRAPPVESRSYRSGRERRRTPPDPIPHQPPPYRRPEPQEERRPAPPPAIREPPIPIREPPIPIREPPIPIREPPIPVAPIVSAATILEGRLEALHKRNDSVAMLEAFESAKLVGVQPTAQSYLYLLSTLSMADDASNMETVYLELVLQPALSLKPEQLHESILILSQSPLKGRTMELYSQLRRTGHLIQSPVTYRILLSCCTDAAGAMAIFEDMKKSEMLITLPIYHSILSFLASQPQSIRVVLQDMTDHGVNPSDVLGQMKAPEALAMAQAIVQSGMAVEEPLFLQLLQKVGATDLPGLLAMWRKAEALMTLPCAAGLLSLAVQHGQLAVGLSVWHEMQKHQLPGDAPLAMTLIEGLSVARDTRIFEVYSEAKSRLQPSLALFNYVLVVAAQHRIVHAAKEMWRDMQANDVLPDDEALHFILQAASRDQRALVELFDVIRASQLAIPSASAGILFEAFNRTDEGRARIPDLRRACPELNMTHPLPPGPGVLGAAASSSSSQPLRPPVPPVPRPPPRPYERA
**8266**	AAASSAAGGAAAYPYAGPGMGQRGPGPMGDPAAARNMYDAQGYGQQRPQGGPGGVYPAQPQPPAQDWQYGARGGAAPAQPPAGAPYGGAAPAPYPTNYPDPRGGPTPATAGRPAAPQSQWQTPDAGGYGRAGDDRGGYSAPSAAAYPAQAGGGYGDAGAARYPQGGHRAEEYNSARDPYGASQPRPGYLAAAERQPVVGRVPDPLAAVPDPYDSYRSAERRPPADPYPPSNAAPPSSSRRPEPSNTRAPASTQGHHDSYHSSEQYPNGSSSNNRSGADNRYVGGGSSLYDRRDRHDPPTSSYPEAKRPRLDSDASRSDRDRERERGREREPQYSSRSGAAAPREPRDQDPRDRDPYPSRGQPEYEQSRSTHQSYGGGGAYPDSRQPADRNPSGRSYGQGEPDRHSLSAEVVRDTIDRLRSAPPAPMVYGSGGPPPRRDEDPRASAARAGYAGRADDPRSAAGHEGWTEAAIAKLVQSALQKTAATSPAPRIPDPAEQARASGRPVQYGDASRDSRGDPRGDPRGDQDSRYRRR
**8300**	MAQPDAKRPRPAEAPAAEARRLRESTQWAVINGLVMAVPGATQTVVTHCPYTLRPSPYPRRLFQQALELSQPFNRLVHAVSQDVAFLVDVHKEVVLVDDFTRHLVDILQAVHAEGVAQPRSLGIFRSDYMIDAAGHADCAEWRLRQVEINTIASSFAALSTKVGRLHRYLADRFEGGASEALPESTAGHLVPKALYEAYALNVAVPAEVKAARGVVLFVVQVGERNIADQKALDLALWQQGCLQSLWLSLQEIEELGTLDADRHLHINGKVVVVAYYRAGYTPRDYEAAEGPCWAARLKVERSTAIKCPSIGYHLAGTKKVQQALFDQQQLARFLSPADAALVSECFAPQYALSEDNDDVIQHAAENFRGFVLKPQREGGGNLVADEALQQMLQDITPEVRKAYVLMHRIRPPVLEGAIVRNGQLETGPLISELGIYGLCVGAGTELLYSECGGYLLRSKFERHADGGVAAGVACLDTPLLY
**8521**	AGDAKSTQADKLLPVTLLGDLLDYTPLDKCGEVIELMERSDQQIKKCLDNMPSKLALLAMCKKLTGRLCMSKDTVLHGRVMLLLANFFDMYERSGLNLQGDTNQVETHVEAETDSQDAPGDSANDAATPALAKYDFYALFWGFQDYFTHPNLLPDKLDKFGHELQVILREFDNNLHLDNEEGSSWALYTSSSLGSKADGLLKEGPITSLPKYLTSRRLMSLQLRDGTFRRYVLVQVLVLLHSVRHYRQGKEATAAQLAKVENLQADVVQCLKKIPPNGELFVKCVLQALKRETVWIQWKLDRCPDVKSNKGIEGATLLLEEHLKGKASVPEPKWHRMGNPALSKLWAEHDVPADEMCRLIRRGPTPDQFIKEFKDQLAPENAVEQKYYLSKNRLYVWRALRHISRQSLMTFCKSLAGATDKNAAEMFEKLVRDLDDEARQSGREERDDIQIVTAPSRGSKAGTIRLSSSPVPSRDEPAGKKLRVD
**8554**	MKVKVSIFRFWTNLKIGLALADRKKLALYSGAGLATGLTGVSLLMAHDCESVFYDGILFPLLRRLEPETLHRLCINAAKWGICPFDRSGDLGRMEKALAIGVCGMVMSSPIGLAAGVDSSAEAFDRLFRLGVSHVEVGSVTPDPQAGNPRPRQFLLEPDGAVVYRAGQPNDGLVVTKERLQKRMKKLDGGVVGVNLAPNAQSATVVEDFVRGIQELGPNADYLVLNLTAPEEGPWHEYRFGPKLHALLKAARVARDGLPGDWVPPLMVKVSADLTPREQLQVALAVVNFDIDGIVVGGGTQDRPAGMLSPQQKEAGLLCGRPCTAKTNQCIQNLYRYTRGHVPIIASGGVLSSLDAYNAIRSGACLVQLHSALALQGPGVVPRMRRNLWKRLKEDGFHAVGDAIGADVHFEEPARRPWWRWLW

**8570**	MASKNTYILFELASGFALFHCKEMVQIEQLTNESTVACQDFGRFKSMVGLVSFAPFTTADMAIENINAVVQGQVTPFLSDFLKLQLGGSKKEKAELGVADVQLGQTIKSTMQINCVANDTIFEMSRWIRFHIAKFIPELKEADIQQAQLGLAHSFSRTLVKMNVNRSDNMIIQAISLLDQMDKDINTFAMRVKEWYGWHFPEMKKLIQDNEVYCKVCVVVKDRKNLTDPAVVQKVADIVGDEDNAAEIARTSAISMGMDFTEADFLNIETFASRVVNLTAFRKSVFTYLTEKMNVVAPNLAALMGEQIGGRLICQAGSLTNLAKYPASTVQILGAEKALFRALKNKTNTPKYGLIFHSPYIGKAPKEHKGRISRYLANKASIASRLDRFMDEPNRVFGQILAQQVEDRLVHLETGKEVETNEQVMEKAMVAYADSLGKKLKKKRKAEEAGVAEEADDGKKRRKEEKKAKKAAAAEAAANGAAAEAAAGKKEKKKKAKGE
**8816**	KVSKGKNPVWKEAVRGVRIPAGVQYITINVWDEDPGKDDFMGTHRLLLSPPPDGDTWLPLHARENEPKDAKLAKKGNLGVINLRVKCRQGRLDSAYVPPPSPSPTPSKAGVATLDLEVIEGRDLVDLDIIGKSDPYVQFCWGTDPKPFYTTKVSKGKNPVWREGVKGLRIPDGTQFISLLVWDEDPGKDDFMGVHRLRLVPPPNGDMWLKLYPRENEPQDAKLVKKGNLGEINIRVKCTGVRRGSGPLPMPEPLSLTGPVPTRCRVTLIGAEGLAPADSNGLSDPYATISVGYEIASKEHKTSAKKKTLNPIWNETFDLHVPENETHLTFNVWDDDKLSKDDFLGQAGLDVRGPMRQNGAFFLPLRPRANNADDQKLVSEHGGLGKIRFKTEWVGLPTTVPASPTFQTASSQTSVPPSPAHQPAPIPRPPSEVVAPPPAPKPAPPPAPKPAPPSPPKPVEPPAQTFEEVSVW
**8825**	MAWPAGRFAPLARALRRYSTTTAPLSEKQAQFLADFRQRLAAAAHEEGGSGVSFEEKYPLRPTDGPSLRRQLRDQRLPMPSWLQKKKPGGDNYGRIKAQLKDKKIATICQEGRCPNIGECWGGGEGAVATATIMLLGDTCTRACRFCSVKTSRAPPPPNPNEPEETAQAIASWGVDYIVLTMVDRDDLADGGSAHVTETIQAIKRATEGRVLVECLSGDYAGNRDSIARVARSGLDVYAHNIETVERLTPQVRDRRAAYRQSLATLEYAKAVVPGLVTKSSIMLGVGENDDDIRQAMRDLRAAGVDCLTLGQYLQPDRQRMKVARYVPPEEFDAWRAEGEGLGFKYVASGPFVRSSYRAGEFFIKNIVNSARAVSSS
**9045**	LDLGGAAPLGTRLPVQARTPPVLAALPPTPWLPSVGAALPQLQSSGPLLTHPDLARPAPTPGPGWAALGTAIGAMAALLLAVVGHAQRGPELERSPSVVALLAVTGSASAPAVEPPEAHHIPPFLNKTPHSADIRLHFYDDALQAVQRAISAGESRMEVRTTFPELNSETDVYRAATMLEMVRHIATALATEGKRVKICVQQSLGQGIFSGLPLSLNGMMKLAKLMDWDEEAVGDRFSFGEVGAGQVESTVDVYLLISPQNITGCSIVELLQGMCKAANGKPIVLINPKLDDIPSSGGVMSYRGREERMEFVATFRTVYHFRLLYRKPYLYPIYGALRMAYGGPWAVYKRIETGAAPDLQEEYRFSRSFPSEPDSGKIMDAVFGSSSSAVYLS
**9049**	LDLGGAAPLGTRLPVQARTPPVLAALPPTPWLPSVGAALPQLQSSGPLLTHPDLARPAPTPGPGWAALGTAIGAMAALLLAVVGHAQRGPELERSPSVVALLAVTGSASAPAVEPPEAHHIPPFLNKTPHSADIRLHFYDDALQAVQRAISAGESRMEVRTTFPELNSETDVYRAATMLEMVRHIATALATEGKRVKICVQQSLGQGIFSGLPLSLNGMMKLAKLMDWDEEAVGDRFSFGEVGAGQVESTVDVYLLISPQNITGCSIVELLQGMCKAANGKPIVLINPKLDDIPSSGGVMSYRGREERMEFVATFRTVYHFRLLYRKPYLYPIYGALRMAYGGPWAVYKRIETGAAPDLQEEYRFSRSFPSEPDSGKIMDAVFGSSSSAVYLS
**9215**	MATIPKLRQYPVRRSATSPSRVERVSYGAAPPSISPARVHVSPSRVSEGRTYVSGPPRDVPYETVVVGGPRRQCVTTAGGLQHTEELVAVPVPTPVEVLHYQVVEVPRTEVVDKPVVAVQERIVEVPTERVVECIVEKVVIRTVEVPVEKIVERIVEVPVDRIVERIVEKVVVVPRDKYVDRIVERIVEVPVEVPFDRIVERIVEVPVEVIVERIVERIVEVPIQVPVDRIVEKIVHTVQEVPVVEVSERVVEVPREHLTDKVVQVPIERMVPVVQDKVVPHNVERVVECEVPFDRIVEVPVEREVIREVEVERPIVEEILRPVIREVEHPVVREVTRPIVREVQRPIVREVERPIIHEVQRAVPVVQDVRVERSYVPCMTRTSIAPPVTVTEHPPVVRRSMGAHSSSAAYVHAETRRSLSPARVVEYAPARAARGSAYDTARALDAADGVIDGKYFGRDIRVVKELR
**9484**	MTKQERRALQEQQRAAKQAKKDAEVQAAPAAKPTKAPASAPPSQAVASVSPAGPAAPSREGAKPAASAKGAPAKAAPAPAGPKQATPAPAQGPHQPRQPGTKEAHKAAKRGRVDLTDLHPAILRLGIQYQTHEIRGGNARCIAMLQALKQVIQDYTPTQTEMKPNHGRDVVQALNKPIQYLINCRKLSVSMGNAIAGLKHKITTTCDAAKDISLESLKKIVGQYIDMFIYERIVDADRFITSHGVSKVAEGDAILTFGRSSAVEMILRTAKQEGKKFTVCVMDSGPAFEGKGFAQRLALHGISCTYVLLSALSNVIEEVTKVFIGAAGVLTNGSVYSRIGTAMVCLMASHYQKPVLCCCETYKFTDKAWFNSLTSNEEGDPDDLMVKDDANGVSQLGDWRKQENLSLINMQYDLTPPEYITMLITEVGPSAGPGRGPQEMMSGGSTPTLCLAVPPKTSCCIHLWDHAYPFGFHNRPFVPHPRGAICLPGTASDDPADLCARDHPRVPPESERFLAGPSWQALACLYHTPTW
**10009**	FNVSEPEKKERSGVLDLSYWTYHIYTKTNLSNFVQKELVCVRRYNDFVWVREQLVELYPGVIVPPIPEKSVKGVIEKLSMISAEGLLQYRQRALRKFLVRVGAHPVLCTSDLLREFLELREEDFNRRKALPKRPAAVPIGQKFRELSFSMAKSFGGSAASPATSSTPAAVAASAQPLPAEEALPAGAPGARWEETRHYIDQLERSLCLLKERIELLVKRRRETSLSLLEFGKSFVRVGEIEKTYEEGTLPQALIDVGHHSEHLSIVYQEQAENETVQVVETIIYYCGLTASVRDCIKRIQKMALTHDTIQDSLTKLQDRRTRLQGKDEEQRRLDLEISNTSQRNDELAHEIAHAEATFAEELRRFHREKQYDIKQMLRAFVDLQTEYFGKMKASWDAVAPSVEAIKTD
**10196**	MSMDPALPGRKKMDPAAPHKFWDTQPVPRLVESPDAITNAGEGEINSDHKVEDVQKEPYKIPDAYEWFNPDVHDDGDVTAIYELLAGHYVEDDDSVFRFAYPKEFLVWALTPPGWLRDWHIGIRVRASGKMVAFISGIPAQLRLREKVVTVTEINFLCVHKQIRSKRLAPVLIKEVTRRVNLQNIWQAVYTAGIVLPRPVSSCQYWHRSLNPQKLISIGFSRVPYQFEKFQRPMEMTKRHFALPDKTALPLRPMKEEDVPKVLALLSEYLKQFLFAPHFEEQDIRHWFLPRDGVINSYVAEKDGAITDFVSFYTLPSTVIGNTKYSLLKAAYSYYNVATTTDIVKLMNDALILARNLDFDVFNALDLMENGKFVRELKFGIGDGHLQYYLFNYRFPDVEPKDMALVLL
**10462**	MAGQGTIGLEMLAVQPDLDCLLVAVGGGGLISGVATAVKALKPGLEVIGVQSSKFPAMVNAVKGTQYPQGQTTIAEGIAVGVPGLLTQRVIKEKVDDFLIVDEGDIEHAIVMLLEIEKTLVEGAGAVGLAALLKYPDRFADKKVGLILCGGNIDPLVLAAIIERGMVRAGRLTRMWVNARDVPGSLARITQVVAEAGANVDEVHHHRAFTMQSVQLVDIELVLQTRGRAHIARVIEALRKAGYEATEQQHFQPLRSPALRPSPAARLPAPL
**10778**	MATGLDKKSISDLDLKGKRVLMRVDFNVPQDKKTGVITNPQRIVGALPTIQLALDGGAKAVVLMSHLGRPDGVPNPKYTLKPVAEKLQELLGRPVTFLSDCVGPEVEAACAAPADGSVILLENLRFHVEEEGKGVKDGGPFKATPEAVEQFQASLARLGDVYVNDAFGTAHRAHSSMVGLRGKTACVAGLLLQRELEAFCKVLDPAEVKRPLLAVIGGAKVSDKILLIENLIEKADALIICGGMAYTFMKTNHDMPIGKSLYDAKGAAIVPTIMAKAQAKGLDVVLPVDWACGQDFCNDQEIKIVTKEEGIPDGWEGMDCGPASMALFREKILACRTVVWNGPAGVFEFDNFSKGTKAVLDAVAELTKAGHVGIIGGGDSATAAAKFGMEDQVSFVSTGGGASLELLEGKVLPGIAALDDK
**11076**	MLATSGEKRPTWFPGAEAPAWLTGEFPGDRGFDPCGLARDPVDFAKFRDSEVFHGRWAMLGLVGCLVPEVFGNLGIAQLPAWYDAATVANTSNLDYLGNPNLVHASNVSFIALSTLLLMGPVEAWRWNGALASEAKSAERTTYPGGPFDPLKLGASPELKLKEIKNGRLAMVGMFGFWAQSYVTGEGPLANLAAHLADPAHNNLLNTVAMFAASGEKRPTWFPGAEAPAWLTGEYPGDRGFDPFGLAKDPEDFAKNRDSEVYHGRWAMLGLVGCLVPEVFGNLGIAQLPAWYEAGAVANTGSLDYLGNPNLVHASNVPLIFFTTLLLFLPIEAWRWNGQIAPDAKSKEWTTYPGGPFDPLKLGASPELKLKEIKNGRLAMVGMFGFWAQSYVTGEGPLANLAA
**11216**	MDSYPSPSTSDDLAGPRRSASISHVSVDLSRGVSEMVPRPAVVSTEYRAASGPERGGSFLSSAHPLPEASAGLEFGRNTSWRWMQGYGAGKAPALSAQEAAVIRQLVVDGNALPNQHGPHADIDGRLSIPTSLEERYQAPLTEAEVQRLRASYAARGGEVRQESHSDPDSAPGSAAGFDRRSSAPVPPRPVLPPPRLVSSGADRPPPVPPNSVQSPKVGSIRTRVPGFDMSPTTAIATSVLDGSFAPTRANTSVGSGAPAGAYRAVSSGYFAHPPPRIVSVVSEYSGGSLPSRNVSEATSPLHGGSALHTRVAAGGPPSFSEPVGIGGGQWGGVGAVEVLVRPSSLQAPLPYQPARLSPDGAGGGPPAPLDHVFTSFGYPSTPRVSRGAAPLPYDVLPSPANSRARVSRTYATELAGHRRKQSEGNA
**11395**	MLPDDANPAGNVHGGTTLRMIEQAGFVVATRLCNRPTDKPHPPLMGSLVQIYQMSFIQPMHVGDLARLTATPTFTSNRSIEVQVDVWADNLMTGEVRHTNHAVLNYAAVDAATGATIDFVIPQLVPTTPEEQALFDAGRERYQQRLTSQQKVNPSPVSPSPTPVIVPGPQPTGDSAISLVQMMLPGDCHHSNVVGGGVILKLMDNAAAVCAVKHCQSNVVTVSIDTLVFLSRLRIGDVAYAKARVVFTSARTMDIQVEVEVERFGVEHPIRTTQGVFAFASLDPTGRPQAIPPLRPQTSEAVALFEVRQQKYEERKRAREKSKAAPAQAEPTGPPAAAAAPSP
**11747**	MPSRKKQQVESDDDDMDDHEDGPDIDVKNELRRLIDPDYAEKFVASLDAVVQGRVRALQGMQTTMNDLHNKYMEEHKALEKKYETLYGPIFVKRAEILSGQREPTQEEVTKGQGEDFKEPEDANQEVSDAKGVPDFWLTALKNHDAVCEIIEERDEECLKSLVNITAKTFDDPDTGFVLEFHFAENPFFTNTVLTKTYHLVDEDEIVMDKAEGCEINWKPGQNLTVAIKKKKVKGRGKGTKQITKEEPCESFFNFFKPPQLPDDMDEEDEDDELGDTLEELIEQDYEIGCAIKDQVVAKAVSWYTGEAADHDDDDDDDDEDGDEDDDDDDDEDDEPPPRRGGRKPKEAPKKANPNAQPECKQQ
**12086**	MAAVRERAEYLDVMEEMKYFSERPMTPISHAVFHRFALHQTQKAALLQGALFLKAELPIRLAHSAHLPDRLPRGLSGLAAFQQVREMFTDSFRELRAFQFSKPAPSWEDGQRFHDMLSVIQSRHKRVPEIVFQGCQQLRTQLQRTHGANWADHPDRQLTQDVLEDFFGARVVVRFLIGQQLRLHAQYVRGESHADVFGLIQLKVDSEHLMKASIATAKRMALASFNVCPDIELTCKCGTPFIQIGYHIQFITVRLLRHAIMGTLKAHGVEKVKAGKAPPVTITVADAGANEELCIRVQDSSLGVSRSKLRYLWSYLHDDVRLGEQEWVAATPEDQQYGESFGLPLARVRARLFGGDVVVQSVEGLGLDMYAYIPKFKAEEVLARRPFPN
**12275**	MSPAPAALPAALCGAAAGLLVLIAAAAAFSPASPPPPAALYRAAVPVRVGFAVRPIQAVSHARQGRLGAPGSAQLEFGAASESPASHVAGYVGRTLFAMGAALAAASALVLWLFREEEQPWVMAAAGKGFGPPKVVKPKPVKKPEEEEPCPCCSGTAYGLCCQSYHQGKAVPATPEALLRSRYAAFAKKKRDFIRTTTDPEHLTRNNVTETEHMESVTLSCNNVEYSQLVILKQEPGETEDDWWITFRYWFGFTAEFKGRHKGVTRQSVEKGETETRTERSLFRRTDGVWRFVDSPDEFCNSDSFEVGDTSATQNLLLKAKDAAANVKQLAAQAVLPKMEVRLEEQ
**12357**	KVHISLVVIGHVDSGKSTTTGHLIYKCGGIDKRTIEKFEKEASEMGKGSFKYAWVLDKLKAERERGITIDIALWKFETAKSVFTIIDAPGHRDFIKNMITGTSQADAAVLVIDSTTGGFEAGISKDGQTREHALLAYTLGVKQMIVATNKFDDKTVKYSQARYEEIKKEVSGYLKKVGYNPEKVPFIPISGWNGDNMIEASENMGWYKGLTLIGALDNLEPPKRPSDKPLRLPLQDVYKIGGIGTVPVGRVETGVLKPGDVVTFAPNNLTTEVKSVEMHHEALTEAVPGDNVGFNVKNVSVKDIRRGYVASNAKNDPAKEAADFTAQVIILNHPGQIGNGYAPVLDCHTCHIACKFATIQTKIDRRSGKELEAEPKFIKSGDAAIVLMKPQKPMCVESFTDYPPLGRFAVRDMRQTVAVGVIKSVNKKENTGKVTKAAQKKK
**12528**	MSSWQEQFDVTCAEIKKIYTEAIEPVEKRFQYELFKPSWFADTLKPSKPFVLFLGPFSAGKSTFINYLLGHDYLWTGPQPTTDKFTVLMHGPEQNTVSGRILCSNADMPFRGLAEFGNQFLETFSGMQVPSDLLRNVTLIDTPGVLESAQEVHTRTYDYIKVARWFVEMADLVFVMFDPSKLDAGVELRNLFNQLKGHESKLRMVLNKADLCEPQELMRVYGSLFWSLSNLIHTTEPPRVYVSSFWNKPYRTPQNKELFDEEKADLMFDLLEVVPLQCLDKRVTAVMRRALDVQIHAHVVGTMKERMPSMMGKSSAKQKMLANMDPIYDEVATKYKLTRADFPDPKLYSAFFDKPQVHMSEMPKLTDFEKEAKKGAENPLKRLERIIRFDLPKLLHPLAQAAAVDPRKARKERQGQGNAPPDKAVFLDQLQEKQQPKARYE
**12831**	PQLPAHHLQAPLNGHVPSIPLSVEAGAVKGRLALVLAGSACAAIATAVLLGRRGPTPGLRRSTALRLVEPGSPLVALAPSSPGWRRPMAVAAEPSPLLRDTEDLQLAVLMELELLRERQAAITEYLQAPHEDAELDVGLVKLRVKQEIEEWRQREKAIEDVFDLQHKAHPSKGVEGARVRAAVELEAMRRTEDALEAFLARPAEVEEVRGRVLADLWSLRQRQRTIEE

	AVKELPKSKSSDVAKARILAELEELRSHEAALEAFLSPEARAGKKAKKGGEDAMRVRVLESLLALRQREDALEASASAPLEDVPAEALPELQAGLASEVEALKWQEAALEDYVRAHLKE
**13018**	MAPPLFPNVICCIHILLLCTITCALPLATVRTGTECWREWMQKERRYQRAKRLRMKQPCEDGGKSYDTNIPEWFCEDTRRLPPEMTGDGPKWACGMETFKKHPKVLLVSMGCWGEIDWERAVRGWLPQAEMHLIDPSGHDGGWDLPKTLEQLKPLNVTFHEFWIGSTHVPGHKFPVLSVVEAMKRLGHAGETIDILKVDIEGHEWKALPEPIASGALKFHHFNVEVHNMNCREHIPFFAALDKQDYRMFYKEPNHWGCGGSGCIEYSFVSKEHACAEFATAHNCPPCSQWLNGPRSDAKPDA
**13288**	LYVGWFGVLMIPTLLTAATVFIIAFIAAPPVDIDGIREPVSGSLFYGNNIITGAVVPTSNAIGLHFYPIWEATSLDEWLYNGGPYQLIVCHFFIGICSYMGREWELSFRLGMRPWIAVAYSAPVAAASAVFIVYPLGQGSFSDGMPLGISGTFNFMIVFQAEHNILMHPFHMLGVAGVFGGSLFSAMHGSLVTSSLLRETTENESINVGYKFGQEEETYNIIAAHAYFGRLIFQYASFNNSRSLHFFLAVWPVVGIWFTALGVSTMAFNLNGFNFNQSVIDSQGRVINTWADIINRANLGMEVMVRQV*
**13428**	MQQTVLRLSNGVDMPAIALGTWKSPPGLTGAAVKAAVRAGYRNIDCANDYGNEAEIGAALAELFAAGEVKREELFIQCKLWNSNHRKEHVRADLLASLKDLQLQYIDSFVIHWPQACPSTGKAPAVCKDGPHPGPKDSGCMFPLEADGTYASDNACHYVETWHAMEELVDEGLCRSIGLSNFNRRQIREILEVAKKHRPVILQNEGHPYLQQKDLLDYCRREGIVFQAYSPLGSFDRPWAKAGSITSGPPSTGHELLTDPVLVAIAEKHGKTPAQIVLRWHVQRGAAAAPKSVTPGRIVENLAIFDFELPAAAMDAIGLLNCGWRHLVWAETAMHPDYPFKDELPHGYIPGPAPTSTTSSGQ
**13576**	LDESPNNVKLLFEQGQAYFRLEEFETSVNVFQKGLQVAGASTLPEDKELVKKLELWVRKCNANLSEDKQVHIDVKPEAALAQAQPPTPAPAPVAPARAPPEDQSMAKVRFEWFQSLTHVTVTFFCKDRTEKDVDVEYGANSLTVSIKLGDGKEYQHSFSPLYSEVVVAESSHSVGKYKLEIRLKKAKDIQWRAVDRPAGEAPAPEVTASSAAAPARPAYPSSRPVKKDWDKVTADIAAEEANEKEEGDAALNKLFRDIYSRADENTRRAMNKSFVESGGTVLSTNWEEVGAKTVEGTPPKGMEFKKLGE
**13640**	HVAHAGLIVFWAGAMNLFEVAHFVPQKPMYEQGLILLPHLATLGFGVGFDGIVLDTYPFFVCGVLHLISSAVLGFGGVFHSLAGPDTLEESFRFFGYTWKRKKKLAAILGIHLCFLGLGALLLAWKAMYGGGVYDTWCPGGGDVRSITNPTLNPFIIFGYLVKSPFGGEGWIVSVDSMEDVIGGHIWIGILLVSGGFWHICSRPSPWVVRTFVWSGEAYLSYSLGAVATMGFIAVPMVWFNNTVYPSEFYGPTGPEASQAQAFTFLIRDQRLGTNIASAQGPTGLGKYLMKSPTGEIIFGGETMRFWDFRGPWLEPLRGPNGLDLNKLKNDVQPWQERRAAEYMTHAPLGSLNSVGGVATEINAVNFVNPRSWLATSHFVLAFFFFVGHLWHAGRARAAAIGFEKGIDRSREIARKLKPLD*
**14460**	GLDVPTVTLPSKHQRMRSRLPLREEVAVGVAPATTPEVGLEAATGAKPRAWPRWPHALGLTAAVAALLATFSVSRHRLARAAAAAPFVQQAEDAPWCVVPLTGEAFDPLGLNEDGEPAGRSFKTRYGQALLFAWVATFSAFPASAADLDVFAGLDESQKTFVNFLIFGQAVGFLGLLATANVVRQQKEELDVLEDTLEKVYRQLRQRARARQGELLAQVPPVLLSPVLDQDDHEEELQRAVVQLLRHGRHALKEQHGAEALREFQAAEQLLRGGGHLEDRLRFERKAERGVAAALQQLGRTEEALASLQRVLDMTKTIGDSTGLEEVYGAMADLLAELGRYVEAGKYYDLHSNRVVEDSVTGVEGAL
**14530**	MVHIDNDVKLDFKDVLIRPKRSTLRSRSDVDIERSLIFKHTGKPWTGVPVMVANMDTTGTFEMVQALARHRMFVVVHKHYTTAEWTDFLHAHPECLPYLAASSGTSEADIGRLREICAIEVPSPEGTLGVQYVCLDVANGYSEHFVECVRRVRHELPHVGIIAGNVVTGEMTEELILSGADVVKVGIGPGSVCTTRKQTGVGYPQLSAVIECADAAHGLGGLIVSDGGCTCPGDFAKAFGAGADFVMSGGMFSGHDESGGELIERNGRKFKLFYGMSSETAMKKHAGGVAEYRASEGKTVEVPYKGPVESTALDIAGGLRSACTYVGAGKLKELSRRTTFIRCTQQLNPVYAANLEYAPQPRTVGKP
**14553**	MDGDRVVLPGPRPGGSPTASPGRLAGMAVLLAGVGFFGTRSALSGSAAQLGHVPTAPVAALTPAVATGTVAVASMAAPAHTLAAEGGPHVLPAVASRAAALADAAAVAAPDALQLALGLAAAVALGFVAWLLISRDAYADQSKTPIDVEVPPEQEIEVSPSGIAKVGGYIATSPKGWKLQADTLKARGVRSVSGAEVVRLAQGKNVAIVDVRLRYRFEQFSIPGSVNVPLFQPIDGWAPFKTLRRIQFALFAVEGTECNPTFMQELLAKVPRNKELIFVDDSIVATLQPTKNCPDGKAGHAYMAMYRALNSGFRNKMRYLEGGIIEYEKAGGEYVVA
**15305**	VSQIPPLDWKNPEIVASFEAAVKHCSNPDLLDIARVRTKISQIISNTLIGKGFVHPPVQLFSPCSDPLNHETEVASMSYYGQTVTLSQSMIFCKMLVLALSNIDKVFWVSPNVRKELNVKTAGRYATEFTQIDFESSLLDFESCLNLIEEVITNVVNTLADEEGDTIQRLSGRRLQKLTEPMKRFDLGAEAEKLGISQGQAEDKLIAENTVHPFFLTNLKREAYDRRDDETGKYKNYDVLFPVVGEILSGGEREFTEERLTLRMHELGYNLEYFEPVLRVAREHGLKSSAGAGFGIERLTRAILLLPDIQSVYPFPHAGLNVEFQPSLGGGSPCHDRGWEVVTGFQIATG
**15630**	VNGVSYVTRTLNQHIPQYCGSCWAHGALSSLADRIKIRRKAKGVDVTLAIQFILNCGTELAGSCRLGGNHLQTFEFITKTGYVPYETCQQYAACSRDSTEGSCGTGNWECSPLNTCKTCNTFADAGGKCVSIDIFPNATISEYGPVAGADSMMAEIYHRGPIACALNAEPLVAYHGGVFDDPLAPKELNHIVSLTGWGYDAKTGRRYWIVRNSWGEYWGEMGYARVTMGGNQLGIETDCAWAVPATWTEVNYPCDEDGGNCHRQ
**15795**	MVDLGQHGDPLPVALSEPAHELSRRRLLQWPAALLAAHALPLGAGAAEEATPAAAARPPQVFFDLTLDGEPEGRIVVEVFPDVGLGAQRFLDLAANTGGVGYRRSKVDFIANSFVRVGEVPQLSISEAERSPIAGGASTAKLEAALGTAARRHDGPGVVSLLVRSSTPQVFQEKLVARNGQMVTVRKPVGPAPPNGTAFCITTEAAPGLDATNLVVGRVVAGLDLVERLAKLPSVADNHGSPFFILAKAVGDKRADVAERGFGRPFAKVVVTRSGVL
**15813**	MKSLTAGSSLPKKSEEVTAMCWTEATGESFNVRAVGYKTKNLKQPSGPSLYDTVAVDAYSSPKKVPHFAQYVDIPEPEDGHGVPYNLVVNLMIPRYAPNLVWAAAGDGESWCVVLYCRLSEAAREMLRTNQPSPALKLWKRFVEADRNDPMKQRLKCIVRALNPTEVGFSTATRSLINKYNAKPFMIRTTSSFYTGDNYYEIDIDIHNFGKTARIGLYGCSEVATQADFDLSATIQGETDDELPEQILFGVRLDKLNPAVVNVVPFDIASPGNRTPPRQASPRPAEAVPDTEALPDTEAEDDTVHPDEE
**16000**	TTACTTAEAPAPHIRAALANIHPDVLAKYYGCGLVVPHCVQGLRVLDLGCGSGRDVYLLSALVGPEGSVVGVDMTAEQLEVAQKYIAWHTEKYGYPKPNVEFKHGYIEKLDFPDNSFDLIVSNCVVNLCPDKEPVLREAHRVLRPGGELYFSDMYASRRVPPALVADPVLFGEGMGGAQYWNDFVRLAQRCGFPDPRLVKDRHIAIKNTAIKAVVGHIDYYSATYRLWKLPELEPDCEDYGQAVVYKGTVPESPEVFVLDGHHAMERGKAFPVCGNTFAMLQQTRFAPHFDFIGDRSRHLGIFAGCGRALPFASACAPLAKGKGGSKASCC
**16166**	MVAIVFLSASERTTLFSQTALGSTVVPSAVVANPHMWTLSQTERFHVAKGAATPIKEVGKGNLMSSSFAFWAATLTSFTTAFFLVFSRHRNVSLAAASGQKDEEIGLSRREALLGLSAAGALATSAPAYAFLGLGEDAGYPGDTQNIIAEIKSVLVLAADDPTKPDRITTLRRDMNSYVAKYRRDTRYGGRASFGNVYSVINAVAGHYNTFGVKTPIPKKRATQIVSEIGTAEQLLAKGR
**17018**	MFVARTASLFALRSAALRGCAVRSFNTPLQQGQQSLKDAILGIELAGEEFYRKLAMEAPEPLDEVFGRLADEEHIHHRTLEQAFANGTLPDLQGSDIVEVAHKTLRNIERHHSMSGQMKSMLEVYKMARDMEASNRDKYQSLMEKETNPEAKKIWEYLAAEEQKHFKAMDDMVKFLDKAHVYYYF
**17449**	PELQAVFRAVAQGDLAALAPALATLSSVDEADPLGRTALHVAVLEGQEPALDLILERSADPNRQDEEGNTPLMLAVGLGYEEILKKLLTKGGDIDIPNKQGANCLIKAVQRCNPELIAFLLDKGAHVNHKTLAGKSALVCAVEKSKVDIAKSLLAKGAEANQADELGITALFVAAFRNNADLIRLLLEHGADAKAQSKAGRTPLMMAVDSHLCRVETARMLLEAQADPNAQERLGLSALMLAVDKPAAKDLVACLLEHRADVTLKDKRNRTALDVAKEAEAEDVVLLLTGAQQG
**17548**	MSHQETAALLKSHCTINVSDETSASKSCLLSKIIRATGVACICLGSASIFSGVGCAAGLDVCVFQPTAAPQQLFSVPRSVAIRNSLTGKTFVLSRPSTALKASKVPPAYEKAAKLAITGMLSAAAVANPAIATEQEDRLAELMKAWNEEYGAAYGQWDMSGYRTAIKEAPKVAKEAAPAPDIKISRKATQTLDPIDQVMAEWNKEYGSQYGTWDMSGYRPTKKAAAPAPAPAPVPAPAPAPVAPAPVPAPVVPEPVKPEAPAAVLKEEEPAKVEAKVEPKVEAPVVKEEAPVMKPLVKLEAAPVPVPAPEAPAKAEEAEPSEGSPLALVAGGVLFVAAGVLAATAPTGSEEQKPAAPT
**17576**	MRSSAFPVAAFNYQVPETESTTKVNVAFVPLAFGIGALAAVAASLLQKGKQPLATLATSGTQHIGRNRDSNSGTLEDERRRRQREEERAVQERRQRLQREEQERRQQMLAGRSVHQHQHQHHHAKPPAAPSARVVVPTPPVSPPAPTAATAVTATDAELRRRREDRERREAENAEQARREVQERLARLARLGPPSVQPVVPATPSPAPASPDPVFDRESQMRRDARERAEREKQLQAQREIQERLARLQALSTAPPAPAAPAASSAPAAPAARGGNDWDRRQQERERQEREKKERAQREIEERLARLKALGTAPTPAAPVVRPPAAAP
**17693**	AAPAAALPTFGASSAAAGLGSSAPALGLGVSASAPALGFGVSSSAAGLGFGTSSSAPAIVVPEDPMFKEKTTAEILQHWSEGLVKDVKLFDKLGIQVSGWDDAIVRNGDKIVQLHFDVENLEKQAQELEGKLESIDQQQRELKEALTAIETELDKAGPLALTQPDIERQNTFDLALHMNQHLNQMHRDIQRMVEHLNQSFEKAHTQSVDNPLSTIVKILNVHLLSLQWIDDTAPKLQAAVEGAHGAILRAQQDVVTRR
**18132**	PPFPFPSWLPSGEEELLLVRPLVLGLALQQPSWPPQKQFLLPRRQPRRERERRQRRTRLKTRTKRRWKRKEAKENKAENKDEKKVEKKEEKPKVDYEDKEMDCQDCNQKFTWTAGQQEFFASRSFGEPKRCKECTLAKKGKGGKGKGKGKGGKGDAKSCRNCGGEGHLAKECPKPSSCYNCGGEGHNAKDCPKPKVCRNHKAGNCKHGAECKFSHSE
**18196**	PAATEPLVGASSFPISLSAPISAAAATTTTFTSPAPFISTLPSAPTAFPAATPYPSPLPLTSSYNFPPAAPTAFPATFSTTPFTSPAPLSSVYSFPSAAPSLRPPAGYYAYPSRTPTVSGRVPAAYPVAGTPYRPRTLPQNSPNSVLVSYVYGGPAPKSGPYENFTFSEVATNYRQEHLSPFWQGTAKPFFRFFTESN
**18232**	PYTSPAALPFGYSFPAGASALPTTFPATSVTSFPTTFPTTFPTTFPTAPATTYPATFSTTLPTTTFPTTTFPTTFQTTAAFPSTFPTTTFPTSATTTYTSPASATPGYSFPPAVSSYPVTTLPTSAPSFSPAFYPSAYPSATHSAGYAVPTQARAVYPKPTSVAGGAAYYPAWGGSVYRSRTPYAYARPATAPRPQAPLVAGRSAPNENYTFKEVVNNLYAENIGSTASPWRANARPLYDDFITSFEAKTTWLNKL
**18304**	MAFTAQHMYYDAAIPEAAPQRPHSAVWYAALGFAATLALTTLAGPTAELFRPSPLRSAGMSPTLAAGLPRASMALGVGIDAADFRKGFTVEMEGAPYKVLDFQHSKQARQAAVIRTKLQNMLTGNVLEKTFRSGERLDKAEIEYSEVVFTYKDGDDFCFTNMETFVEERLPGTLLGDKVSYLSEGLGVKVAKFNGKAIDMELPTTLDVEIVDTQPGEQGDRATAGTKPATIAGGKVIQVPLFVNTGDLVRVDTRDDRYITRTK
**18668**	MTRSPYVAEGRSVALALAEARAAAKASEIESLRSLFEADAKAVAAENRIRQVVAEVAKSQTRVIPQPPIRVPVVGRSVPEIATWRKVFDYVDELGAGNVSKHIIQARLAPEIGAFDWEDDHDETPLTFQQFVDLMTSSSVPVEKRFEALSWGRTKCPDKVYPADGGKPFFTREYFVQEAQVPTRVSSVGYGYRW
**19920**	QLYLGQGRVSVDPYLLIQNGELVSVQHTFSSSAGIRVLLIPMLPVAQKTFYDDVLSSVSAENGLVLREGVKAEGVPVGAAAGPASPDSKDGSANGDFDKEFGNSAQKLEWKDYDDGASSSARGSAGGDGLSATGGTVAHQAYFDDLAATTCPTELADLSAEESRWQLSKVFQFPPLSPERRDRLLQVLEQQSQVMQYGAYDPASRGGGRFDARLGSPPYLPDSIALPWNAGSCPAIAEALAAQGYTLVSRTNRVILDRSNFRKLYRLRGLSDASGVNLVLSKLVRI

**20098**	MDRTLKSKRFLCQEHHVKRYGRHLQAVDAAKGQFKCKADSPCDPPSCPDCRAVVPGASAICVSCGKLVRELPPDCRTVIVSHLPVRFRVRHCKHLEGYMEKWGPVRRIVPKKPNRAAVVFKTPEAAQKAVMAKHETKDGTMWATMLKKGSSADTPLAPASQRRRVAKEEAVLRQQTAAEKLLARRKEQKKRARQRRRQVATAAGDAKAAPPKPSKGSSKDPPPTTTPAKGKDKSAERPKKSAQKKKAKAAS
**20283**	MEVQSILERSTQKSVLSKAAIFVACFTFCVAPLLLYFSQGNVQGTYFKVATGENPRYRCHTTEGDFLVELFMDSMPLTSSNFIDLVNSGFYNGIHFHRVIDNFMVQFGCPYARHHRHPWAGTGGPPPSTTFRVGNYKVTRDEQGCIPDEFTSYISNVAGTLAMANKGDPNSGGSQWFINMVNNQHLDWWRHDLSASAHPVFAKVVEGWGVVRRISKTLTDAKHTPIKPIRMISITAAK
**20527**	MSSSVNVKFLPHATTEANIQQLFGACGHVLEVQITDPTAVHAVVTYSSFDSASKAVAAFNHNFILGSHVEVSMGDQHGECKVFVGNLPSTVTQDELQRLFGPHGTVTEVVLLPPRASQSDQRCGFVKFSSYQEANSAIQAINGTDWNGSKMAVRMADAGRKQNVYSALQQILPAVYAATTAANHMYGHGQPMHGMQPVAALTSPGTKLFVGNIPSYWTKDNLAAQFGPYGVLEETVILSRKGINDTQCGFVKFFDPQCASLAMQSLAGTVIDGLEFVVRHADSKRQGEWQGGAMRTTRPLNQSWRPY
**20595**	QTLVPLMKGLDALVILTSSSPRLTQWSAWKAKVKRWLGFKKVRRTYYFPKHESPQEIDWLGQKEQIDAAKEAGVRHIVLVGSMAGTVPNHLLNTLGKGNVLRWKRKAERYLIASGVPYTIVHPGGLLDSAGGRRELVVGVDDQLLNTKFSTVPRADVAEVCLESLRTEEAKFRSFDLGSRPEGEGNPWDGNLKSRLRRRQVVRSHFLAPSMVVVLTSIEGYFMPIGRYLFPPEILIKAGSQINIILIPKRGIFRSFTTSPPPALPPF
**20996**	LVAWWSRSNPKDYAKYVQKYGEPEFMPGLAETDDYEAQIMEALGRAQAAAPAPTAAHPAVAAAASSDAADEEEEGKDLPAAVRQVQRDWRRELDHLSDQARAEEELLRLRHLQDMEEARAAGVSEEMLHNLRREHSEELSLMREQNRLLKHEIVRQKQKEEVKALLLSAKEANDVSTGRPTAADALAEQRQQEFHRIAEIVEHLQQTCDTKDEVLNRMFELICYYEATINDYDVNVVAIAAAHGGAADADRQLAEARKREEELRTLVFLQNEELEALRKAAAAAPPPSSPRALAGGLDALWLRAMAAPPSAASTE
**21028**	MHLTATGEAIPSEGSQGETLAMPSSATLSGRGMFTAGVVMSFSAAAAVASLWLFRPASETVSFAAVSGRRSMELRATLNCRLVPRDEPGYRYLGSTEIPSPVPRHELVDSIQQWAANELKEQLEAKHFGFRMVVDYEDDRVVEGDTFSLTVRFIAPTDEYIVLEYCLDNETVKVARTIEGFKVEDKERQILGRDGTPDVRSISLLGTESEVSGRFFMVRRKEGPMPDVLRPMVSDFLKETAMALTRYYAFG
**21133**	PECFQTPYGTSFFPEYAEDVPGGETSQAMRKAAIDHKITLVAGSIPEKQDGKVFNCSVAWGADGALLGKYRKMHLFRLNTEKVKFDEGETLTAGDSLAVFDAGPFKFGMGICFDVRFAEFAMSYNRLGANVLLYPGAFNMVTGPAHWQLHAKARAIDTQSYVIMCSPARDPDSCSGYVAYGHSIVVDPWGDVLVEAGEEETIVFADLDPERVAAVRKQLPIESGRRHDLYSVTFTPGVH
**21286**	MAHRVLLSSDVVPEVYDLSLEPDLQKHTFSGEVVVHCDVRVATDVVTVHSRDLAISAASFAGEGSPALDAAEIVVNKPLTTATFKFAEPLPTGKGQLKVHFIGTLNDQMAGFYRSNYTRANGQPATMAVTQFEPIDARRCFPCWDEPAQKCVFNVTLTIPRDLTALSNMPESREEFVEGDRKRVTFLPTPKMSTYLLAFCVGEYESMSSLTKDGVLLRVFSVPGKRASCAYSLECGVKALEFYNEFFGIPFPLPKMDMVAIPDFAAGAMENWGLVTYREVDMLCDLATVAVARKQRICAIV
**21679**	MPGRREDKIWNGFNKKKADGKQYFVGECKGCNEVVAGLTDRLKQHASECSKLQELNIWGDAPAAKRPKVQKQLIFGATTIKNQKQGSPFTQEQEEDMLKCKVCEKMMEEVHLKMATTPGGAEIKGHRLDKNNKQVIKEQARIAARAIEILEDVCKSPKFDEFGTMGRTVCEDALQESDEELEQYVKANVESKAFATKVCRKKCAMKSELSDMIERMKASMPPTPVAKNLTFWDELQEAAAIVQGQWKLVVLASTCLTVLVAALRIAYDVRQERRQKKAD
**22079**	MPVAPATAALKAAESVAAKNALFMTDLNKPAKVTKKDIERYAAQLDGEVAKMRAQVTEELNERYRKAVAETNTLQDVKKELSVVDQIIAVDVAVIREKIDTATGELHDAQKRFDAAEQEFIAAKLEIKMCEEYKNELTEYLTLLIQENQKQKTAKLEELSAALHSAGKLTEAGRQVLDMAKESAAVAAESAKAQAASVSAAADALLPPRSSSANHSFNDTPDDAAPDA
**22401**	MAGFFASILAWFRSLFWKQEMELTLVGLQAAGKTTLLNVISEGRHAAEKDTIPTVGLNTRKVTKGNVSIKLWDIGGQPRFRAMWERYCRGVNAIVFVVDSADSNSLGAATTELHELLSKPALAGIPLLVLGNKNDLPNALKVQDLIDAMDLKSIKGREVCCYSISAKNEVNIDMTLEWLIKHAKKG
**22409**	GGSHVDFAKLLYSFADQVENKKFEVYVEDFKKLDSIIAEKGPFWAEEKIFQSPTFQGLSEGFKFILGWIQSEGAIDRLENVRLAYKELVNEARKETTATVIVAKEPSGNDLAEIRKQVEELHKESPLKDYKLVLETKVACPPHGGRSGFWFPC
**22467**	MRLTAELIERVPQFTNPLKERELDLRGNKITQIENLGITEDGFDTIDLSDNEITKLENFPLFHRLKTIIAHNNRLCRIAAGLGKVLPRLETLALHNNHFKELSELEPLTDLPNLLRLSLCDNLVTKKPNYRLFVIAKCPKLKHLDFRKVKPKERKQAEDMFGQKKEDAAAPKAVANGDVVAPAVILSAEEQQRIMQQIEAATSYEEIQKLEERLLAGTAQ
**22652**	MDSSRNESTQWSTTAIVCVTALLSVCVGATAGFIASQPSTNLYAPTAAVKPINIAPLPVSSAARAPLPLHAVRVPQDGQILEDSMFTTSSSEANQGQPWLTCALAGLAFSAASLLTFLRKPKTEENYAMLAEIATDPKEDKRYRTGPWPEKGFELSGRVISTTMDKTAVVLVERRILDTKYHKFKRVTKKYKAHDEFNEYKNGDEVVIRLSRPISKTKSFVVIGKLLK
**22662**	MYGTVSASSPNKATSTVAVCACAAVFAVAGFVAVQPHAHYTPAAVRPAVQTTMTTIPAPRSFQANIVSATQMQSQSQQMESSVFSQIGAMSVPIFSLGFGAVAGALGGRLALKLFSSSGASPELFEKVRSIVIKELGIDEEKATPDANVQELGADSLDLVELIMKLEDTFGISIPEEVSMKITTIREQGCTGIFTKDYSHLATSLGHLR
**22828**	EDEARLQAVAEALLEVKARIAAVSARQSRPREPRLVAVGKLHGPELIRACYDAGHRCFGENYVQELVDKSSSLPEDIEWHFIGHLQSNKAKQLVEGCKGLATVETVDSDKLAAKLSAAVQALGRGPVRVYIQVNTSGEESKSGVEPGQEVPLAEYITRECPGLIFAGLMTIGMPDYTSRPENFQCLQQCRARVADRLGVPPDSLELSMGMSGDFEAALEMGSTNVRVGSTIFGARPKK
**23031**	MPPLGRPPLPGGPGALRLALRQFRQVGHRVHPLAFDFGLDVYLRGYERPRVERAAHDVAKFVQDVVPPALEQRRRWVERCYLAPPVHCITVPQEVVDEPNIQAYARQEAETLQRLLDHAIREAQQCAPVKCKTAAMVWTVLSSPFKYKRHFQKLDEVIYTAKVSFNMPMRDEALEERFAAIPGWLPIGVEAQVVTRRVENFTCPLTLEGQKDSTGGLSLLEKYCGP
**23275**	MSHSQEVYSIFRVFKTLFEMLHDRGYLVTSEDLQLTVERFKQEKCILEGEGDQAQFRLKPRNSLWLLHRKLSDTSDGIFVFFPDEVKMGVKPIRQYHDRMVEEKIKRAIVVTQQAPTPFAKQAMAALTAQGMIFETFGENELMVNVTKHELVPKHEPLTDEEKKEFLRTHKLKEKDLPRIQQSDPVSQYFGMSKGQVFRITRKSETAGTYITYRLVM
**23444**	VTSLSRRGPPDAPQLASQEWVRRVEWLKGDVLQRDWRTHLSPDVVAVVSCVGAFGSNDVMEKVCGDTNIAACEEAAAAGVPRFVFVSAHDYHGIGHGVGPLRGYFNGKRRAERRVQELFPQTGHILRPSFIYGTRHAGGYALPLGLVGAPLAAVCALPPVKFLATLPVLDLAFTSPVAVEAVARCAVQAATGKLVKPILDVDDILRA
**23771**	MFRTRMLLTFNAKFFKDRFDLQLVVPPVWTGMSAERDWQQYPLHFVVAVGNQKDIADALYNVSKFTGQCGRDSLPEGHVSVNDWHRDTLKTPLDFAVHRADPEIVSLLLKRGARPSPLTIRLAEELVQDVQDPTYAERMHQTLQKVHSQDEAEAVLKLVSDAYRNKPLHDVPIAGPPVGW
**24159**	GLPPGIDPAALANNPQVLAMLQQMLQAQGQQPPQLSPLAQALQAIPQFDQLRQVIASNPQMLQAVIQQLRTSNPQLFQLINQNPQEFIQLLQNGIPSGAGAAGLPAAPGAGAGGVPPGVIQVTPEEKAAIERLAALGFPERVAAEAYLVCGKNEELAANFLFENGAMDFDDGAEDEGGN
**24336**	MTEDPDQDVAFRIMLRLPKGHDRRQRPMDLQLWFTEDRKTIGYVLFDLTASASALWGMSVNPPRRRTGLASLFVSLWLQLCICWGVEPRTRRIDKPLLSLVLQRFGFTPLSSGIALEVAPPGAGPATVVWAEDAERLRSVFSWRYLRSQNLEIAAQRPVGGTPVCVNTAYAAPDAEVWQTCVARLVAGRVRLHGPAPGGPLKGPGDLFRLLAEVDPSLPTHYLQHLALHSRGDA
**25106**	MNNNRQGLPPAPPKTSGGKGKSYDDEDGKNRRRPTSRSMKAGLQFPVGRIHRRMKDSLISGQRVGATAAVYMAAILEYLGAEVLELAGNASKDLKVKRITPRHLQLAIRGDEELDTLIKATIAGGGVIPHIHKSLIQTPKSGPSKKKMPTEENP
**25253**	MTDLDQRSLEYASEVSNLRDKLQNIVVSGRSKDGAVSVQYWGTGLVSQLKVKEDQTDLNAVLSGIQQAINDGNERLCQEAAAEQQAFDEVWQSMFASLAQKEALQPYFPSIKRTLQGERYEQAVQRRMRQYLGPLAGPGAAAAGEPPAATAADAGEEKAEAAPPAPKEPREPKAEAQAPPSADGASSTPSCADGPPLAA
**27401**	INGSWAIHQDATGRAKDRTLSLAMGVGCGHLFETTFEKEVSSDLTGERCVLMGMLQGAFKAQYDVLRAAGHSPSEAYNETIEEALCSLYPLVAEKGMDWMYANCSTTAQRGALDWAPKFEAALKPVIQDCYNEVLAGREAARSISKNSEPDYREKLEAELAEIADQEMWVAGRLLRPLRPENSKK
**28113**	ADAKQREIQERLARLAKLTGQTVPAAPASAPVSPRAAPVSYAAPASSEWERRQQDREFREREKKEQAQREIQERLARLARLGSTPPAQPAVHAVASGDSELERLRIQREQRERREREAAEAKKREIQERLHRLQRLSAQSAAAPAYASKY
**28941**	MHAAALLLLCLLPFCAANSRVAHLLVLKTLNKFEVVGDEDVSVTINVFNVGKSAAVSVEVDDTAWADKVVQVKGSLATSIDKILPGYNYTHEYVAKAVAPGTLPVGATKVTYHSDPKEDDSQGKVVFSNILPALPIFTKAEYNKRTTRHLREWFTFFVLAFIPVGLPAVMYFTARKKLQELDEKLNPPAPGAKGKGGKKA
**29430**	MPLTKADHGTTILAVKGEVLELTLVGNPTTGYQWLPARKLPENSELKDLGSTYKVDEPELCGSPGVYTFKYEVQGPGTIALEYRRDWEGAVPALEVFEVQVALK
**29936**	MFVSGGFLLMTGVLYGLLKKPLAPQSSITLLAATAEAPEQVNVKDSMKHMMTSQGFYEAYELVMLISPRLSDLEKEDRLASLEVLFTQHKCKKVEKTDRGRRILAYPMKGAVEAYVIIYHFKGPRSMPKFITEWHTGPGLNSDGNVLRCSLMKQQLLKKAESVKPLPPIEDEPPMWSV
**30241**	AGCGLEIEGKALTALGAHWHTQCFTCSKCGDAIASSGERSLCALENQRPVCAKCRTSHACAACGEKIQGQVMTALGKKWHPSCFQCGKCKEKITGSFFHRDGVPYCSSQCFQ
**31431**	DISGQSIYFRMIDQYVQDKARPANAPPEPGATSVLENTSVCAVVYDVTNKDSFDKCMKWLEVCRKQRKGMTAVLIGNKADLVERQVVSPVVAEGFARAHGMEFFQLSSLQRSEVEQPFQHIAHAFYRSYEAKLKALGQVE
**32126**	VQATMTTVPVAHSVQSNTVAAQIQSRPLNVESNVFAQVGSTFAPIALGVGALAAFVAALIHKGQQPVAMAASSGERLLWIPNATPPAHLTGEFPGDRGFDPLGLSKDPKVFARMRISEVFHGRLAMLGIVGCVGQEWLFNKGAWFDYSDFDLPRLGLIALQVIAPLEYWRGNGGFSWNGNDGPDRSYPGFDPLGLTTEDTKLRE
**32885**	MTKGTTSFGKRNGRTHKLCKRCGKRSWAVQKKRCAACGYPNPKMRSFNWSEKAKRRNTMGTGRMRHMKNVLKKAAVRQRQDQVAPHQKRKTAENRKKFALSRKTKLAKDAKKA

**33811**	MGAKGAKPQYATPYGAYGPRVGQIPNPAVRGYPRPAPAAYPAPAPVYAPPAPVYAAAPTYSYAAPVTSYAAAPVTYAPSYAPAPVTSYAAPVTSYGYAPSAVAAPTQGAAFALDAADGVIDGRYYGSQVGVTSPAYYGY
**34869**	ADPIFNGSKVVRSIQMMRSYHRLPVGPEPPHLKIRGAPLHPWSMYTDKGGFFFGVGTQLPRNFFPKFLATTSAIMGVTYGLVWLYNAAGPRAKTQTRKWKELESEDPRFPFQEIPEIYLPSDIDRDPNADCWRILNQVPRKEKLLVEITDPLRNFDFKVAKIKEN
**35001**	LIYAALNDPEAPGRAVNWAGFYLHRGGDDLVLGPFQGKVACIRIRKGRGVCGACWAEGKAQLVPDVHKFPGHIACDSVSESEVVLPLTDKSGRFLGVLDLDSPVKDHFDAVDVDGLGRIATLLGNGCDWSPVAPAVTTGLQ
**36724**	KDITAALNDVAKFTGQCGRDSLPEGHVSVNDWHRDTLKTPLDFAVLRGDPEIVSLLLKRGARPSPLTVRLAEELVQDVQNPTYADRMHQTLQKVHSRDEAEAVLKLVGDAYRAV
**48568**	EAAKTSFRISMALFAPSYTEQSYVPPQKEKVDVVLEGVFQKIDLVGNQLCATAAELEELKAEIGNSNTILDRELRTQLQQANDEIYRLRNQLSEYTDRKK
**49190**	MAAACAVRGFTTARPMLTPNKVKVPGRKPQDEEDLTWAEADRKLTPEERYARDKQMALLDKMTSQVEELEKSHTEQKKSNKGVKAQIEAISRQLEALKAQLKE
**49725**	FYEEPETLFDKNGRPAYPKATGPGYAPYRDPLPELPTVYNKSGKPIKPKGVPPGYVPPGANFPPVPKGTQMVNGPKGPTLLCKSKSIANVCFMSQCPP
**50778**	AAWSLPSPRPALAATGPRVLVAGATGATGKLTVQALKAQGADVVAGVRNVAKAASLGVPAVPLDITGRFDALVDSLKGVDVVVSCIGFTPGNPFEFSKAAHAVDNVGTVALVDAAKAAGVKRFILMSSIL
**52389**	HEIRQLEKKDLLKQLDDLKTELAQLRVAKQTSGAASKLCKIKIVRRSIARVLTVLNMKEKNTLRKLYKNKKYKPLDLRPKKTKKERLALSIIDRRRKTARQKKILHAYPMRKYYVKV
**52992**	MSTFKDSNLLFPPEASEKRKHKLKRLVQGQNSFFMDVKCPSCWAMYGCNLWNNFSFLFFCSTIVFSHVAKVVVCSSCNTVLAQPTGGKVCFSLSSINVTPFNYFRQSSLKVAPSARSQNE
